# Incidence and factors associated with postoperative delirium after primary total joint arthroplasty in older adults: a systematic review and meta-analysis

**DOI:** 10.3389/fmed.2025.1664605

**Published:** 2025-10-22

**Authors:** Yandong Ni, Xu Yang, Yuelai Yang, Huachun Zhang, Sheng Peng

**Affiliations:** ^1^Department of Nursing, Shanghai Ninth People’s Hospital, Shanghai Jiao Tong University School of Medicine, Shanghai, China; ^2^Department of Anesthesiology, Longhua Hospital Shanghai University of Traditional Chinese Medicine, Shanghai, China; ^3^Department of Nursing, Longhua Hospital Shanghai University of Traditional Chinese Medicine, Shanghai, China

**Keywords:** incidence, factors, postoperative delirium, total joint arthroplasty, meta-analysis

## Abstract

**Background:**

The proportion of older adults undergoing total joint arthroplasty (TJA) is increasing annually. Postoperative delirium (POD) is a common and serious complication among older adults after surgery. However, the incidence and factors associated with POD following primary TJA in this population remain unclear.

**Objective:**

This study aimed to assess the incidence of delirium after primary TJA in older adults and to identify factors associated with POD through a meta-analytic approach.

**Methods:**

A systematic literature search was performed in PubMed, Embase, Web of Science, and the Cochrane Library for studies published from inception to June 2025. Observational studies reporting POD incidence following TJA, including total hip arthroplasty and total knee arthroplasty in older adults (aged ≥60 years), were included. Pooled incidence rates and factors associated with POD were estimated using a random-effects model.

**Results:**

After applying the inclusion and exclusion criteria, 35 studies involving 29,311 older adults undergoing TJA were included. The pooled POD incidence was 13.6% (95% CI, 12.2–15.0%), with substantial heterogeneity across studies. Advanced age, sleep apnea, hypertension, diabetes mellitus, coronary artery disease, stroke, chronic obstructive pulmonary disease, renal disease, solid tumors, dementia, Parkinson’s disease, psychiatric disorders, ASA class III/IV, substance use history, and blood transfusion were associated with increased POD risk. Conversely, higher educational attainment was identified as a protective factor.

**Conclusion:**

This study systematically reported POD incidence among older adults undergoing TJA and identified factors associated with POD These findings provide evidence to optimize perioperative management and develop prevention strategies for POD in this population.

**Systematic review registration:**

This study was registered in INPLASY platform (number: INPLASY202570015).

## Introduction

With global population aging, the proportion of older adults aged ≥60 years continues to rise. Consequently, the number of older adult patients undergoing total joint arthroplasty (TJA) for joint diseases such as osteoarthritis and rheumatoid arthritis has increased markedly (1). Statistics indicate that in Europe and North America alone, over one million older adult patients receive hip or knee replacements annually, with this number growing at a rate of 5–8% per year (2). By reconstructing joint anatomy and function, TJA has become a critical intervention to improve joint mobility, alleviate pain, and enhance quality of life in older adult patients (3). However, postoperative delirium (POD), a common neurological complication following surgery in older adults, is increasingly recognized as a major clinical concern (4, 5).

POD is a syndrome marked by acute confusion, cognitive dysfunction, and inattention. Its pathophysiology involves central nervous system inflammation, neurotransmitter imbalances, cerebral hypoperfusion, and oxidative stress (6). In older adults, reduced central nervous system reserve, increased blood–brain barrier permeability, and frequent comorbidities such as hypertension and diabetes substantially elevate the risk of developing POD compared to younger individuals. Research indicates that delirium is linked to a 4-fold increase in mortality, a 2.4-fold rise in healthcare costs, prolonged hospitalization, impaired functional recovery, and strong associations with long-term cognitive decline and higher dementia risk (7–9). Moreover, the occurrence of delirium significantly increases healthcare resource utilization, imposing a considerable burden on patients’ families and society.

Despite the substantial impact of POD on the prognosis of older adults undergoing TJA, considerable inconsistency persists in reported incidence and associated factors across studies. Reported POD rates following primary TJA in older adults vary widely, likely due to differences in study population characteristics, surgical procedures, delirium diagnostic criteria, and perioperative management protocols. Additionally, most studies are limited by small sample sizes and a lack of multicenter data, with risk factor analyses often restricted to univariate approaches. These limitations hinder a thorough understanding of the associations between patient-related factors, surgery-related factors, postoperative management, and POD occurrence. Therefore, conducting a systematic review and meta-analysis to comprehensively assess the incidence of POD following primary TJA in older adults and to identify key associated factors is of significant clinical importance for optimizing perioperative management and developing targeted prevention strategies.

## Methods

### Data sources, search strategy, and selection criteria

This review strictly adhered to the requirements outlined in the Preferred Reporting Items for Systematic Reviews and Meta-Analyses statement (10). Our study was registered in INPLASY platform (number: INPLASY202570015). The study aimed to comprehensively analyze the incidence of POD and its associated factors in older adults following TJA. To achieve this, we systematically searched epidemiological studies without restrictions on language or publication status to capture global evidence. We systematically searched multiple electronic databases, including PubMed, Embase, Web of Science, and the Cochrane Library, for relevant studies published from database inception to June 2025. The search strategy was designed in consultation with a professional medical librarian to ensure high sensitivity. It combined controlled vocabulary with a comprehensive list of free-text synonyms and key phrases for the concepts of (1) total joint arthroplasty and (2) postoperative delirium. Boolean operators (AND, OR) and field tags (e.g., [tiab], [mesh]) were used appropriately. The full electronic search strategy for PubMed is provided in Supplementary File 1. In addition to the electronic database search, we manually screened reference lists of included studies and review articles to identify records potentially missed due to indexing limitations or oversight. Our search was focused on electronic bibliographic databases as they are the primary repositories for published observational studies, which constituted the target evidence for this meta-analysis. Clinical trial registries were not searched as they are designed for registering interventional studies, which were outside the scope of this review.

Literature search and study screening were performed independently by two reviewers with clinical epidemiology backgrounds, following predefined standardized procedures. Disagreements during screening were resolved through discussion; if necessary, a third senior researcher was consulted until consensus was achieved. Studies were included if they met the following criteria: (1) Participants: older adults aged ≥60 years undergoing primary unilateral total hip arthroplasty (THA) or total knee arthroplasty (TKA). This age cutoff was chosen to be inclusive of the widely accepted definition of “older adults” in the surgical literature and to ensure a sufficient number of eligible studies for analysis, as many relevant studies define their cohorts starting from age 60; (2) Group setting: clear distinction between an exposed group (patients developing POD) and a control group (patients without POD); (3) Outcomes: reporting of delirium incidence and identification of potential associated factors; and (4) Study design: eligible epidemiological studies, including prospective or retrospective cohort studies. Exclusion criteria included: (1) Ineligible design: case reports, case series, reviews, meta-analyses, animal studies, or basic research; (2) Ineligible surgical characteristics: studies involving non-primary arthroplasty, simultaneous multiple-joint arthroplasty, or non-target orthopedic procedures; (3) Ineligible patient characteristics: patients aged <60 years or those with severe cognitive impairment or psychiatric disorders; (4) Insufficient data integrity: studies with unclear delirium diagnostic criteria or missing critical data unobtainable from authors; and (5) Language and publication status: We restricted inclusion to studies published in Chinese or English due to practical constraints in translation and critical appraisal. Publications in other languages, as well as conference abstracts, unpublished preprints, and dissertations, were excluded.

### Data collection and quality assessment

Two reviewers independently extracted the following data from included studies: first author’s surname and publication year, study design, country of origin, sample size (including POD and non-POD groups), proportion of male participants, mean age, TJA surgical site, POD diagnostic method, and primary findings. Both reviewers independently assessed study quality using the Newcastle-Ottawa Scale (NOS) (11). The NOS evaluates studies across selection, comparability, and outcome domains, with scores ranging from 0 to 9 stars. The inter-rater reliability for the NOS total score, calculated using the intraclass correlation coefficient, was 0.85, indicating excellent agreement. Discrepancies during data extraction or quality assessment were resolved through consultation with a third reviewer, who referred to the original publications to ensure accuracy and consistency.

### Statistical analysis

This study employed a random-effects model to systematically analyze the incidence of POD in older adults undergoing TJA. To enhance comparability, original data were log-transformed based on distributional characteristics (12). Restricted maximum likelihood estimation was applied during model fitting to improve parameter estimation accuracy. The effect sizes of factors associated with POD were expressed as odds ratios (ORs) with corresponding 95% confidence intervals (CIs) and pooled using the random-effects model to account for study heterogeneity (12, 13). Heterogeneity was assessed using the *I^2^* statistic and Q-test, with significant heterogeneity defined as *I^2^* ≥ 50% or a Q-test *p*-value <0.10 (14, 15). The stability of results was evaluated through leave-one-out sensitivity analysis (16). Moreover, sensitivity analysis was performed by excluding large-scale studies with a very low incidence of POD (<5%) to quantitatively assess their impact on the pooled incidence of POD. Stratified subgroup analyses of POD incidence were conducted based on publication year, study design, geographical location, TJA site, POD assessment method, and study quality. Differences between subgroups were compared using interaction tests, and data normality was assessed prior to analysis (17). Publication bias was evaluated using qualitative (funnel plot visualization) and quantitative methods (Egger’s test, Begg’s test) (18, 19). All statistical tests were two-sided, with a significance level of *p* < 0.05 for pooled effect sizes. Data analyses were performed using STATA software (version 12.0; StataCorp, College Station, TX, USA).

## Results

### Literature search

**Figure 1:**
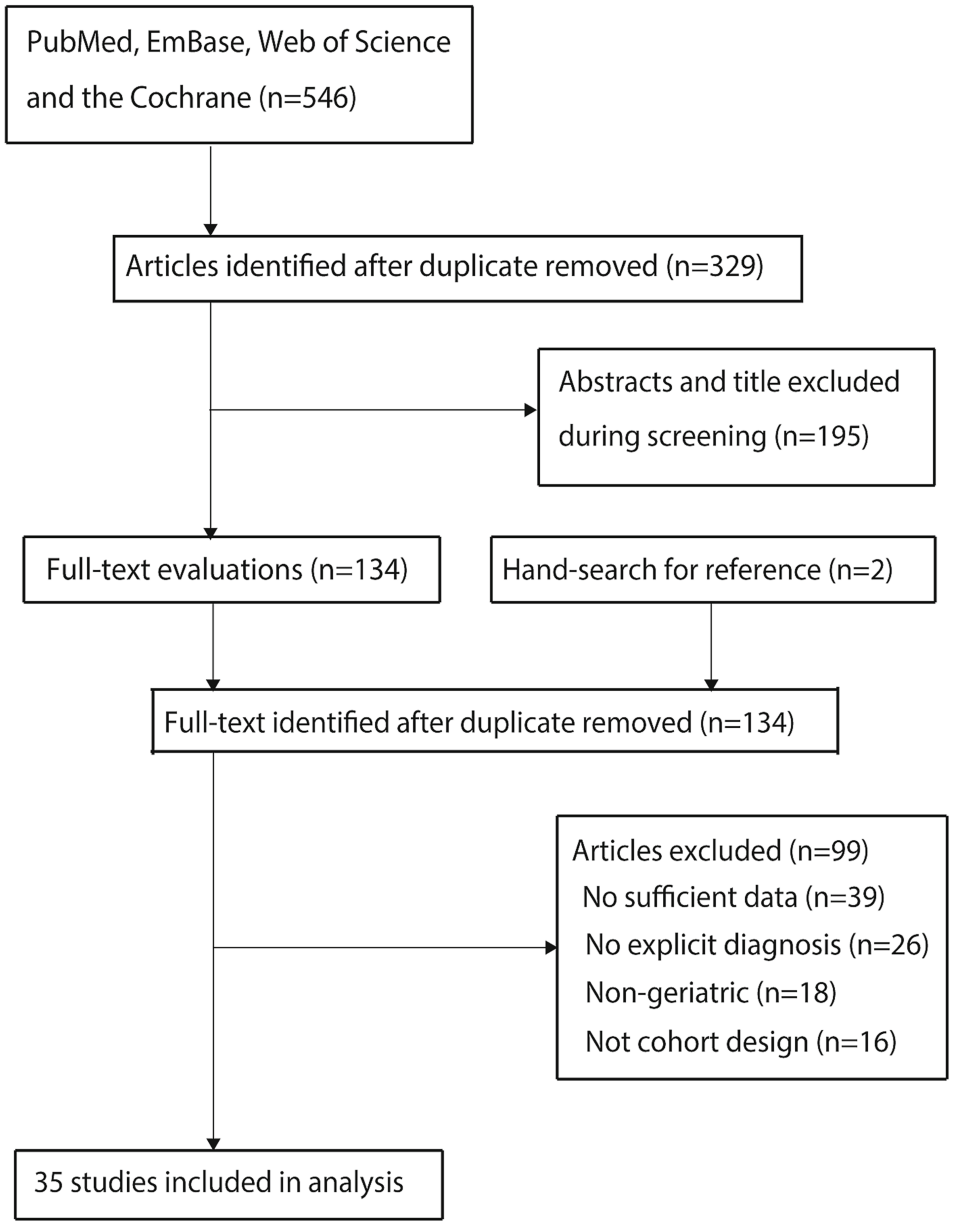
PRISMA flowchart illustrating the literature search and study selection process.

Database searches identified 546 relevant articles. After deduplication, 329 articles remained. Screening of titles and abstracts excluded 195 articles. Full-text assessment of the remaining 134 articles led to the exclusion of 99 articles due to: (1) absence of relevant data (n = 39); (2) lack of explicit POD diagnostic criteria (n = 26); (3) non-geriatric study populations (n = 18); and (4) ineligible study designs (n = 16). Additionally, reviewing reference lists of included articles yielded no new eligible studies. Ultimately, 35 cohort studies (20–54) were included in the meta-analysis. The literature screening flow is shown in Figure 1.

### Study characteristics

Table 1 summarizes the baseline characteristics of included studies and patients. The 35 studies encompassed 29,311 older adults undergoing TJA, with 1,670 cases of POD reported. Among these, 22 studies used prospective cohort designs, while 13 employed retrospective designs. Thirteen studies were conducted in Western countries and 22 in Asia. Quality assessment with the NOS yielded the following ratings: five studies received nine stars, 16 received eight stars, and 14 received seven stars.

**Table 1:** The baseline characteristics of included studies involved patients.

Study	Study design	Country	Sample size	Male (%)	Age (years)	Position	POD assessment	NOS
Rogers et al. (20)	Prospective	USA	46 (13/33)	32.6	69.3	Knee, hip	DSM III	7
Williams-Russo et al. (21)	Prospective	USA	51 (21/30)	45.1	68.1	Knee	DSM III	8
Fisher et al. (22)	Prospective	Canada	80 (14/66)	46.3	71.2	Knee, hip	CAM	8
Freter et al. (23)	Prospective	Canada	132 (18/114)	33.3	76.8	Knee, hip	CAM	9
Lowery et al. (24)	Prospective	UK	94 (14/80)	43.6	76.5	Knee, hip	CAM	7
Priner et al. (25)	Prospective	France	101 (15/86)	42.6	73.6	Knee, hip	CAM	7
Jankowski et al. (26)	Prospective	USA	418 (42/376)	49.3	72.9	Knee, hip	CAM	9
Cerejeira et al. (27)	Prospective	UK	101 (37/64)	49.5	73.0	Hip	DSM-IV	7
Flink et al. (28)	Prospective	USA	106 (27/79)	44.3	73.5	Knee	DSM-IV, CAM	9
Chung et al. (29)	Retrospective	Korea	365 (11/354)	9.0	71.1	Knee	DSM-IV, CAM	8
Yen et al. (30)	Prospective	Singapore	98 (22/76)	48.0	73.4	Knee	CAM	7
Wang et al. (31)	Retrospective	Korea	265 (49/216)	7.9	70.3	Knee	DSM-IV, CAM	7
Huang et al. (32)	Retrospective	Singapore	1,016 (6/1010)	18.6	67.0	Knee	DSM-IV	7
Chen et al. (33)	Prospective	China	212 (35/177)	25.9	73.8	Knee, hip	DSM-IV, CAM	7
Cunningham et al. (34)	Prospective	UK	315 (40/275)	43.2	74.4	Knee, hip	CAM	8
Petersen et al. (35)	Prospective	Denmark	6,331 (43/6288)	37.8	76.7	Knee, hip	DSM-IV	7
Peng et al. (36)	Prospective	China	272 (55/217)	42.3	72.6	Knee, hip	DSM-V	7
Cunningham et al. (37)	Prospective	UK	282 (40/242)	43.6	74.2	Knee, hip	CAM	8
Huang et al. (38)	Retrospective	USA	11,970 (181/11789)	45.0	66.0	Knee, hip	CAM	8
Kijima et al. (39)	Retrospective	Japan	170 (11/159)	18.8	73.4	Knee	DSM-IV, CAM	7
Lin et al. (40)	Prospective	China	447 (51/396)	47.0	72.3	Knee, hip	CAM	7
He et al. (41)	Prospective	China	780 (182/598)	48.6	73.9	Hip	DSM-IV, CAM	8
Qi et al. (42)	Retrospective	China	328 (68/260)	40.2	72.2	Knee, hip	DSM-V	8
Chen et al. (43)	Retrospective	China	994 (67/927)	28.8	66.7	Knee, hip	DSM-V	9
Chen et al. (44)	Prospective	China	383 (66/317)	34.5	72.7	Knee, hip	DSM-V	8
Chen et al. (45)	Retrospective	China	260 (65/195)	25.8	83.3	Knee, hip	CAM	8
Jiang et al. (46)	Retrospective	China	336 (43/293)	41.7	72.4	Knee, hip	DSM-V	8
Lin et al. (47)	Prospective	China	332 (61/271)	44.0	74.8	Knee, hip	CAM	9
Zhang et al. (48)	Prospective	China	268 (42/226)	35.1	67.1	Knee	CAM	8
Hu et al. (49)	Retrospective	China	254 (49/205)	34.3	68.1	Hip	CAM	7
Song et al. (50)	Retrospective	China	446 (79/367)	42.2	70.0	Knee	CAM	7
Chen et al. (51)	Prospective	China	294 (34/260)	NA	71.1	Knee, hip	CAM	8
Tomite et al. (52)	Retrospective	Japan	500 (26/474)	18.0	70.6	Knee, hip	DSM-V	8
Joo et al. (53)	Retrospective	Korea	973 (60/913)	13.9	70.3	Knee	DSM-V	8
Zou et al. (54)	Prospective	China	291 (83/204)	44.3	75.7	Hip	CAM	8

### Incidence of POD

**Figure 2:**
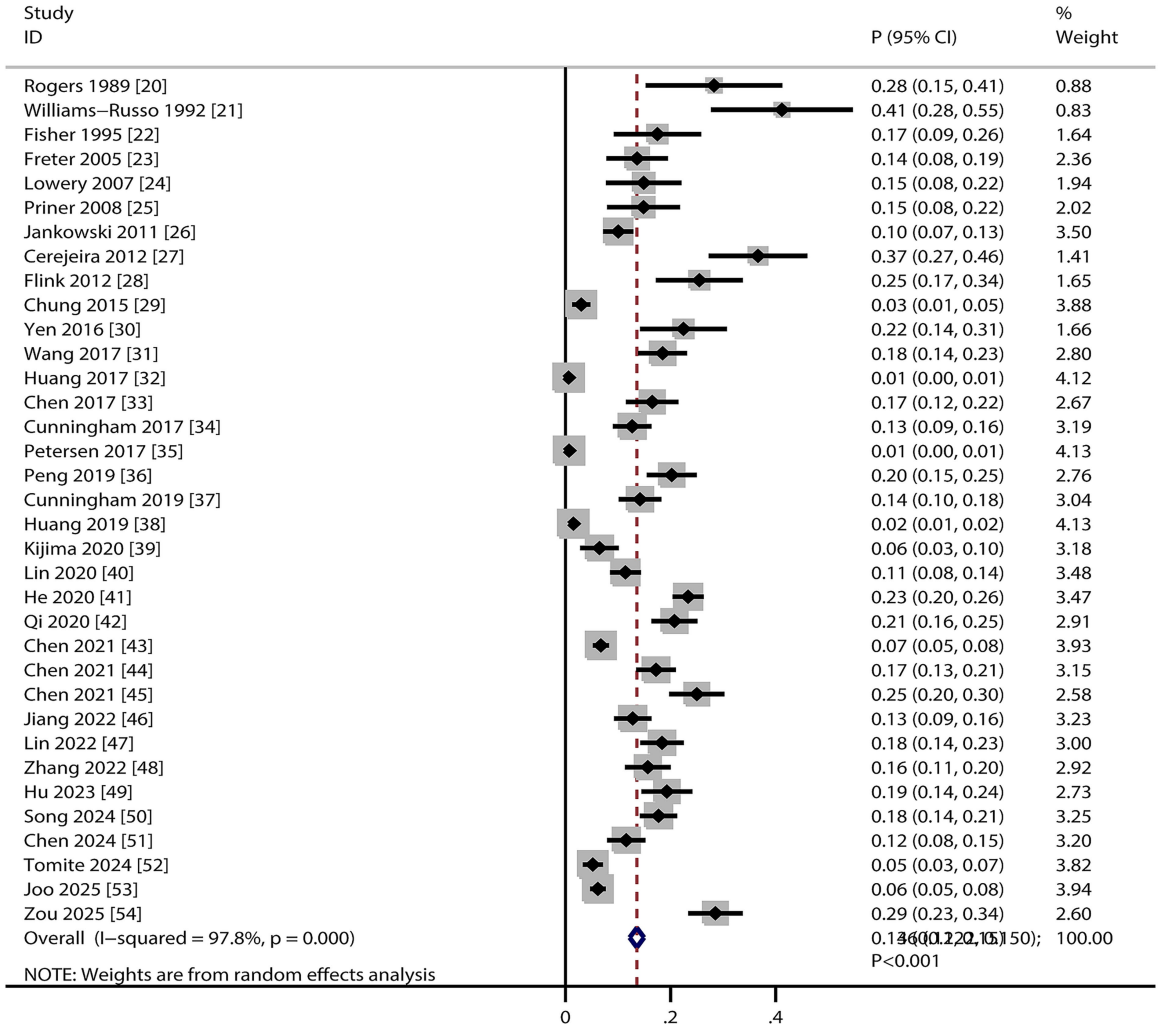
POD incidence summary in older adults undergoing TJA.

Pooled analysis revealed that the incidence of POD in older adults undergoing TJA was 13.6% (95% CI, 12.2–15.0%, Figure 2), with significant heterogeneity across studies (*I^2^* = 97.8%; *p* < 0.001). Sensitivity analysis excluding individual studies showed incidence estimates ranging from 13.0 to 15.0% (Supplementary File 2). After removing large-scale studies with a very low incidence of POD (<5%), we noted the incidence of POD was 16.9% (95% CI, 14.4–19.5, Supplementary File 2). This result indicates that the inclusion of large studies with very low event rates modestly attenuated the overall pooled incidence estimate (from 16.9 to 13.6%). Subgroup analysis indicated the highest POD incidence in studies published before 2010, using prospective designs, conducted in the USA, involving patients receiving THA, employing DSM–III criteria for POD diagnosis, and rated with 8 NOS stars (Table 2).

**Table 2:** Subgroup analyses for the incidence of POD in elder patients undergoing TJA.

Factors	Subgroups	Incidence and 95%CI	*I^2^* (%)	Q statistic	Interaction test
Publication year	Before 2010	19.8% (13.4–26.2%)	70.8	0.004	<0.001
	2010 or after	13.0% (11.6–14.4%)	98.0	<0.001	
Study design	Prospective	18.4% (13.3–23.4%)	98.0	<0.001	<0.001
	Retrospective	9.9% (7.8–11.9%)	97.5	<0.001	
Country	Canada	14.9% (10.1–19.7%)	0.0	0.457	<0.001
	China	17.5% (14.0–21.1%)	93.2	<0.001	
	Denmark	0.7% (0.5–0.9%)	-	-	
	France	14.9% (7.9–21.8%)	-	-	
	Japan	5.5% (3.8–7.2%)	0.0	0.551	
	Korea	8.6% (3.1–14.1%)	94.8	<0.001	
	Singapore	11.1% (−10.3–32.5%)	96.3	<0.001	
	UK	18.5% (11.1–25.9%)	86.4	<0.001	
	USA	19.1% (9.6–28.7%)	96.5	<0.001	
TJA surgical site	Hip	25.8% (20.4–31.2%)	78.0	0.003	<0.001
	Knee	13.7% (9.2–18.1%)	97.2	<0.001	
	Hip and knee	12.1% (10.6–13.6%)	97.5	<0.001	
POD assessment methods	CAM	15.7% (10.8–20.6%)	97.6	<0.001	<0.001
	DSM III	34.6% (21.9–47.2%)	45.1	0.177	
	DSM-IV	2.0% (0.1–3.8%)	96.5	<0.001	
	DSM-V	12.3% (8.4–16.2%)	94.2	<0.001	
	DSM-IV and CAM	15.3% (6.7–23.9%)	97.1	< 0.001	
NOS	7	13.8% (11.4–16.3%)	97.3	<0.001	<0.001
	8	15.1% (11.1–19.1%)	98.1	<0.001	
	9	14.0% (8.6–19.4%)	91.2	<0.001	

### Factors associated with POD

**Figure 3:**
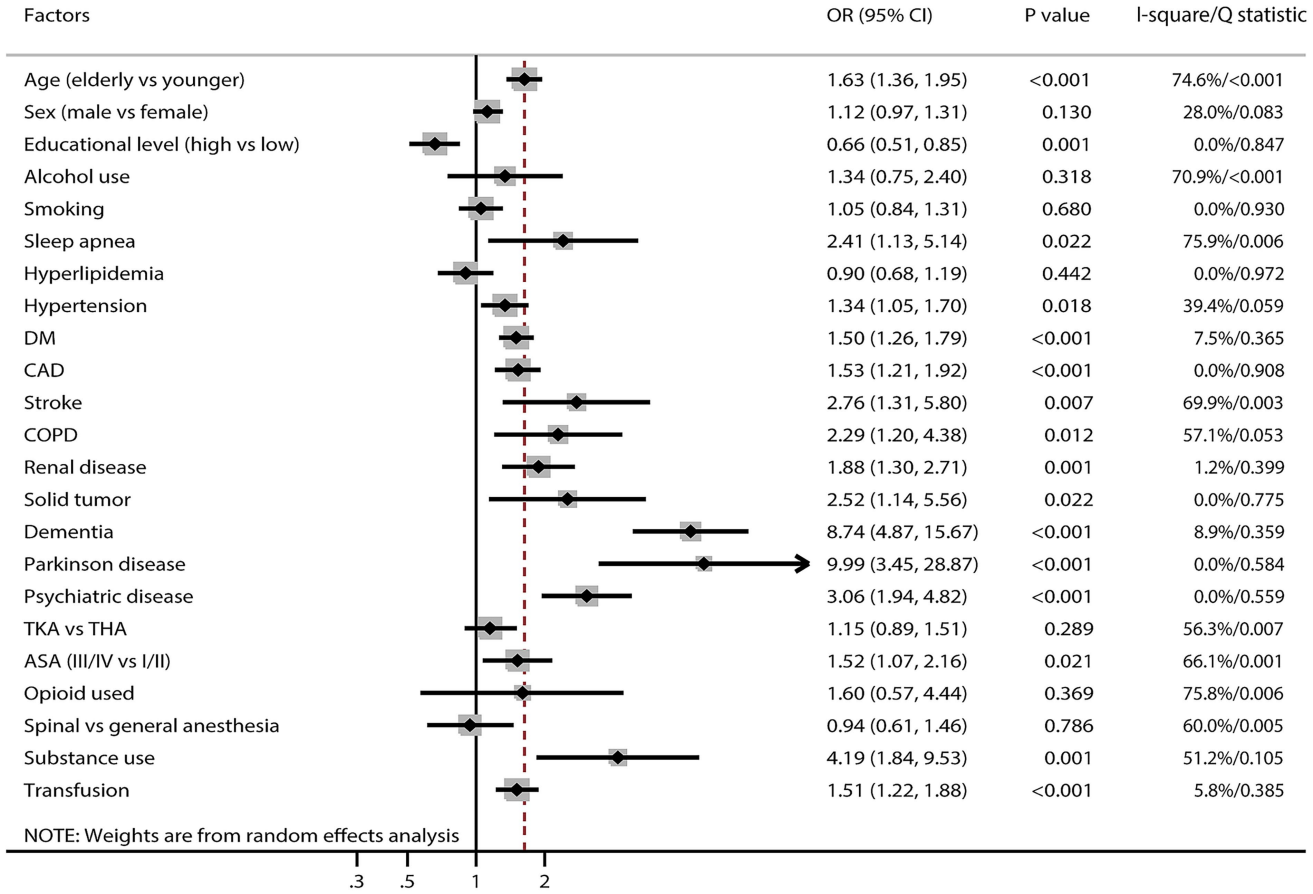
Summary of significant POD in older adults undergoing TJA.

Figure 3 and Supplementary File 3 present factors associated with POD. Significantly increased POD risk was associated with older adults (OR: 1.63; 95% CI: 1.36–1.95; *p* < 0.001), sleep apnea (OR: 2.41; 95% CI: 1.13–5.14; *p* = 0.022), hypertension (OR: 1.34; 95% CI: 1.05–1.70; *p* = 0.018), diabetes mellitus (DM) (OR: 1.50; 95% CI: 1.26–1.79; *p* < 0.001), coronary artery disease (CAD) (OR: 1.53; 95% CI: 1.21–1.92; *p* < 0.001), stroke (OR: 2.76; 95% CI: 1.31–5.80; *p* = 0.007), chronic obstructive pulmonary disease (COPD) (OR: 2.29; 95% CI: 1.20–4.38; *p* = 0.012), renal disease (OR: 1.88; 95% CI: 1.30–2.71; *p* = 0.001), solid tumor (OR: 2.52; 95% CI: 1.14–5.56; *p* = 0.022), dementia (OR: 8.74; 95% CI: 4.87–15.67; *p* < 0.001), Parkinson’s disease (OR: 9.99; 95% CI: 3.45–28.87; *p* < 0.001), psychiatric disease (OR: 3.06; 95% CI: 1.94–4.82; *p* < 0.001), ASA class III/IV (OR: 1.52; 95% CI: 1.07–2.16; *p* = 0.021), substance use (OR: 4.19; 95% CI: 1.84–9.53; *p* = 0.001), and blood transfusion (OR: 1.51; 95% CI: 1.22–1.88; p < 0.001). Higher educational attainment was associated with reduced POD risk (OR: 0.66; 95% CI: 0.51–0.85; *p* = 0.001). No significant associations were identified between POD and sex, alcohol use, smoking, hyperlipidemia, surgical approach, opioid use, or anesthesia technique. Considerable heterogeneity was observed in associations with age, sex, alcohol use, sleep apnea, hypertension, stroke, COPD, surgical approach, ASA classification, opioid use, anesthesia technique, and substance use. Sensitivity analysis indicated that associations between alcohol use and POD risk, as well as between COPD and POD risk, were unstable. Conversely, the associations of other factors associated with POD remained robust and were not affected by exclusion of any single study (Supplementary File 3).

### Publication bias

**Figure 4:**
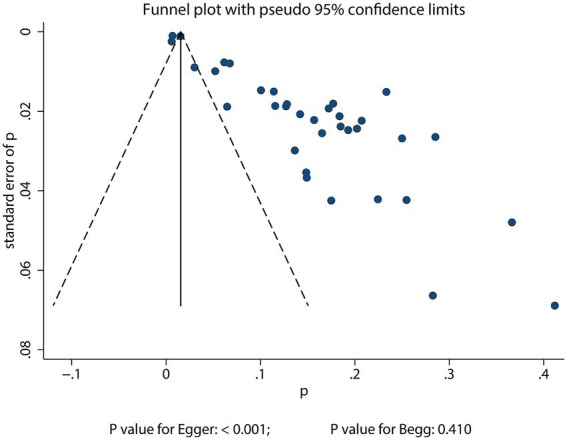
Funnel plot assessing publication bias for POD incidence in older adults undergoing TJA.

Significant publication bias was detected in the reported incidence of POD among older adults undergoing TJA (Egger’s test: *p* < 0.001; Begg’s test: *p* = 0.410; Figure 4). To adjust for potential missing studies, we applied the trim-and-fill method. The adjusted pooled incidence estimate after incorporating these imputed studies was 13.6% (12.2–15.0%), which was consistent with the original pooled incidence, indicating that the overall finding was robust to the potential influence of publication bias (Supplementary File 2). When assessing factors associated with POD, significant publication bias was identified in associations of both age and educational level with POD. Following correction, the original conclusions did not change (Supplementary File 3).

## Discussion

This systematic review and meta-analysis is the first to comprehensively quantify the incidence of POD in older adults following primary TJA at 13.6% (95% CI, 12.2–15.0%), while identifying 16 significant factors associated with POD. These findings address critical evidence gaps: (1) Clinically, the 13.6% incidence establishes POD as a common complication requiring heightened vigilance in older adults undergoing TJA; and (2) methodologically, the stratified risk factor analysis provides actionable targets for perioperative risk screening.

The most significant finding of our meta-analysis is the considerable heterogeneity (*I^2^* = 97.8%) in the reported incidence of POD following TJA. This degree of heterogeneity indicates that the included studies are not estimating a single true incidence rate but rather a distribution of rates across diverse populations and settings. Therefore, the pooled estimate of 13.6% should not be interpreted as a precise figure but as a weighted average across a highly variable evidence base. Our subgroup analyses offer important insights into the sources of this variation. The analysis by diagnostic criteria was particularly revealing: studies using prospective, active screening with the DSM III reported a significantly higher incidence than those using retrospective administrative data. This underscores that a major driver of heterogeneity is methodological, related to how delirium is identified and recorded. Beyond diagnostic methods, we hypothesize that the residual heterogeneity stems from clinical diversity that we could not fully explore with aggregated data. This includes differences in perioperative protocols, patient-level factors, and healthcare systems. The high heterogeneity precludes a simple, one-size-fits-all application of our findings. Instead, clinicians should contextualize the 13.6% estimate: it likely represents a minimum risk in a typical, heterogenous cohort, with the true risk for specific subpopulations being potentially higher.

Previous meta-analyses reported a 3% POD incidence across 23 studies, but those findings were largely driven by several large database studies with potential patient overlap (55). Another meta-analysis identified advanced age, dementia, hypertension, diabetes, stroke, psychiatric disorders, and sedative-hypnotic use as POD risk factors; however, incomplete coverage of relevant studies limited its scope (56). These gaps necessitated the present investigation.

Our cohort-based meta-analysis establishes a 13.6% POD incidence, with variation attributable to three primary factors: (1) diagnostic heterogeneity, as we integrated studies using rigorous criteria (e.g., DSM-III) with those employing validated screening tools, enhancing generalizability; (2) age stratification, as our strict inclusion of older adults aged ≥60 years contrasts with prior analyses of broader age ranges; and (3) procedural and regional variation, with higher POD rates after THA versus TKA, likely reflecting greater surgical trauma and blood loss in THA. Moreover, the interpretation of the pooled incidence rate of 13.6% should consider the influence of study size and event distribution. A sensitivity analysis revealed that large-scale studies reporting a very low POD incidence (<5%) modestly lowered the pooled estimate. When these studies were excluded, the incidence increased to 16.9%. This finding highlights the heterogeneity in reported POD rates, which may stem from differences in clinical settings, patient populations, or, most importantly, the intensity of delirium monitoring. Studies with prospective, daily active screening typically detect higher rates than those relying on retrospective administrative data. Therefore, our primary estimate of 13.6% is a robust average across diverse settings, while the sensitivity analysis (16.9%) may better reflect the risk in closely monitored clinical cohorts. Finally, elevated incidence in Western compared to Asian studies may reflect differing perioperative protocols, including pain management and transfusion strategies.

When examining factors associated with POD, advanced age impairs cerebral blood flow autoregulation, rendering the brain more vulnerable to hypoxic insults from postoperative hypotension or anemia. Simultaneously, increased blood–brain barrier permeability heightens susceptibility to neurotoxicity from inflammatory cytokines (57). Cerebrovascular diseases such as hypertension and DM contribute to chronic cerebral ischemia (58, 59), while conditions including CAD and COPD exacerbate oxygen supply–demand mismatch, establishing an “ischemia–inflammation” cycle (60, 61). Patients with dementia or Parkinson’s disease exhibit deficiencies in neurotransmitters, permitting surgical stress to trigger delirium (62, 63). Long-term medication use among psychiatric patients may alter central nervous system sensitivity (64). Additionally, blood transfusion may induce cerebral injury through immunomodulatory effects or microemboli, and patients classified as ASA III/IV possess reduced physiological reserve, limiting their ability to tolerate surgical stress (65).

The most important limitation in interpreting the factors associated with POD is our reliance on univariate data, as highlighted by the reviewer. The pooled odds ratios represent unadjusted associations and are therefore highly susceptible to confounding. For example, the strong association with advanced age is likely confounded by its correlation with higher comorbidity burden and reduced physiological reserve. Similarly, the link between hypertension and POD may be mediated by its association with underlying cerebrovascular disease. The substantial heterogeneity observed for many of these associations further underscores the contextual nature of these relationships; their strength varies depending on the specific patient cohort and clinical environment. Therefore, these findings should be viewed as hypothesis-generating, identifying candidate variables that are clinically relevant and must be validated as independent predictors in future prospective studies using multivariate models. They are invaluable for building predictive models but should not be used in isolation for clinical risk stratification.

This study has several limitations. First, the restriction to Chinese and English language publications may have introduced language bias, potentially excluding relevant studies published in other languages and affecting the generalizability of our pooled estimates. Second, inclusion of both prospective and retrospective cohort designs may introduce selection and recall bias, affecting representativeness. Third, heterogeneity in POD diagnostic criteria may influence pooled estimates. Fourth, factor associated with POD were based on univariate approaches without adjustment for confounders. Fifth, despite our efforts to explore heterogeneity through subgroup analyses, a large proportion remains unexplained. This is a fundamental limitation of meta-analyses of observational data, where unmeasured confounding and clinical variation are inherent. The results should be interpreted with this caution in mind. Sixth, the age cutoff of ≥60 years, while chosen to maximize the inclusion of relevant evidence, may have diluted the analysis by including patients aged 60–65 who are at a relatively lower risk compared to those over 70. Future studies with access to individual patient data would be valuable to perform a more granular analysis of delirium risk across narrower age bands (e.g., 60–69, 70–79, ≥80 years) to better define the age-related risk trajectory. Seventh, an initial limitation was the lack of a pre-planned sensitivity analysis based on study quality and the reporting of inter-rater reliability for the NOS. However, these have been addressed in the revised manuscript in response to peer review, strengthening the analysis. Eighth, our meta-analysis incorporated studies with a highly skewed distribution of POD cases relative to sample size. While we conducted a sensitivity analysis to address this, the pooling of such heterogeneous event rates remains a methodological challenge. Finally, our study is subject to the limitations inherent in meta-analyses of observational data. Although our search strategy was comprehensive for published literature, it was restricted to bibliographic databases. We did not search sources of unpublished data or clinical trial registries. While these registries are not a primary repository for observational studies, their exclusion, along with that of other grey literature sources, means our results may still be susceptible to publication bias.

## Conclusion

In conclusion, this meta-analysis demonstrates a clinically significant incidence of POD in older adults undergoing TJA and identifies numerous patient-level and treatment-related factors associated with its occurrence. However, due to the reliance on univariate data and the presence of substantial heterogeneity, these associations must be interpreted with caution, acknowledging potential confounding. Future research should focus on validating these factors as independent predictors using individual patient data meta-analysis or well-designed multivariate prospective studies to enable robust perioperative risk stratification.

## Data Availability

The original contributions presented in the study are included in the article/Supplementary material, further inquiries can be directed to the corresponding author.
