# Supplementary material for: Incidence and factors associated with postoperative delirium after primary total joint arthroplasty in older adults: a systematic review and meta-analysis
Source: Front Med (Lausanne). 2025 Oct 22;12:1664605. doi: 10.3389/fmed.2025.1664605 (PMC12586022; doi:10.3389/fmed.2025.1664605)
Supplement: Supplementary file 1 [file Table_1.docx]

**Search strategy in PubMED:** ("deliri*"[Title/Abstract] OR "confus*"[Title/Abstract] OR "POCD"[Title/Abstract] OR (("postoperative period"[MeSH Terms] OR ("postoperative"[All Fields] AND "period"[All Fields]) OR "postoperative period"[All Fields] OR ("post"[All Fields] AND "operative"[All Fields]) OR "post-operative"[All Fields]) AND "cognitive disorder"[Title/Abstract]) OR "acute confusional state"[Title/Abstract]) AND ("risk"[MeSH Terms] OR "risk"[All Fields] OR ("predictor"[All Fields] OR "predictors"[All Fields]) OR ("factor"[All Fields] OR "factor s"[All Fields] OR "factors"[All Fields])) AND ("knee"[MeSH Terms] OR "knee joint"[MeSH Terms] OR "hip"[MeSH Terms] OR "joints"[MeSH Terms])
