# Supplementary material for: Incidence and factors associated with postoperative delirium after primary total joint arthroplasty in older adults: a systematic review and meta-analysis
Source: Front Med (Lausanne). 2025 Oct 22;12:1664605. doi: 10.3389/fmed.2025.1664605 (PMC12586022; doi:10.3389/fmed.2025.1664605)
Supplement: Supplementary file 2 [file Data_Sheet_1.docx]

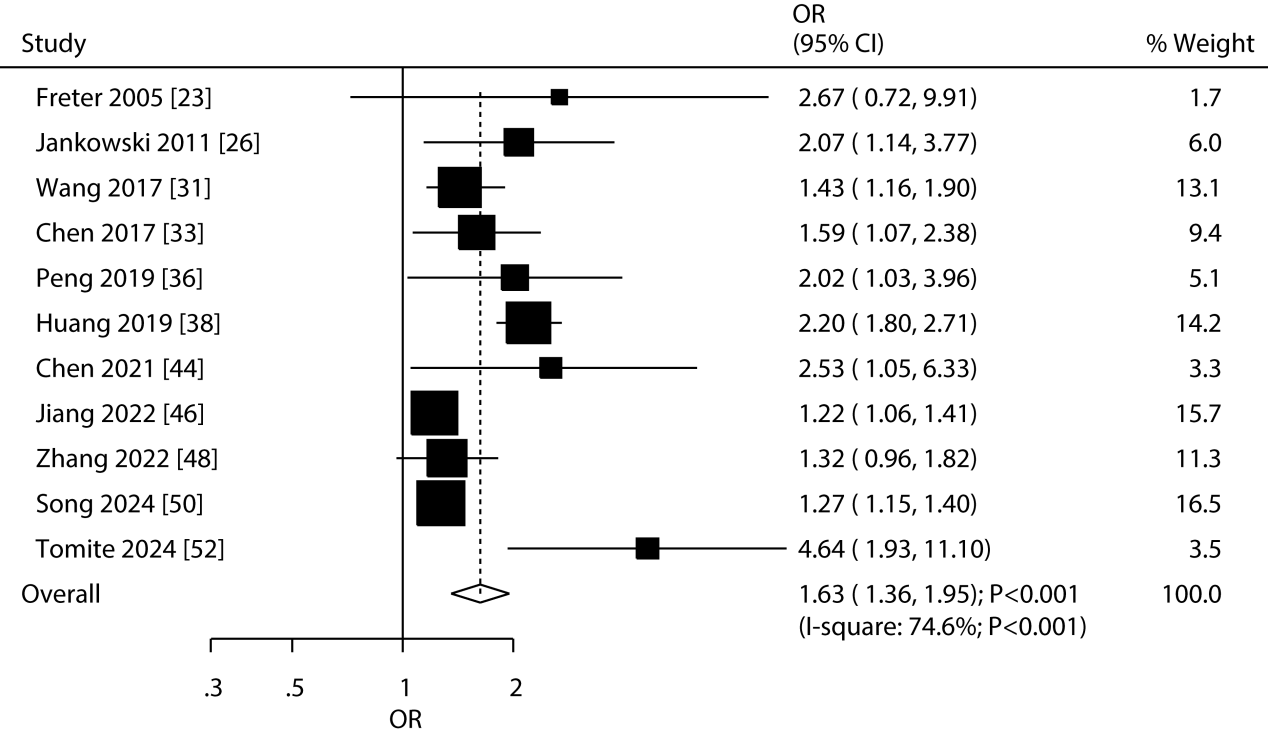


Figure S1. Association of age (Elderly vs younger) with the risk of POD


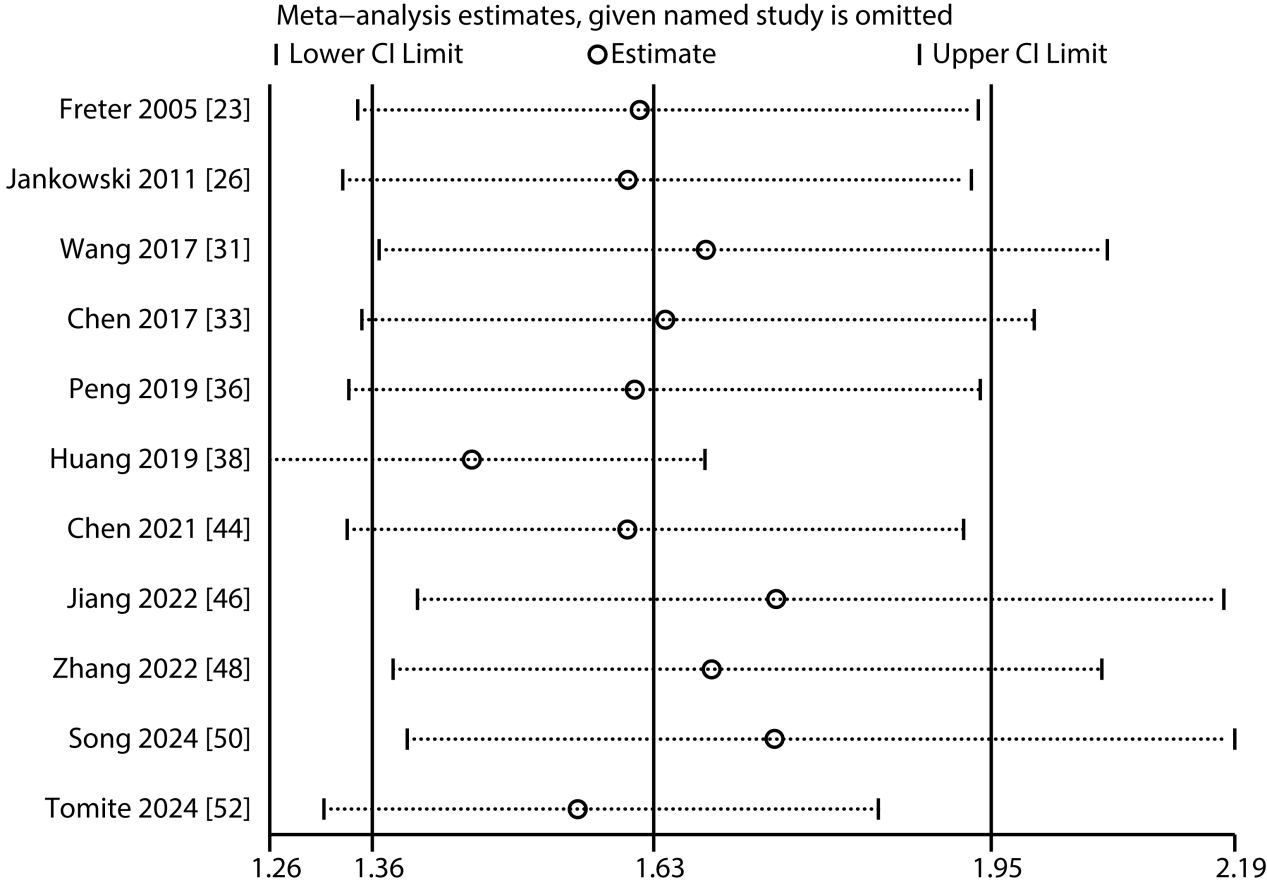


Figure S2. Sensitivity analysis regarding the association of age (Elderly vs younger) with the risk of POD


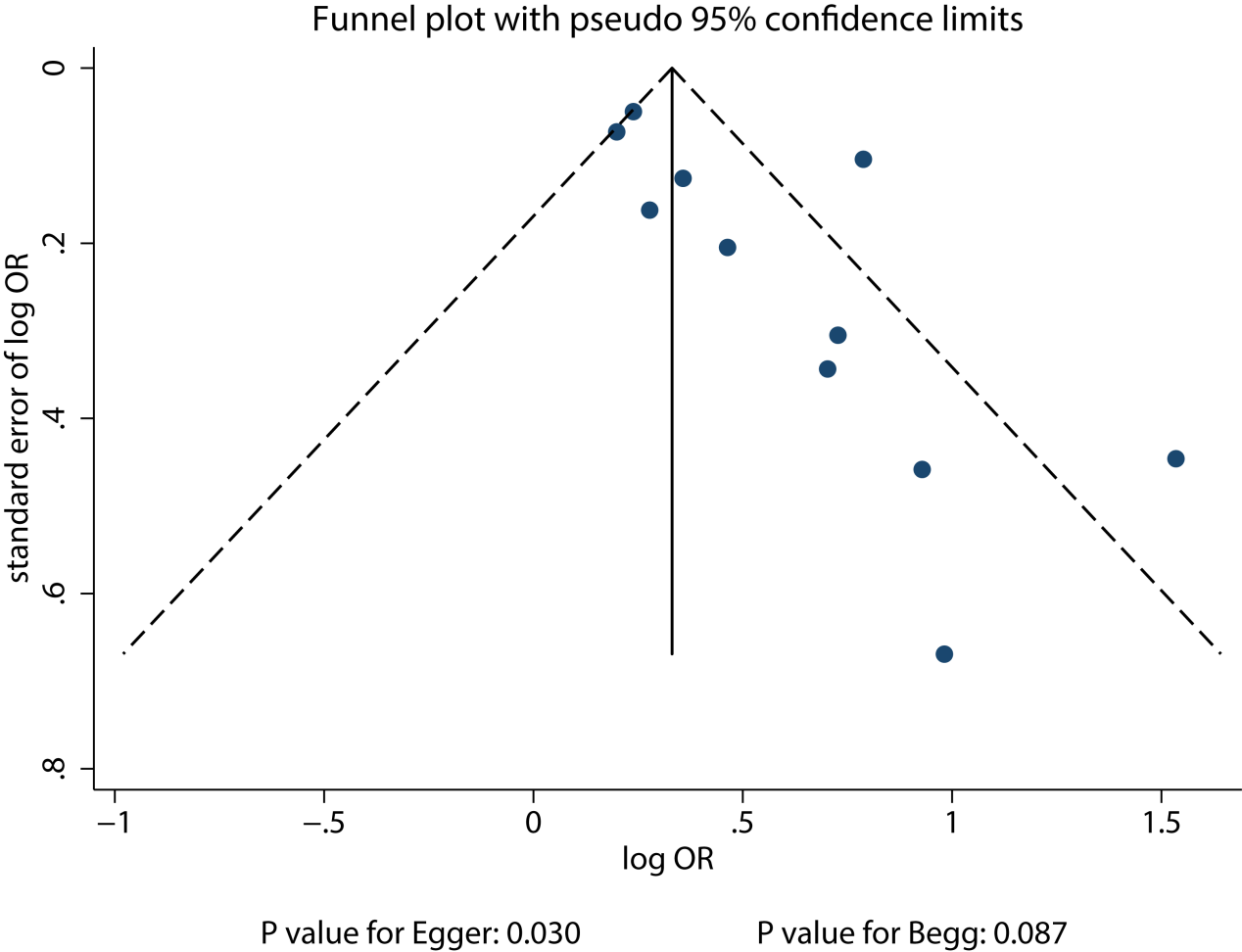


Figure S3. Funnel plot regarding the association of age (Elderly vs younger) with the risk of POD


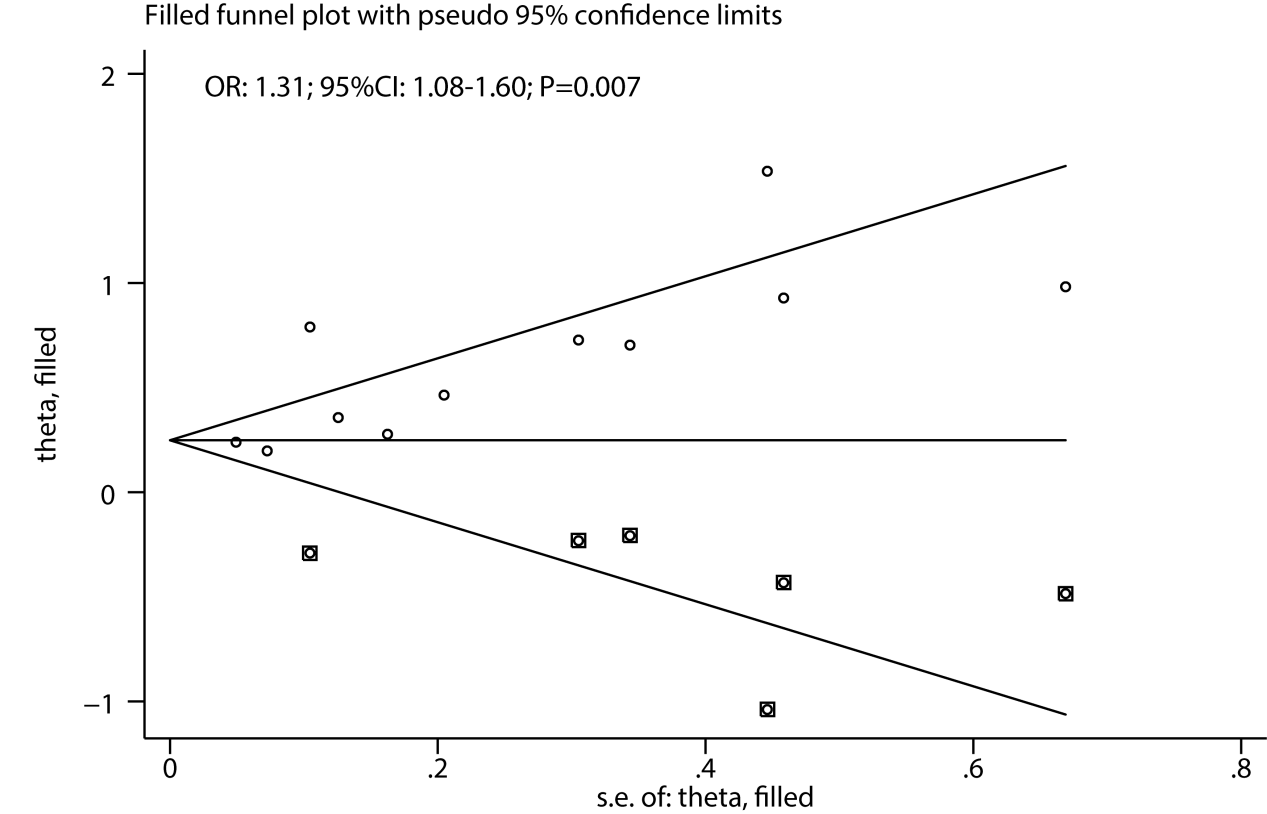


Figure S4. Trim and fill regarding the association of age (Elderly vs younger) with the risk of POD


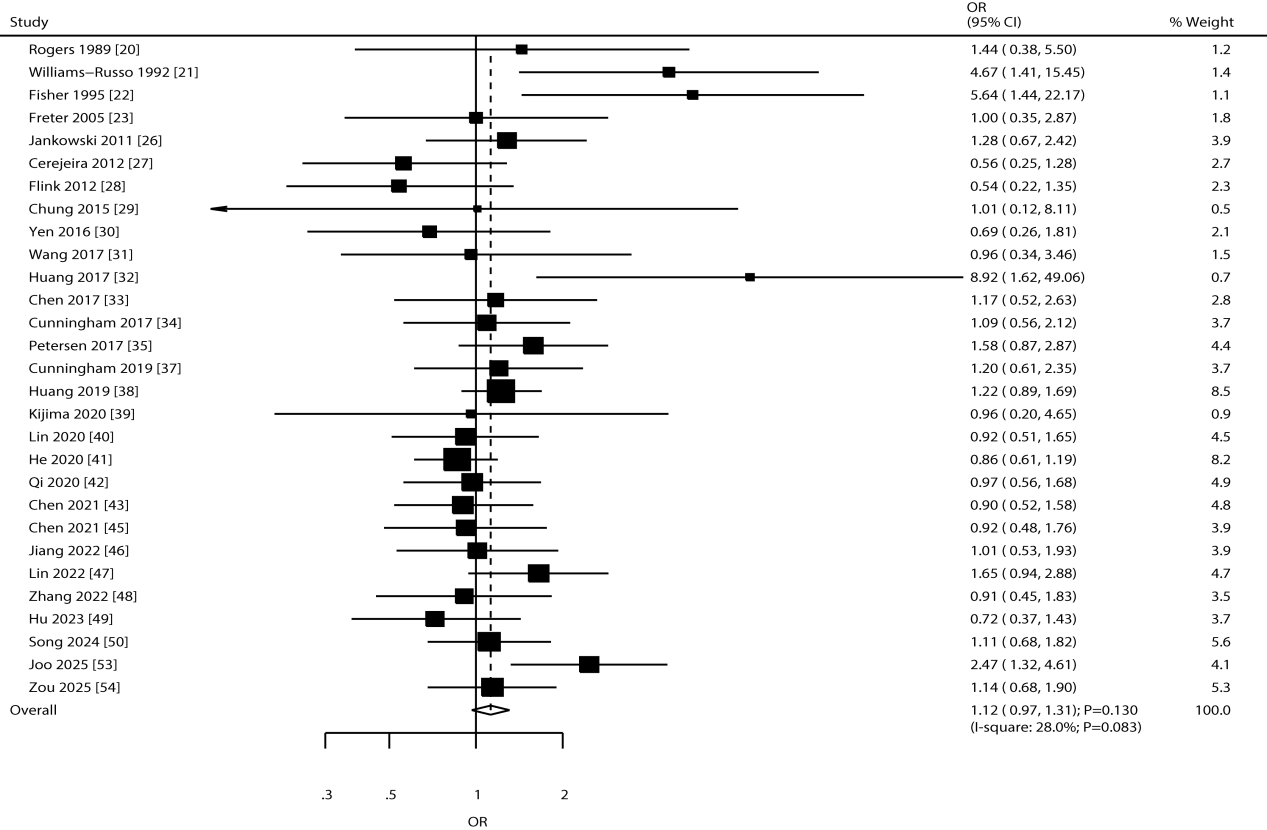


Figure S5. Association of sex (male vs female) with the risk of POD


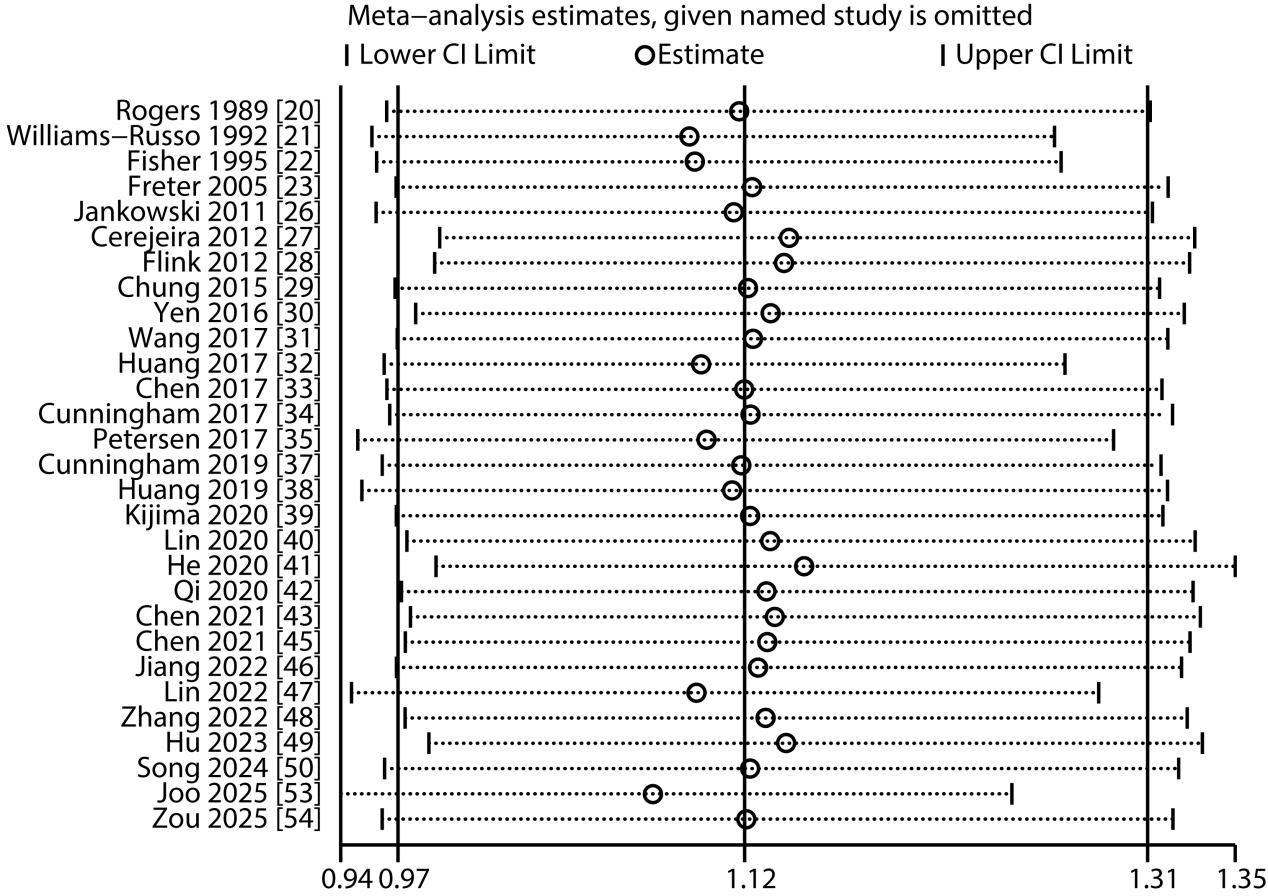


Figure S6. Sensitivity analysis regarding the association of sex (male vs female) with the risk of POD


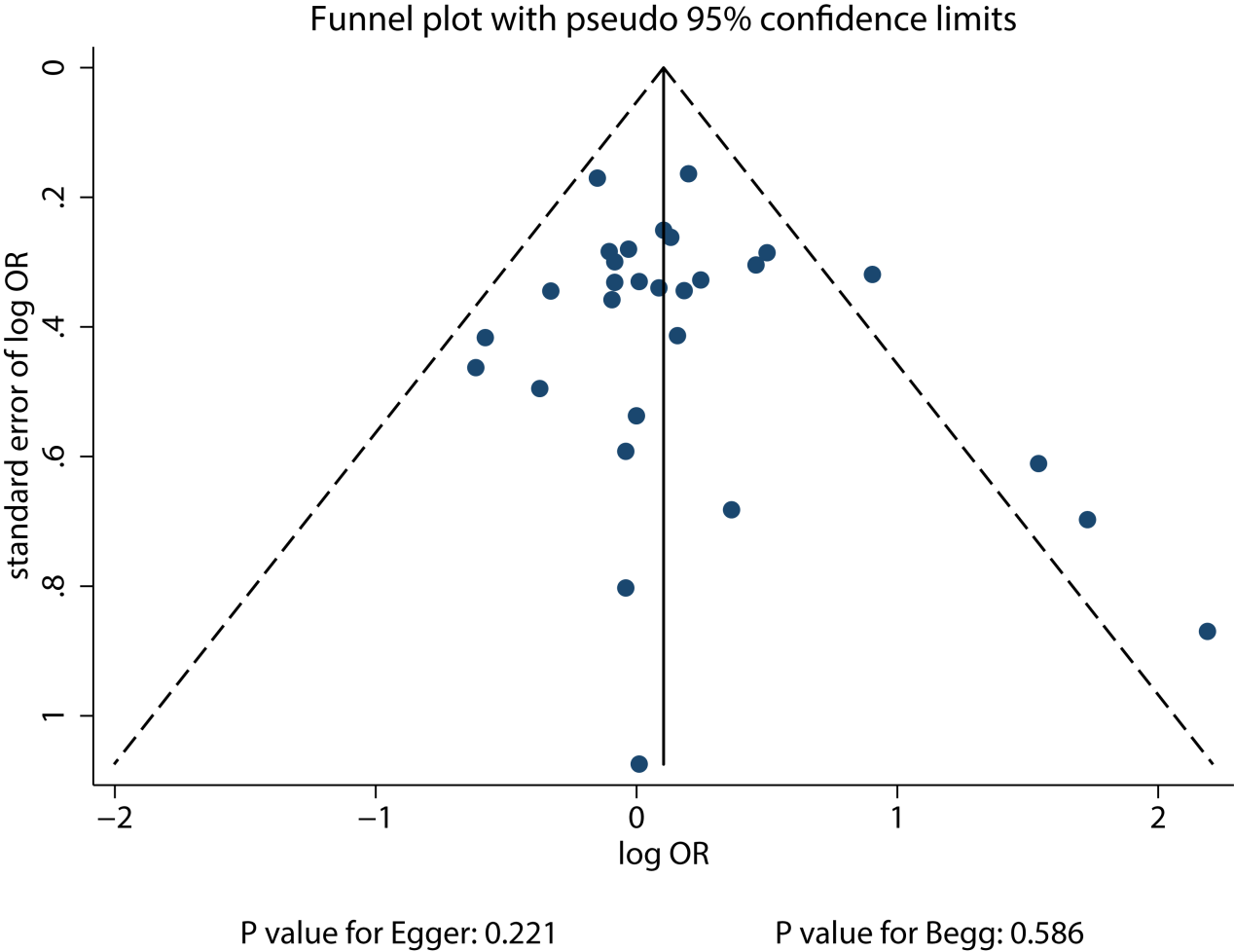


Figure S7. Funnel plot regarding the association of sex (male vs female) with the risk of POD


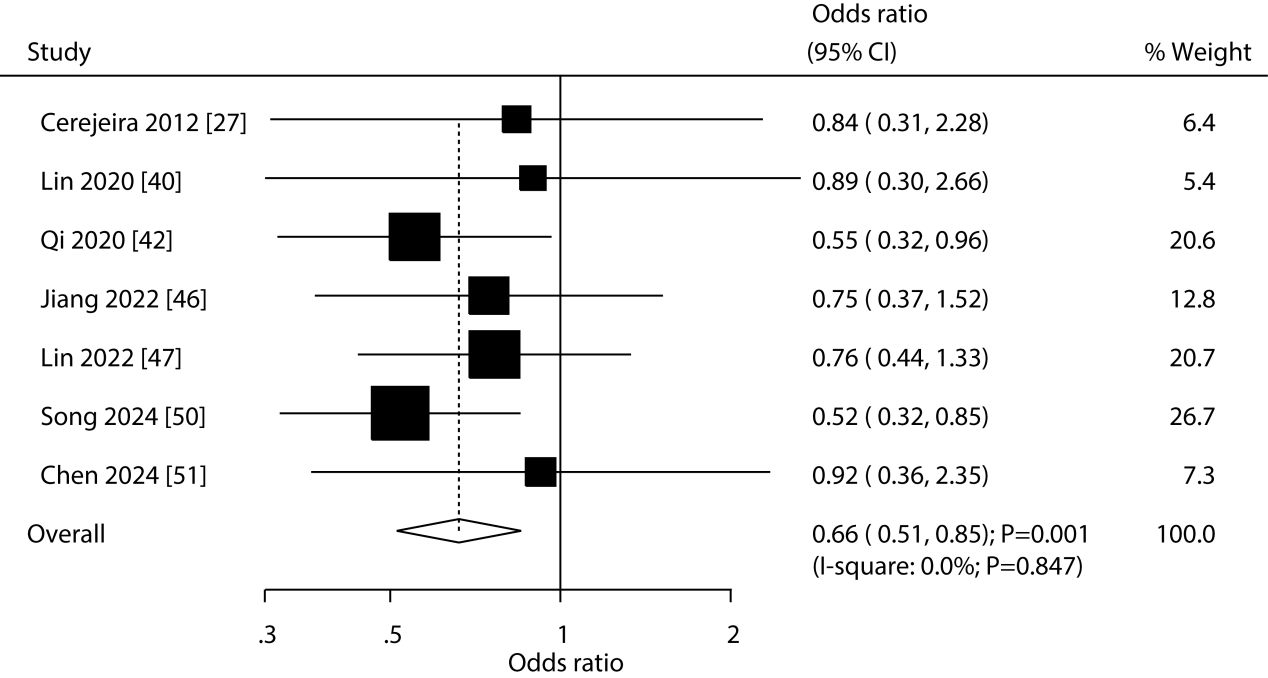


Figure S8. Association of educational level (high vs low) with the risk of POD


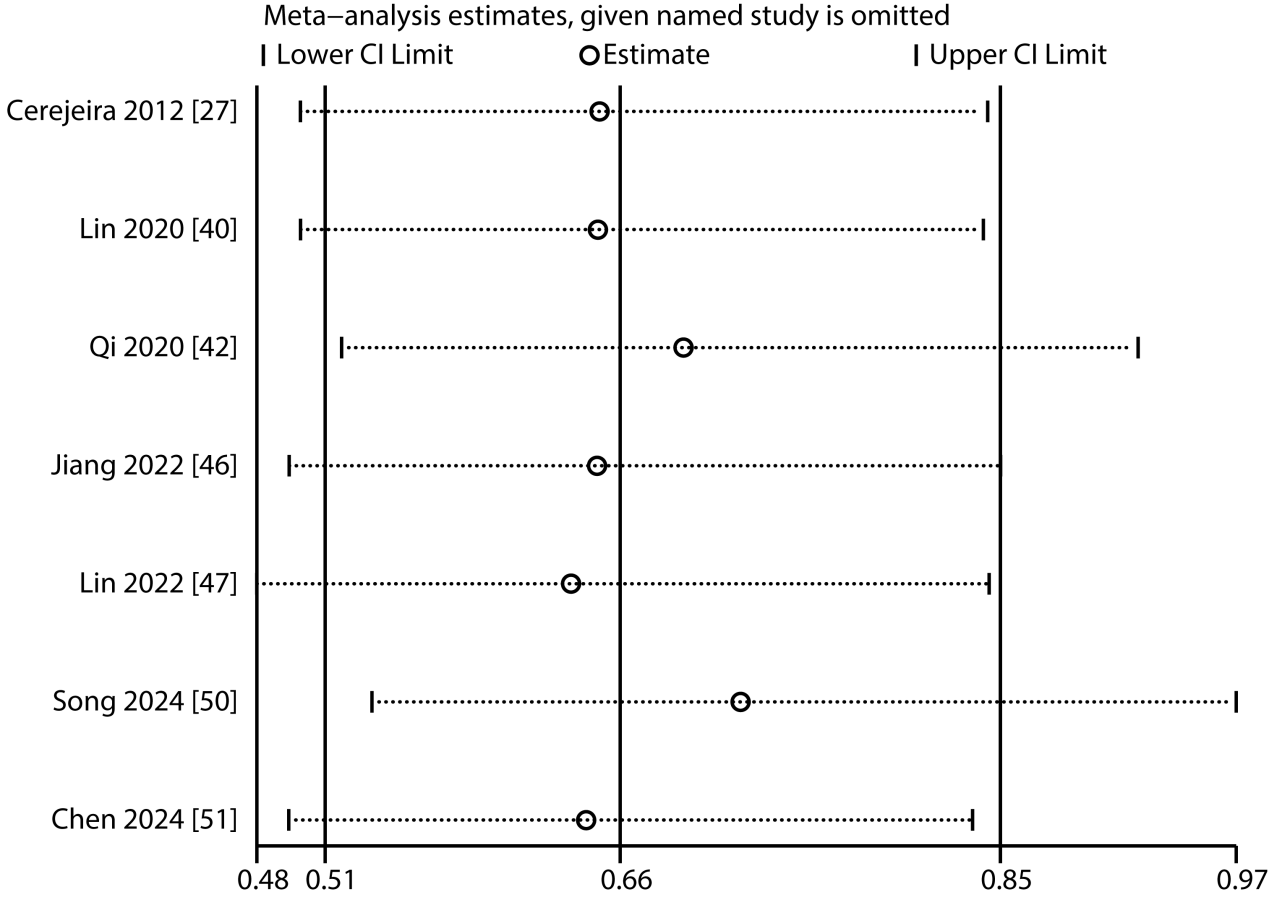


Figure S9. Sensitivity analysis regarding the association of educational level (high vs low) with the risk of POD


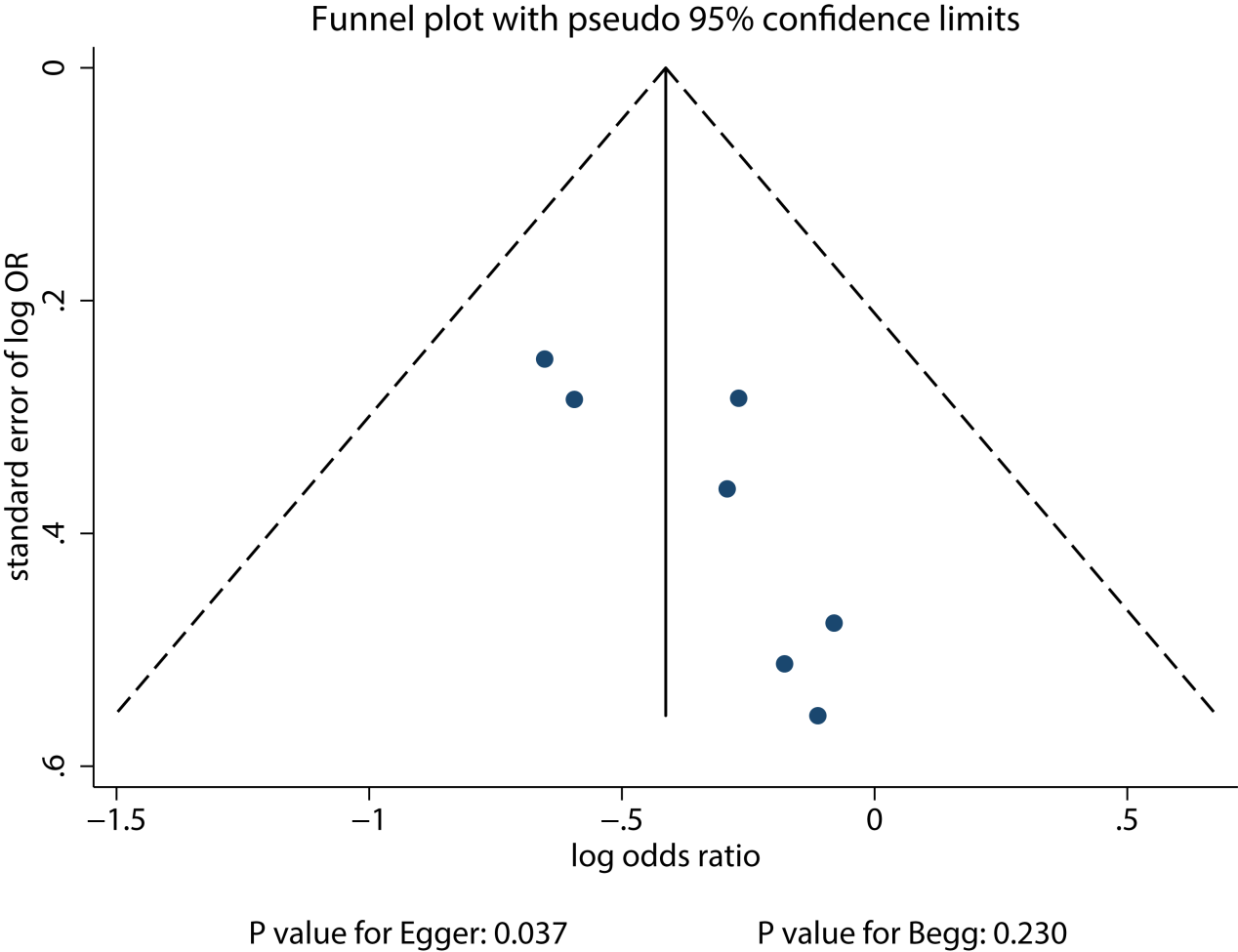


Figure S10. Funnel plot regarding the association of educational level (high vs low) with the risk of POD


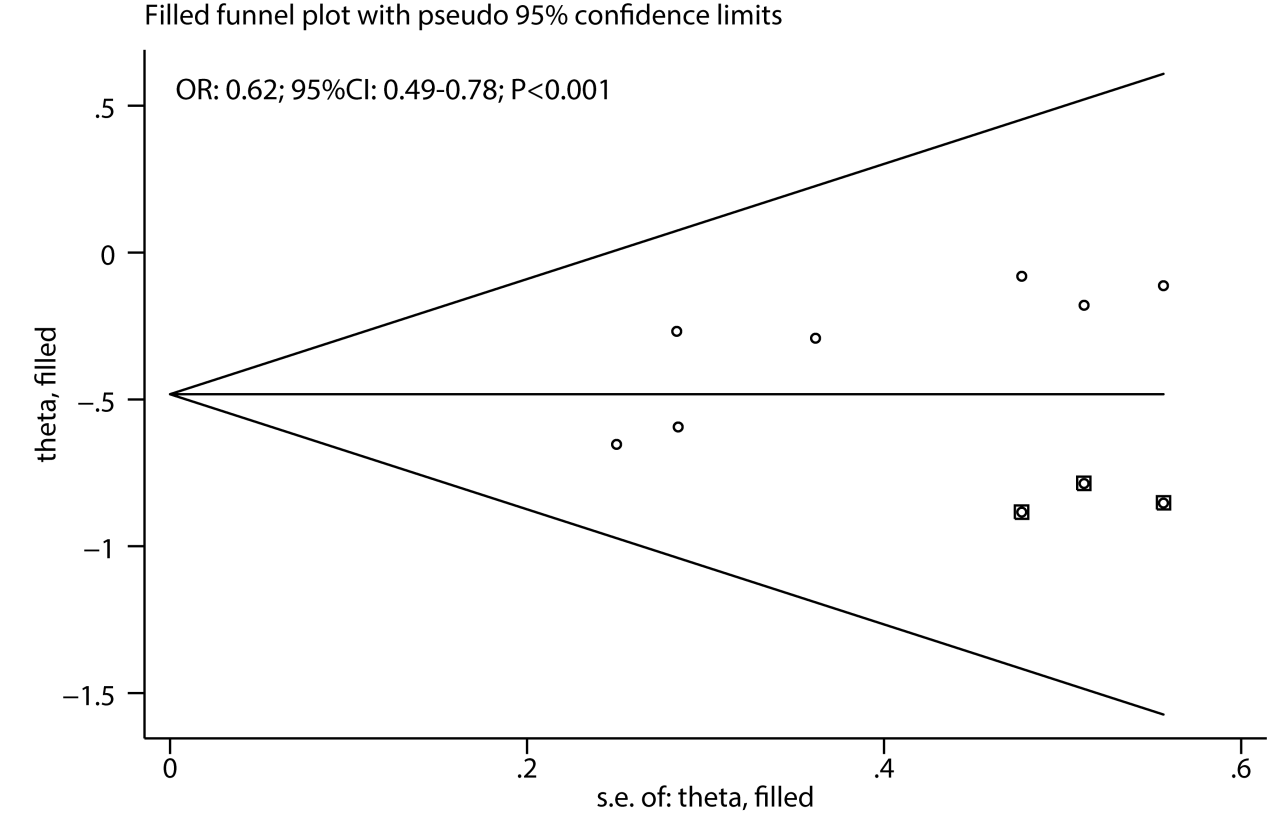


Figure S11. Trim and fill regarding the association of educational level (high vs low) with the risk of POD


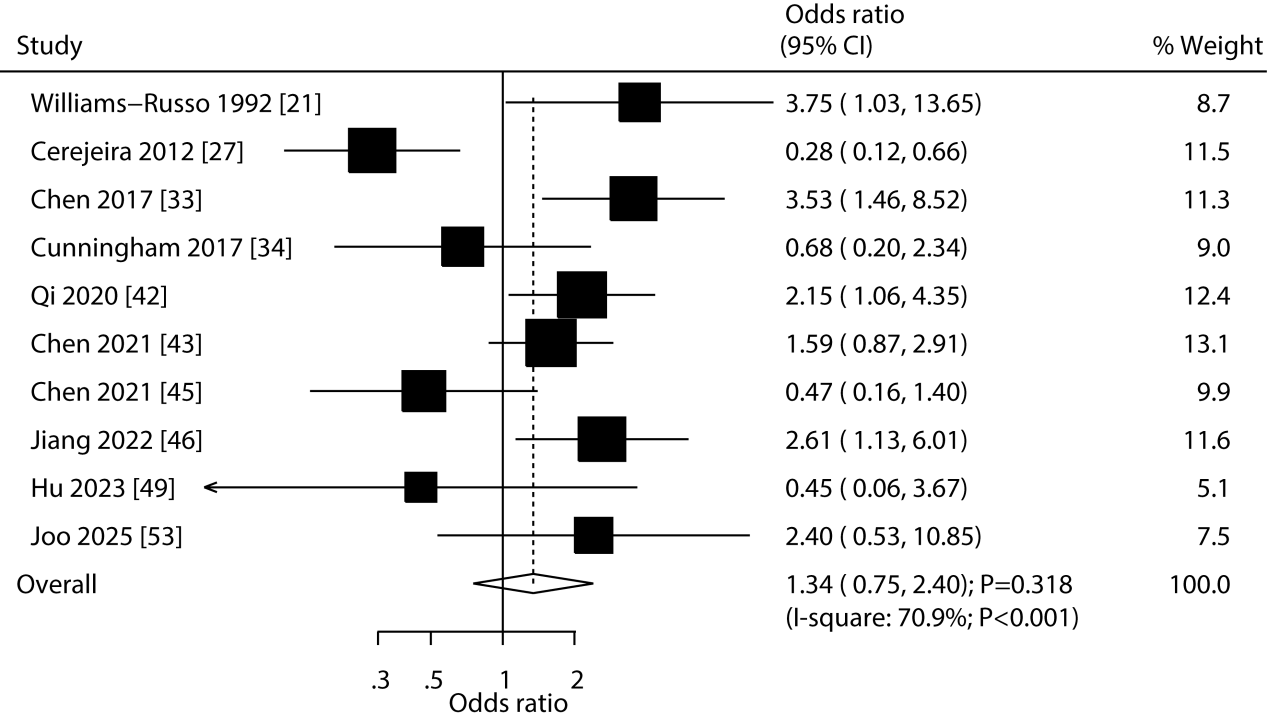


Figure S12. Association of alcohol use with the risk of POD


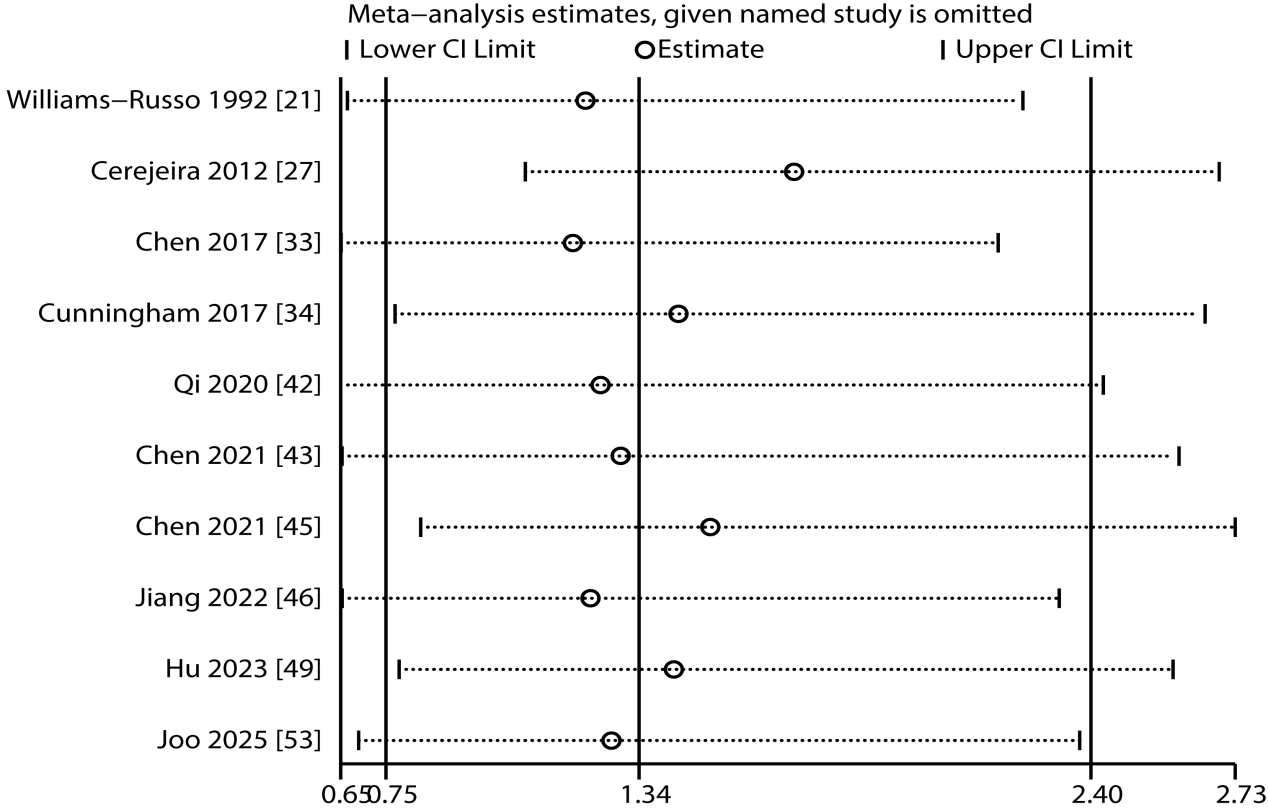


Figure S13. Sensitivity analysis regarding the association of alcohol use with the risk of POD


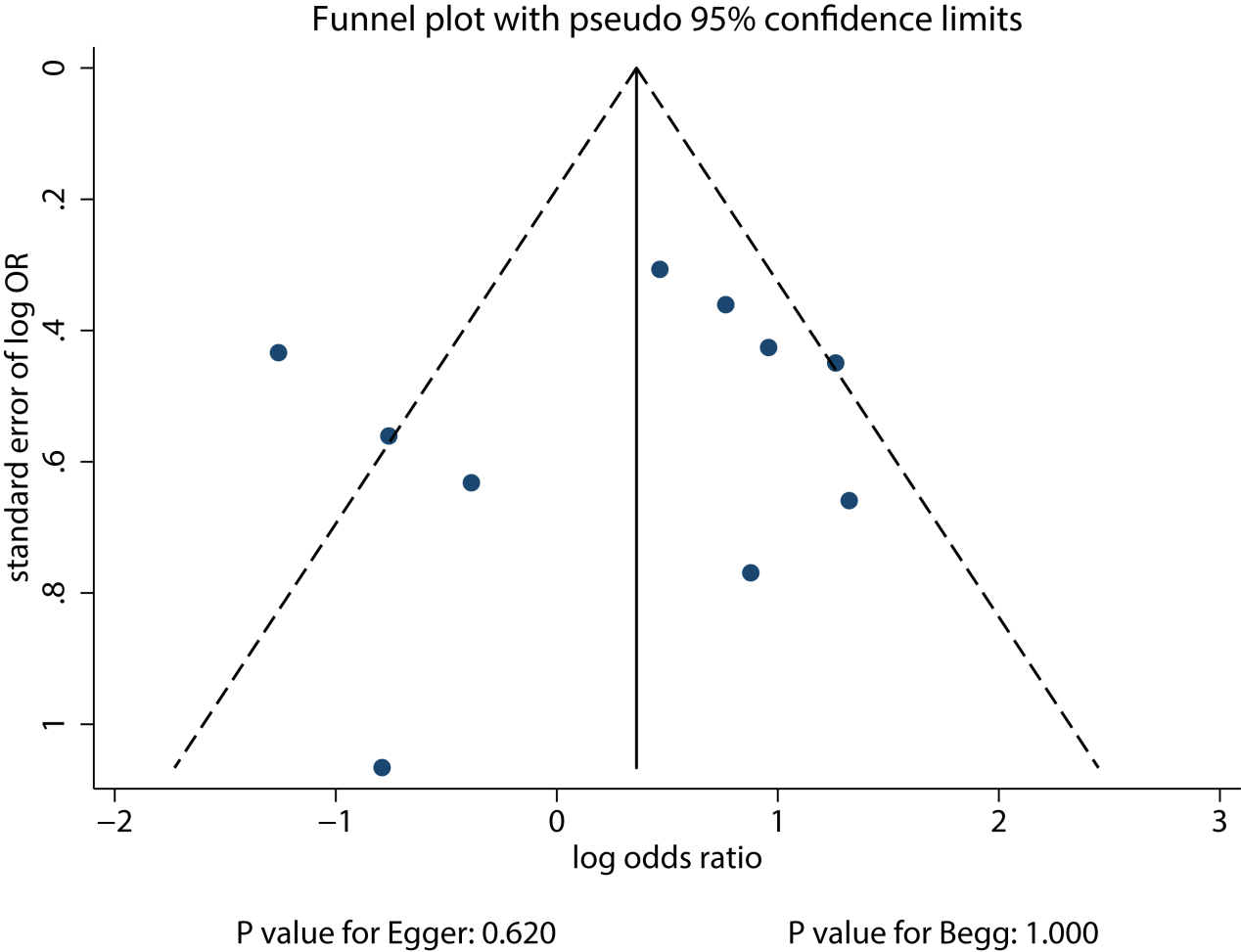


Figure S14. Funnel plot regarding the association of alcohol use with the risk of POD


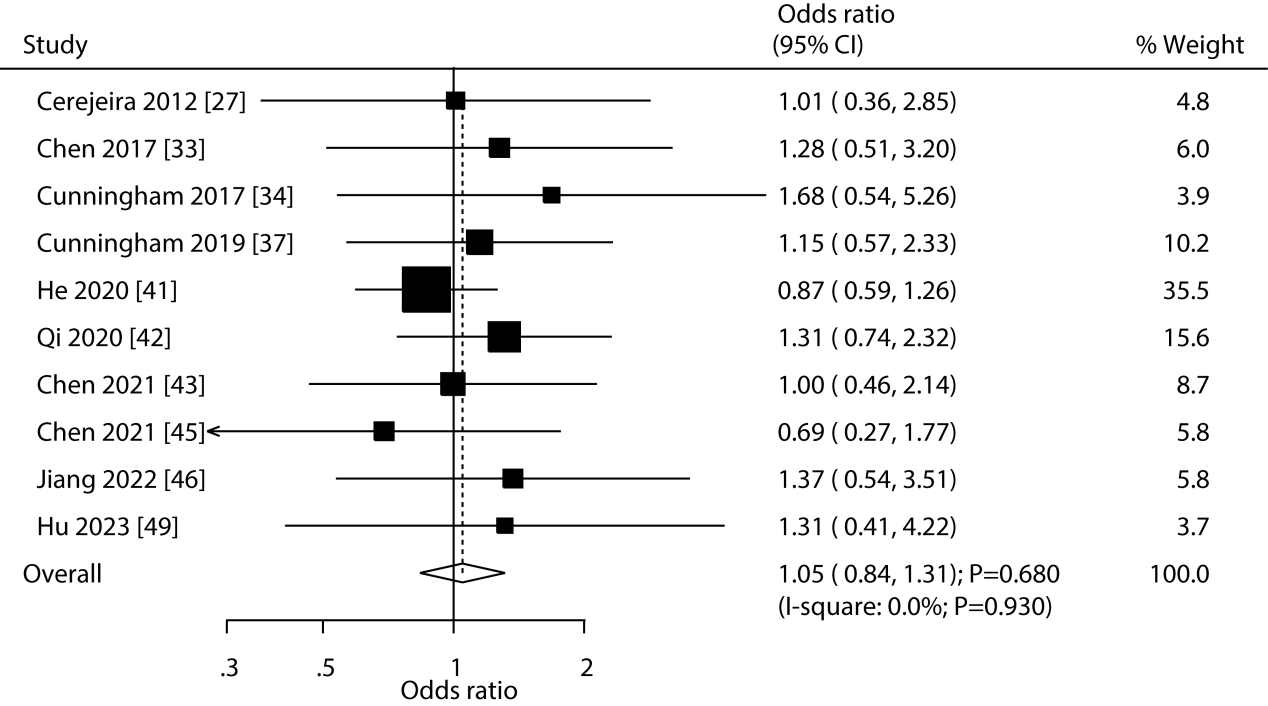


Figure S15. Association of smoking with the risk of POD


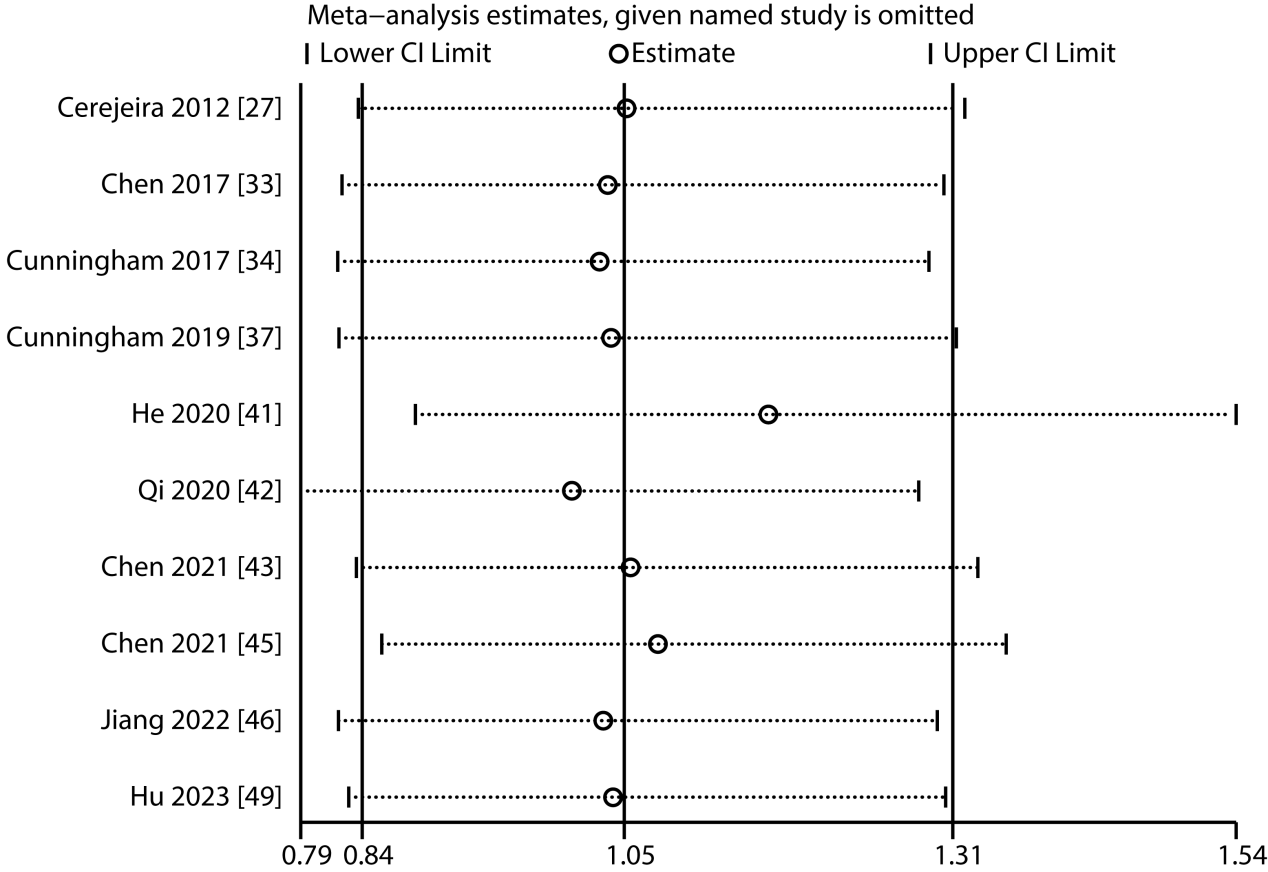


Figure S16. Sensitivity analysis regarding association of smoking with the risk of POD


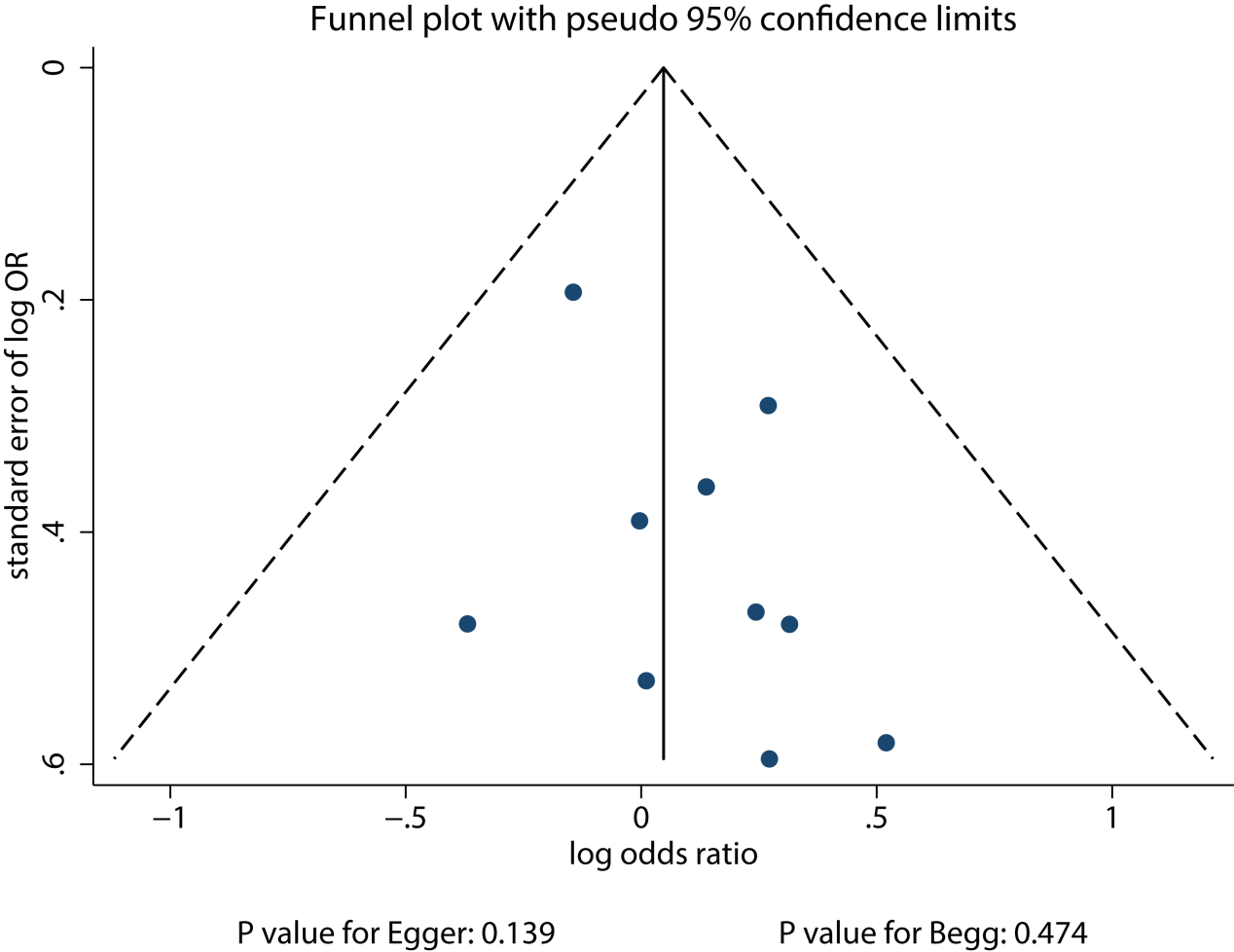


Figure S17. Funnel plot regarding association of smoking with the risk of POD


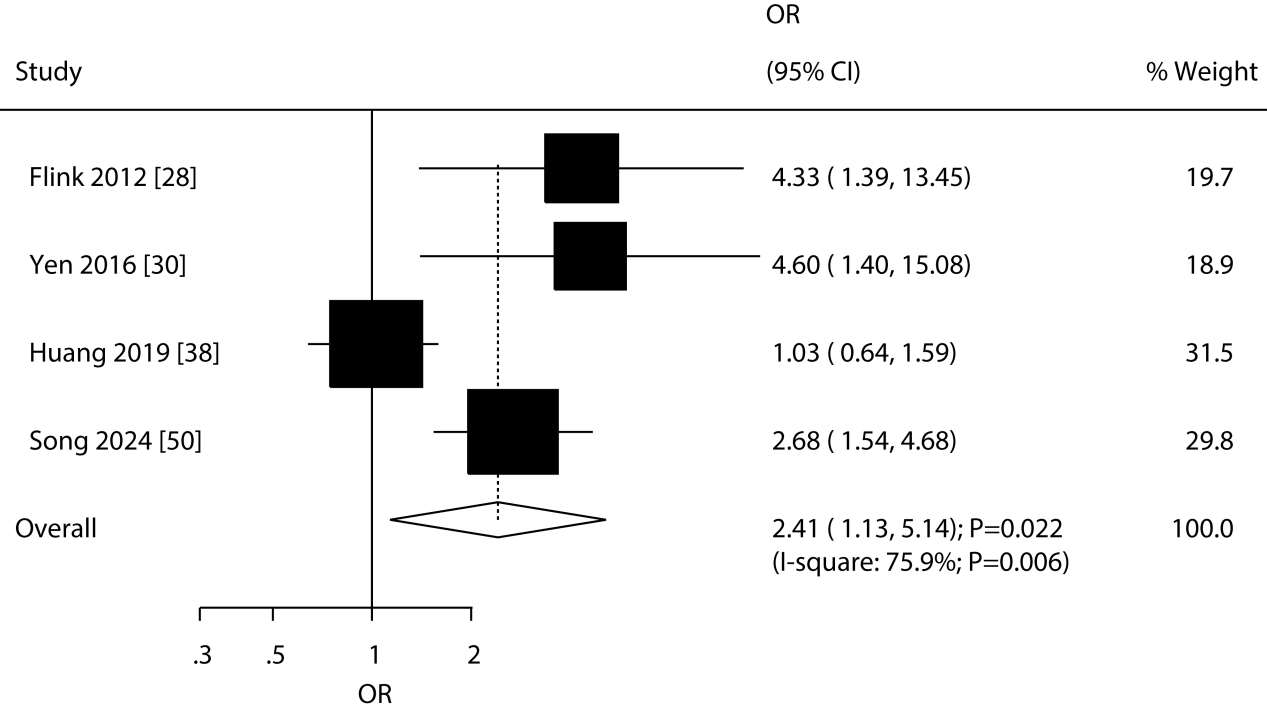


Figure S18. Association of sleep apnea with the risk of POD


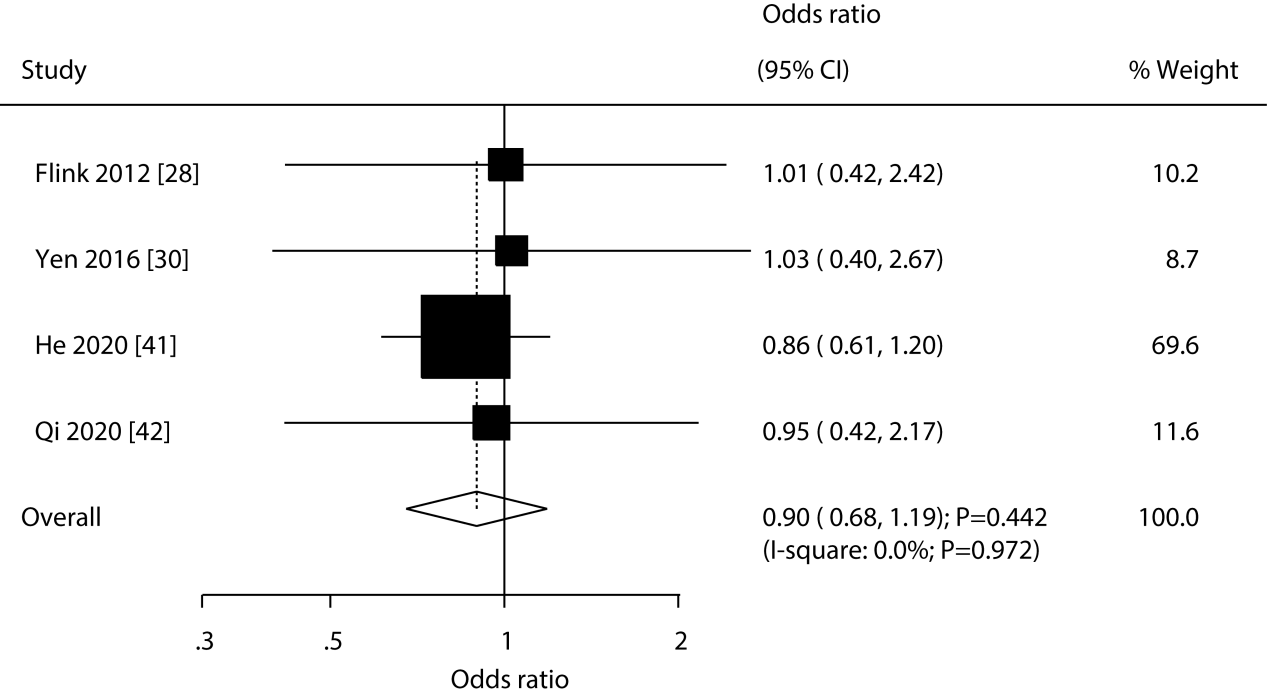


Figure S19. Association of hyperlipidemia with the risk of POD


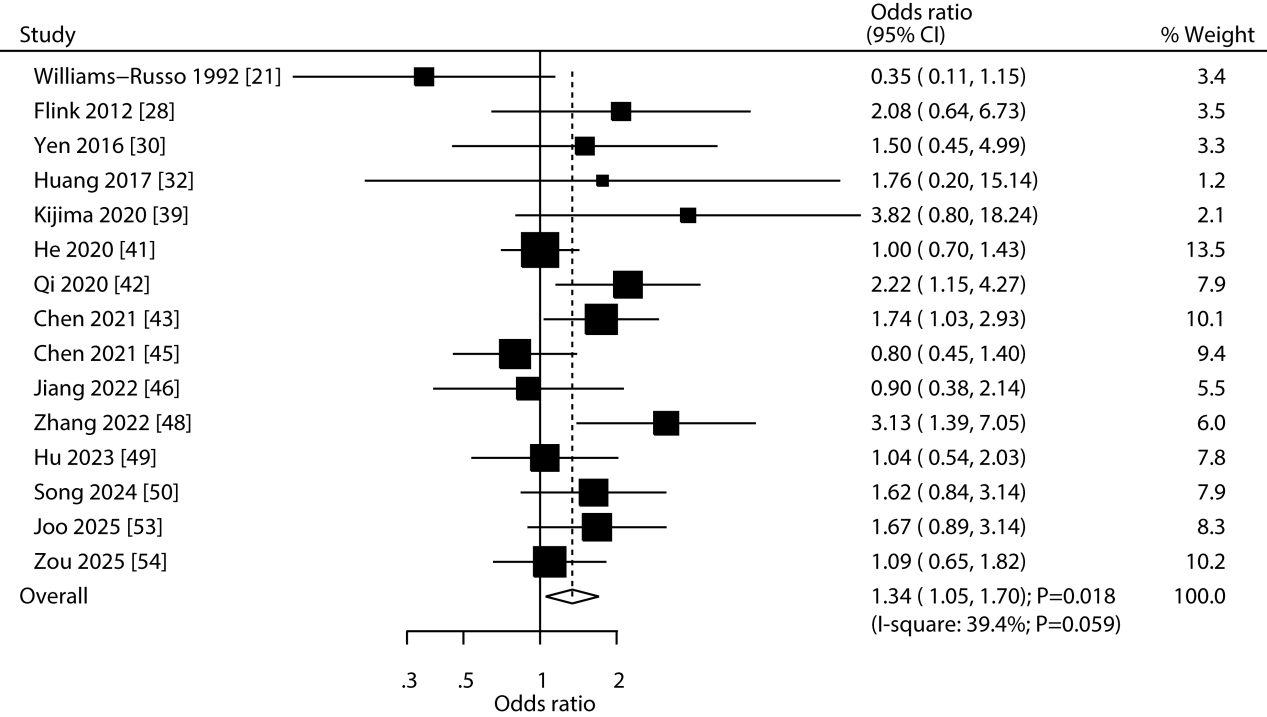


Figure S20. Association of hypertension with the risk of POD


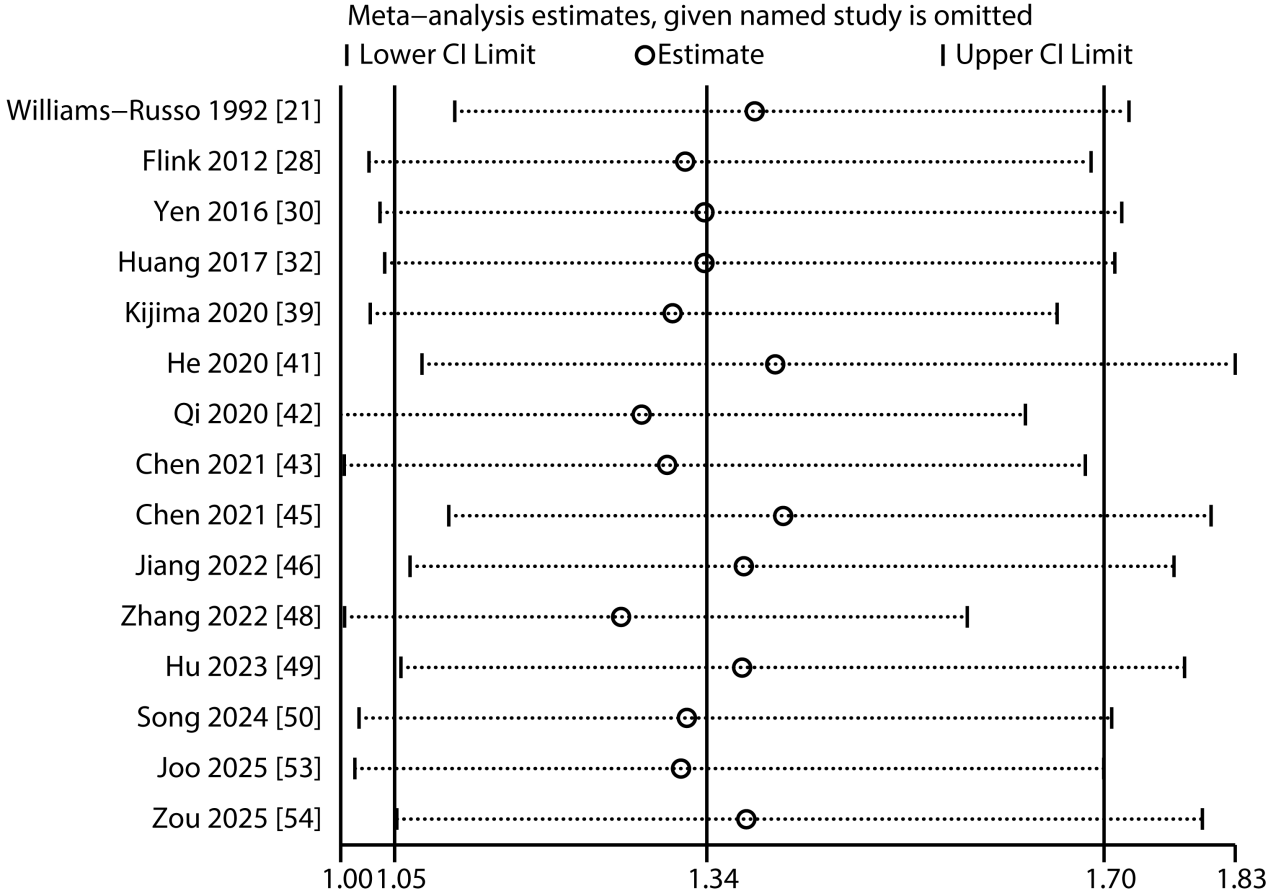


Figure S21. Sensitivity analysis regarding the association of hypertension with the risk of POD


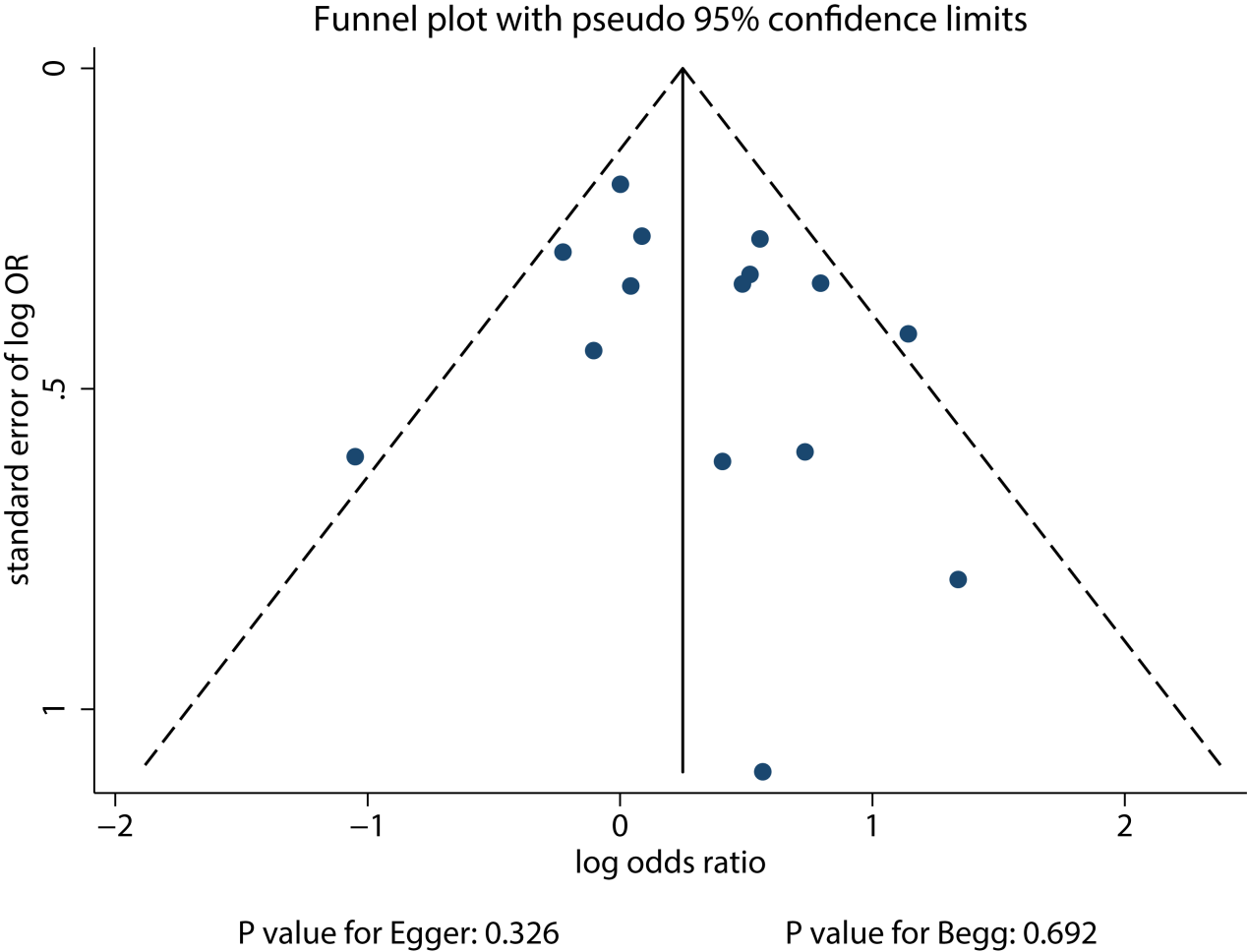


Figure S22. Funnel plot regarding the association of hypertension with the risk of POD


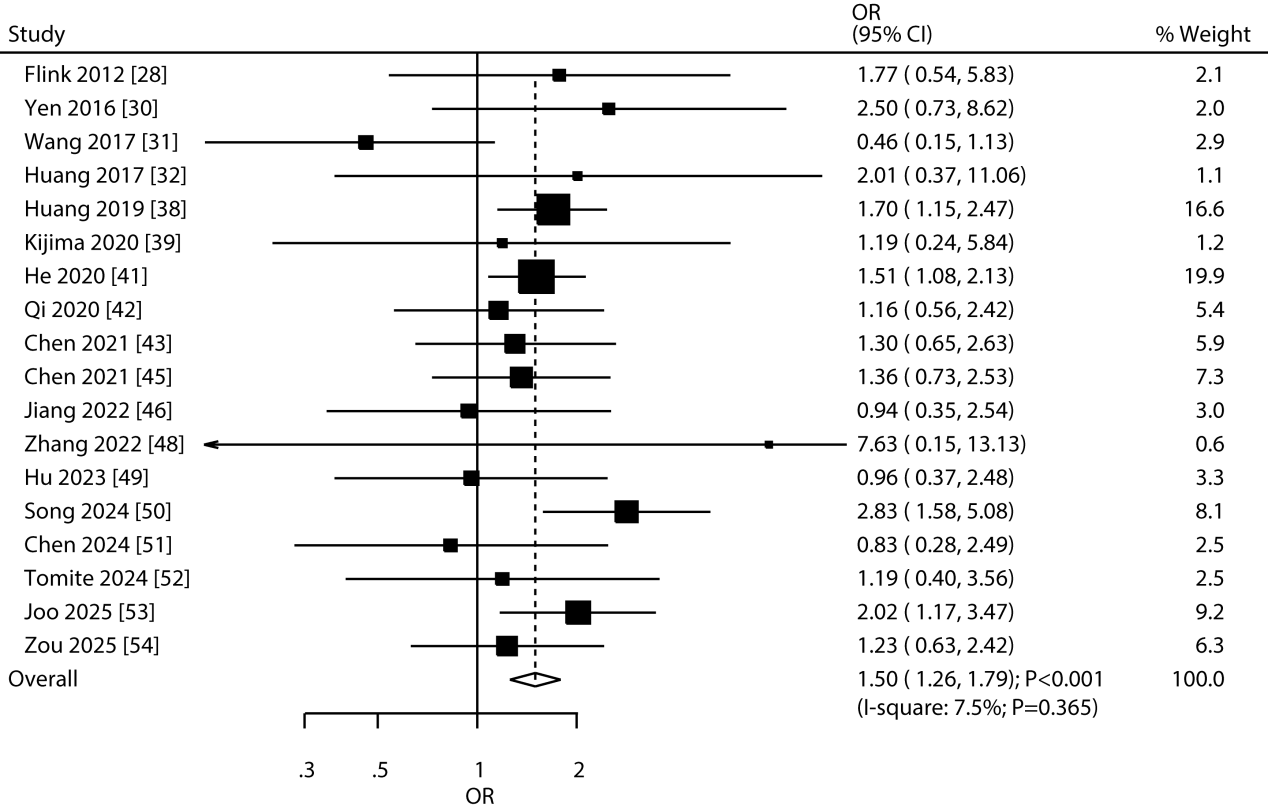


Figure S23. Association of DM with the risk of POD


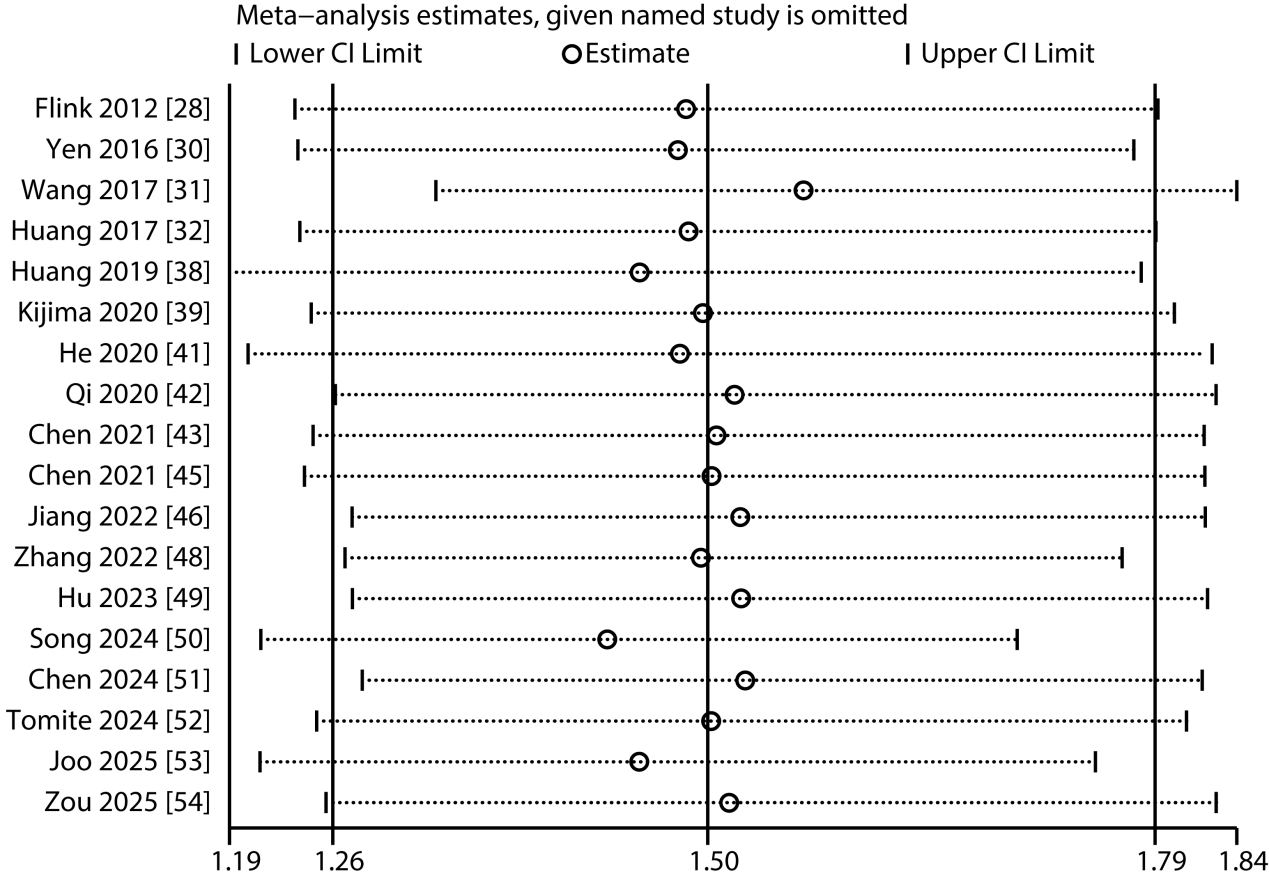


Figure S24. Sensitivity analysis regarding the association of DM with the risk of POD


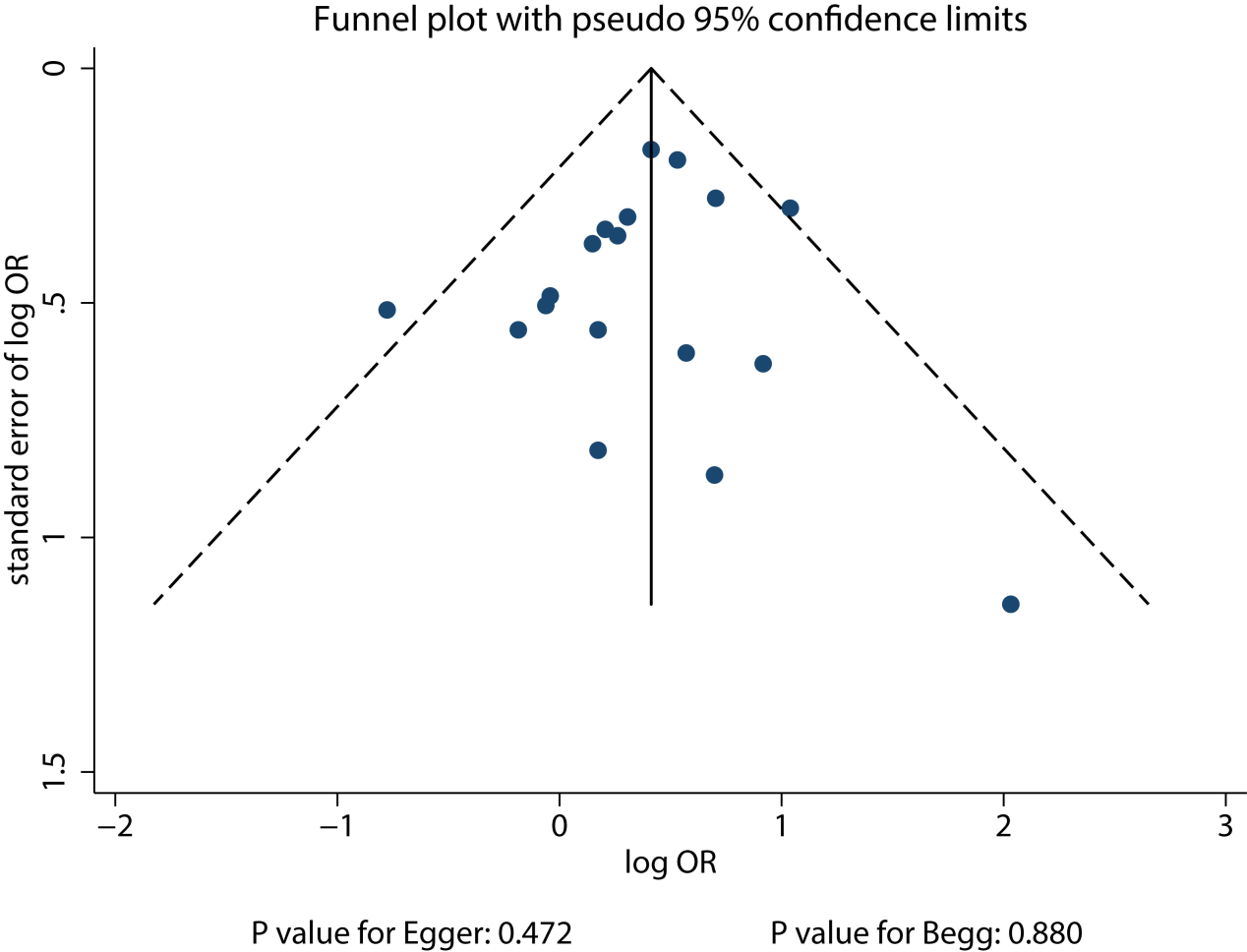


Figure S25. Funnel plot regarding the association of DM with the risk of POD


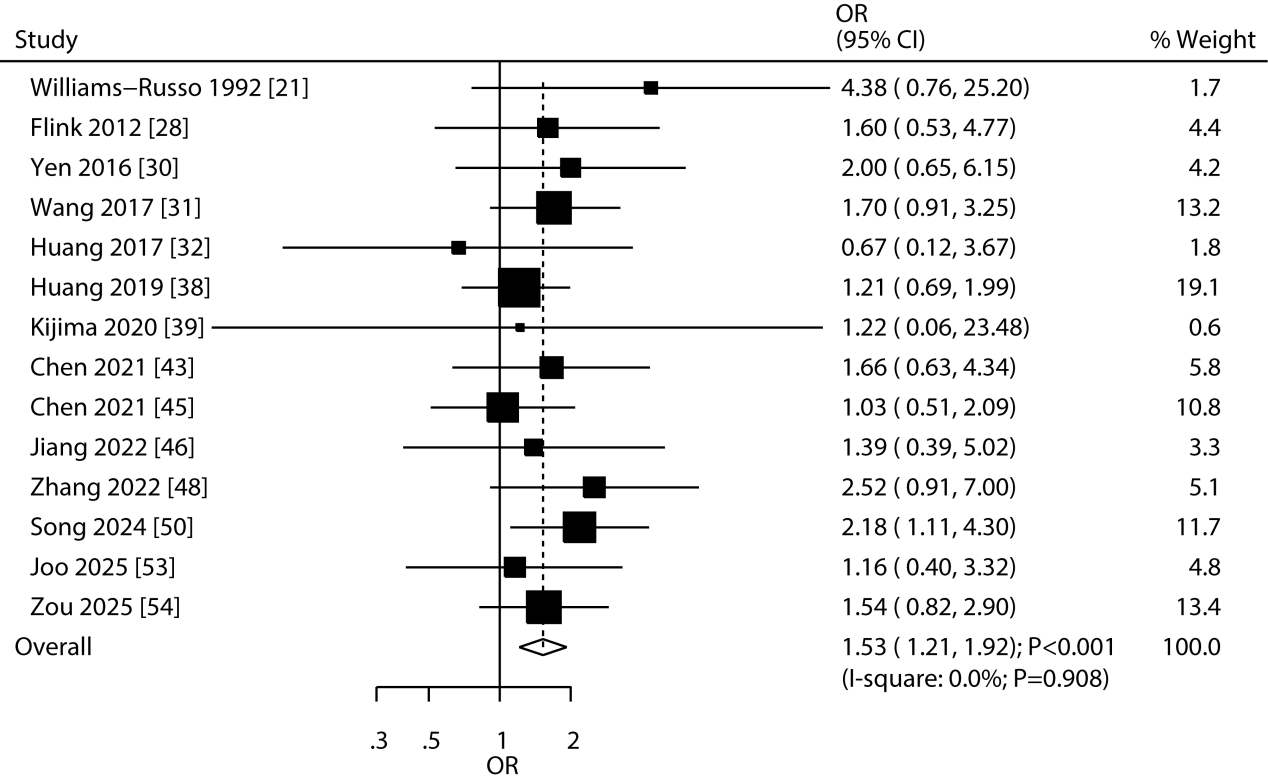


Figure S26. Association of CAD with the risk of POD


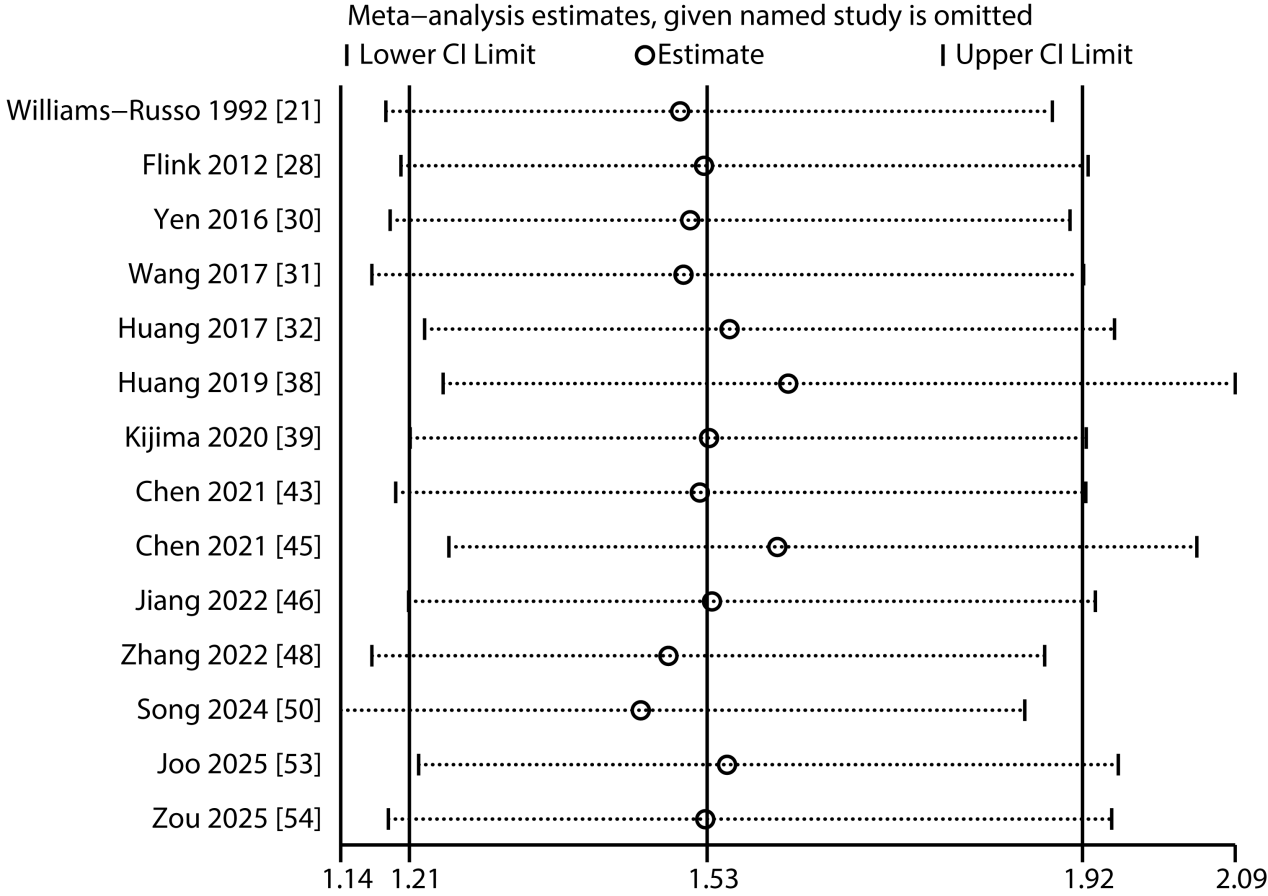


Figure S27. Sensitivity analysis regarding the association of CAD with the risk of POD


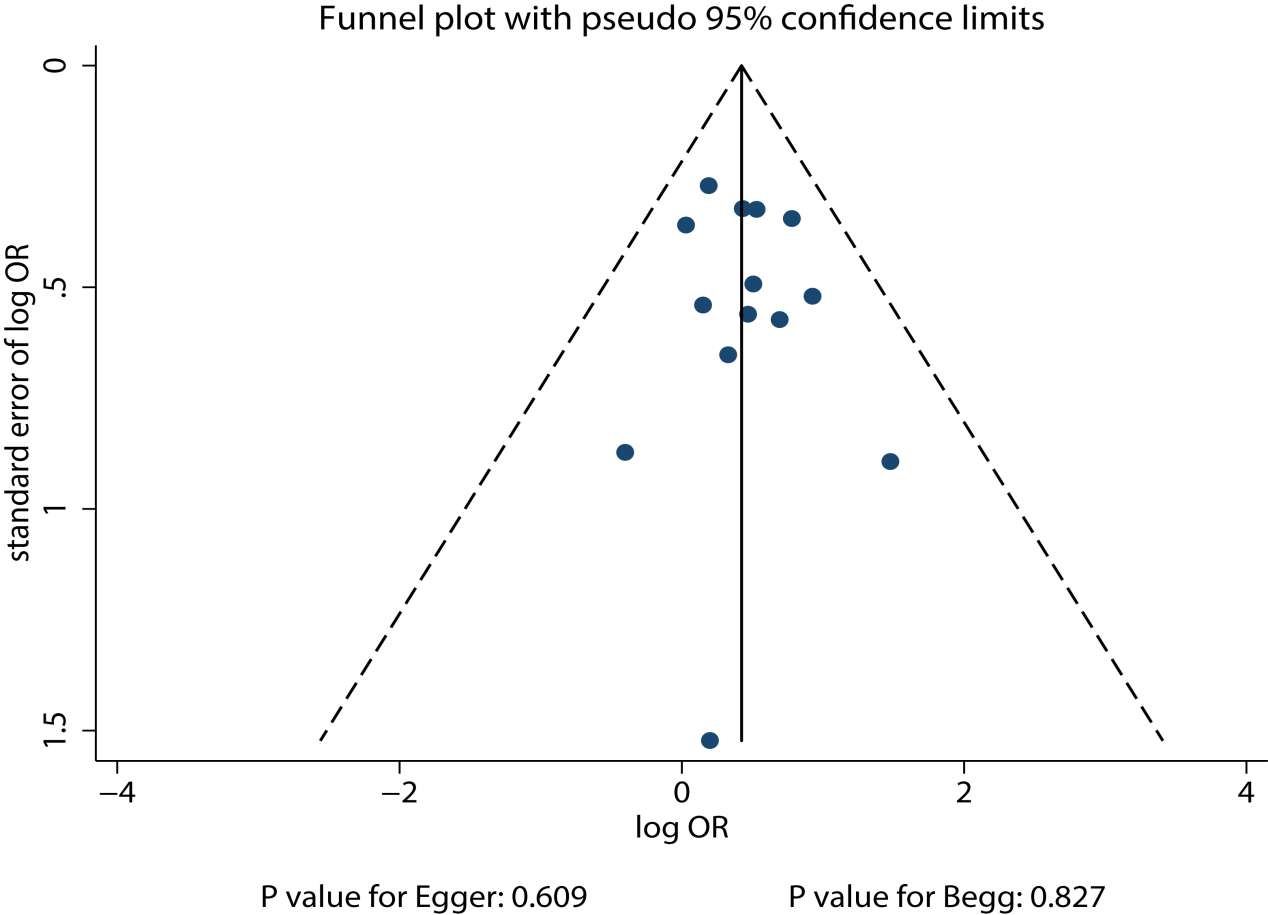


Figure S28. Funnel plot regarding the association of CAD with the risk of POD


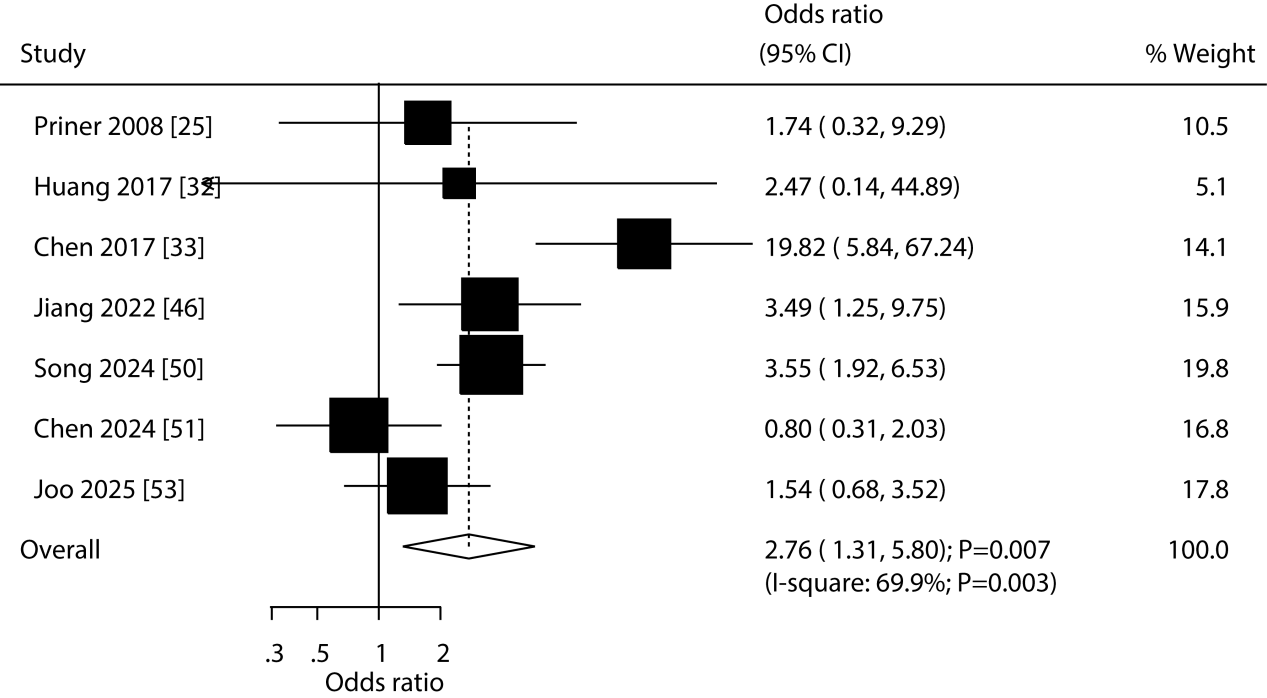


Figure S29. Association of stroke with the risk of POD


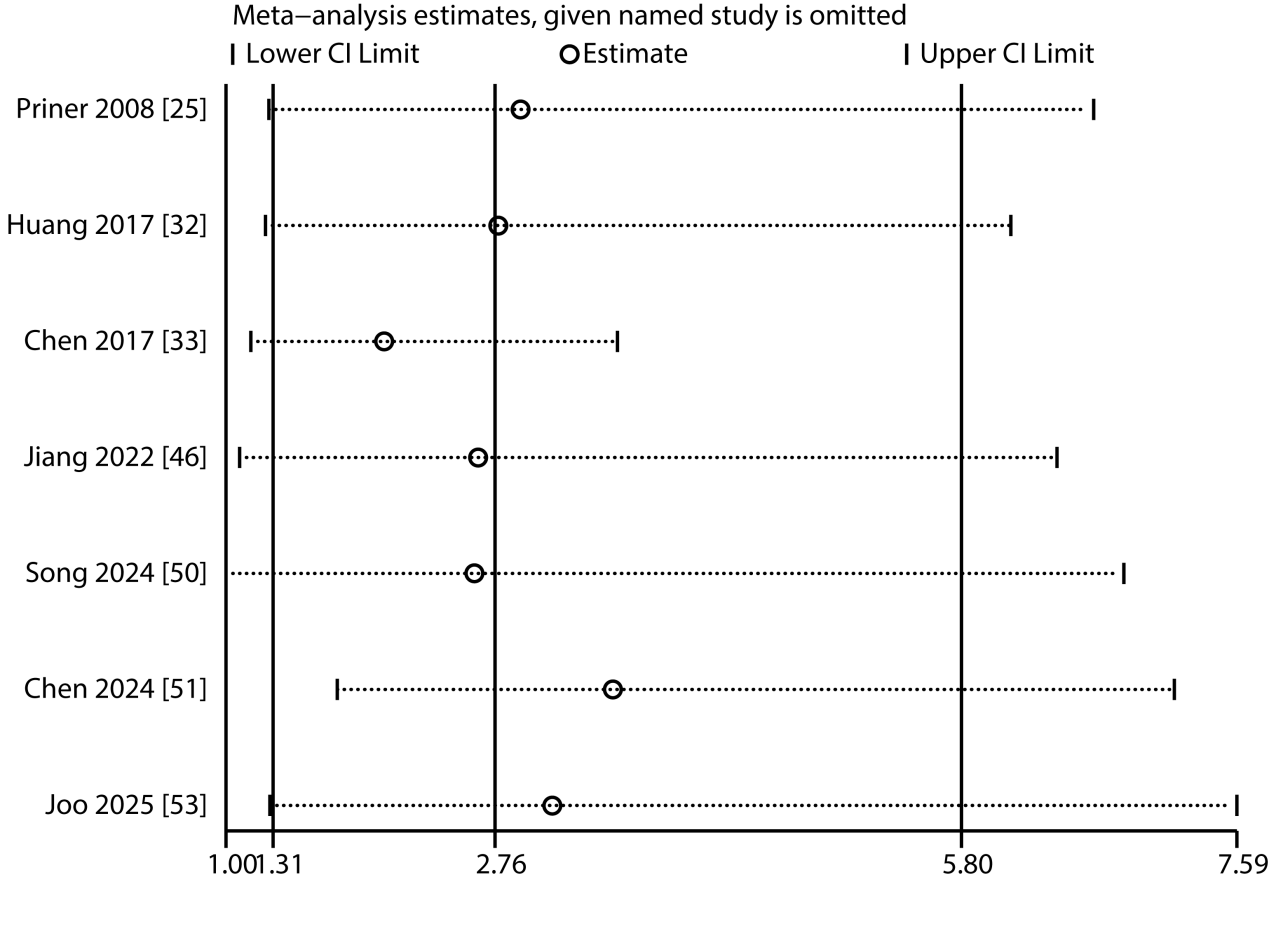


Figure S30. Sensitivity analysis regarding the association of stroke with the risk of POD


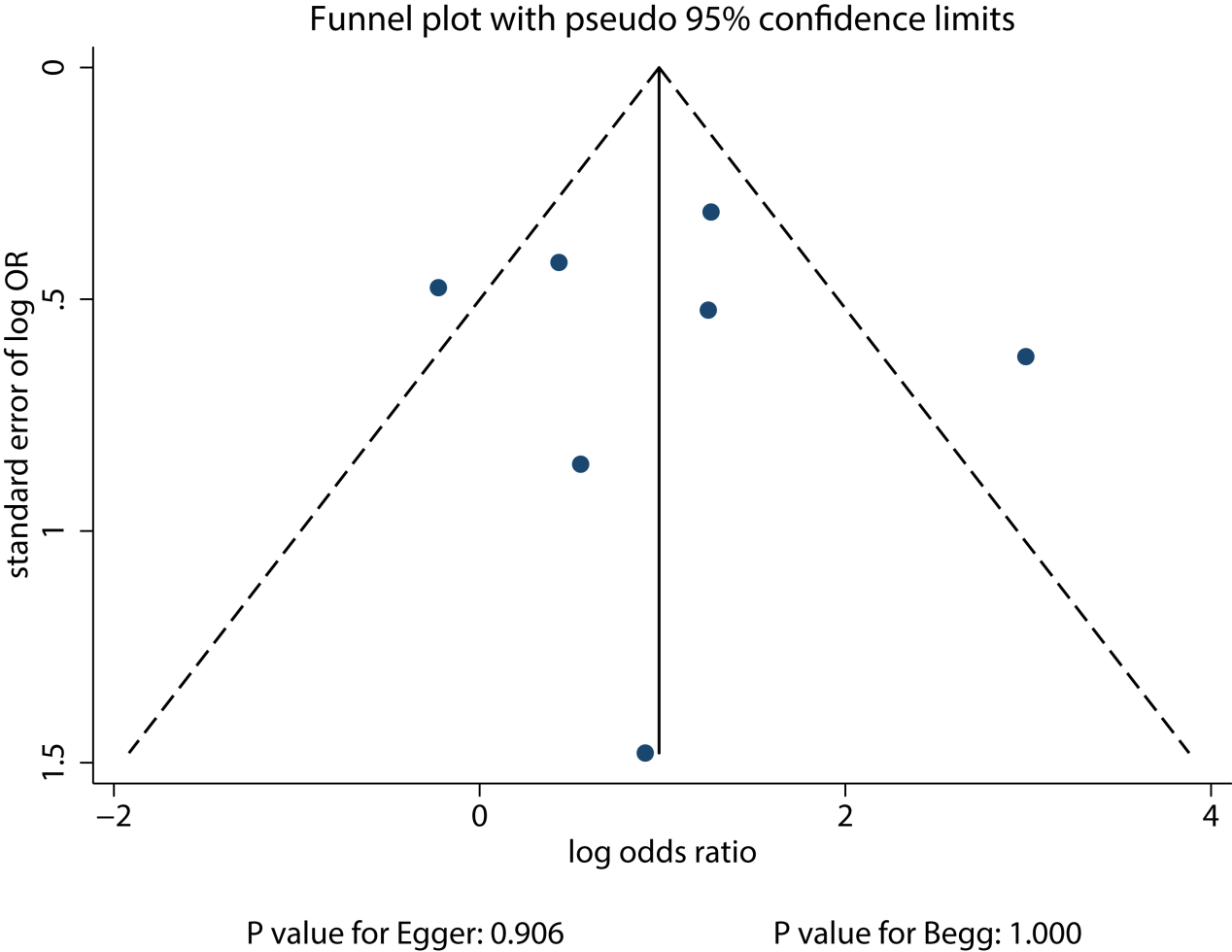


Figure S31. Funnel plot regarding the association of stroke with the risk of POD


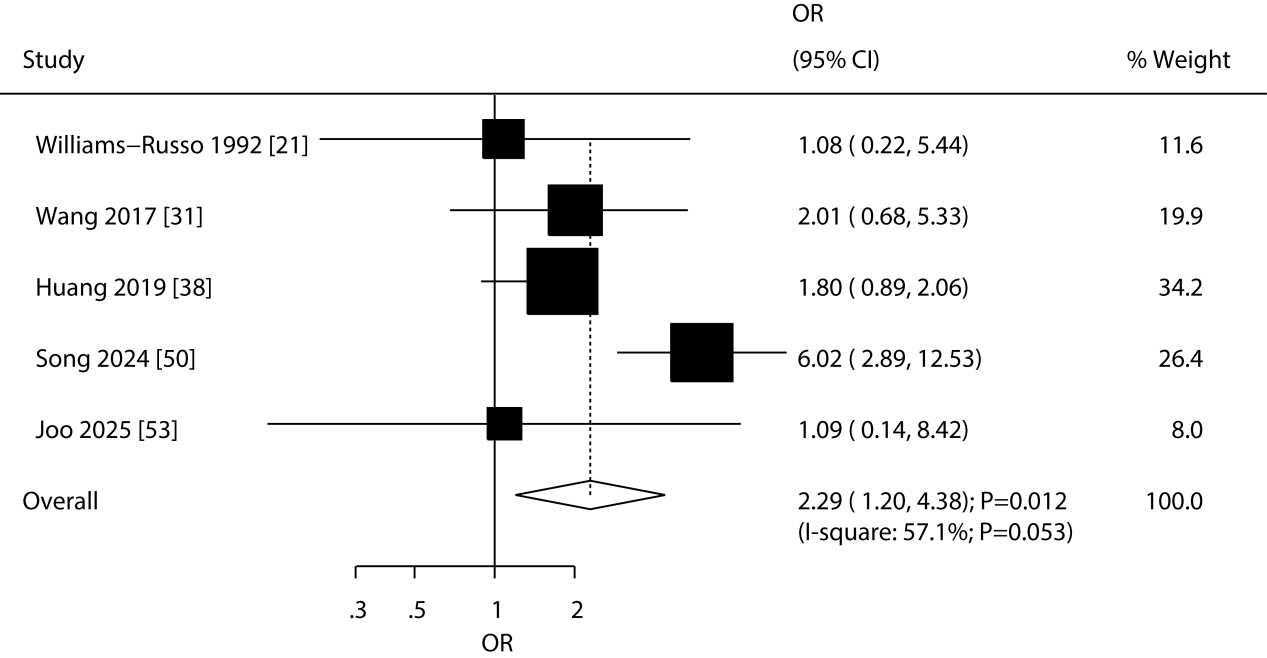


Figure S32. Association of COPD with the risk of POD


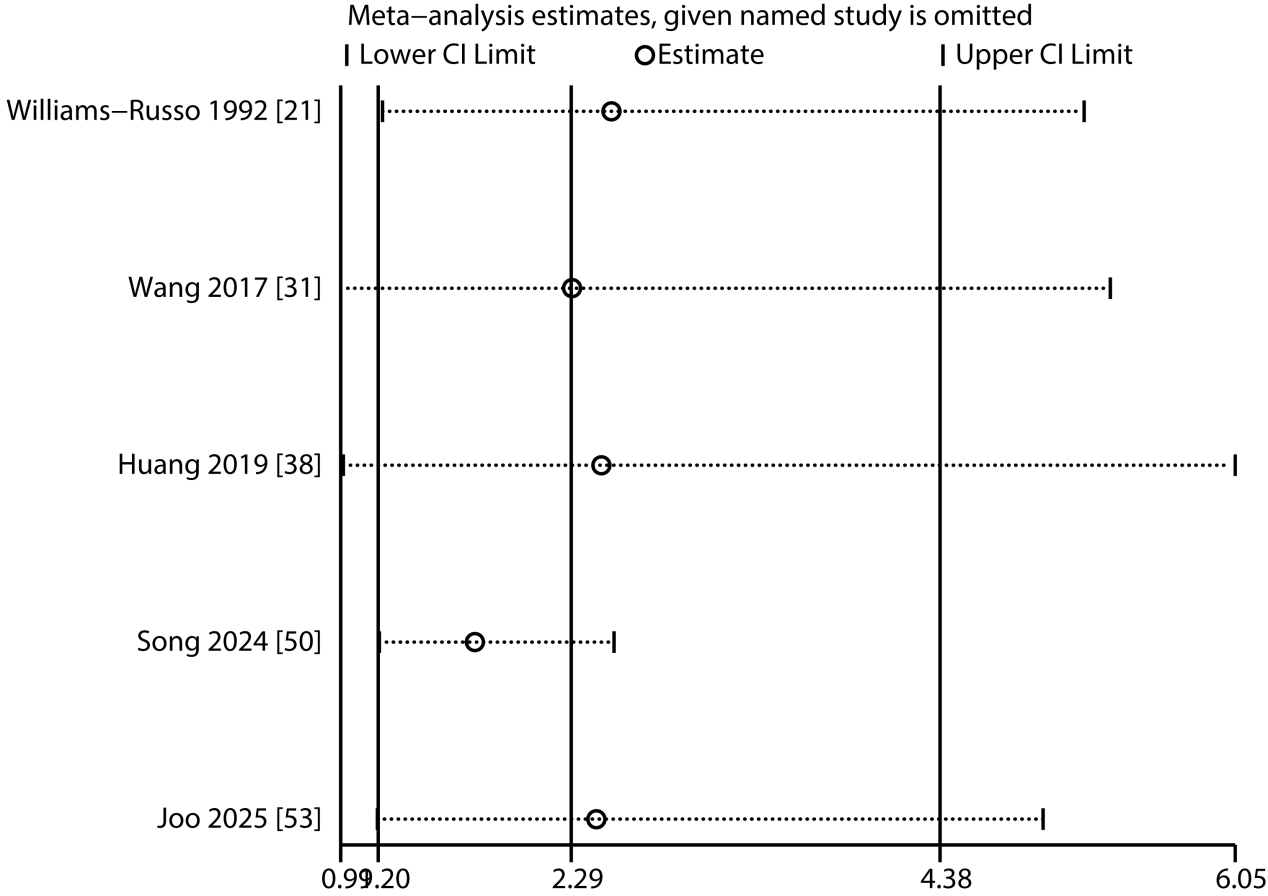


Figure S33. Sensitivity analysis regarding the association of COPD with the risk of POD


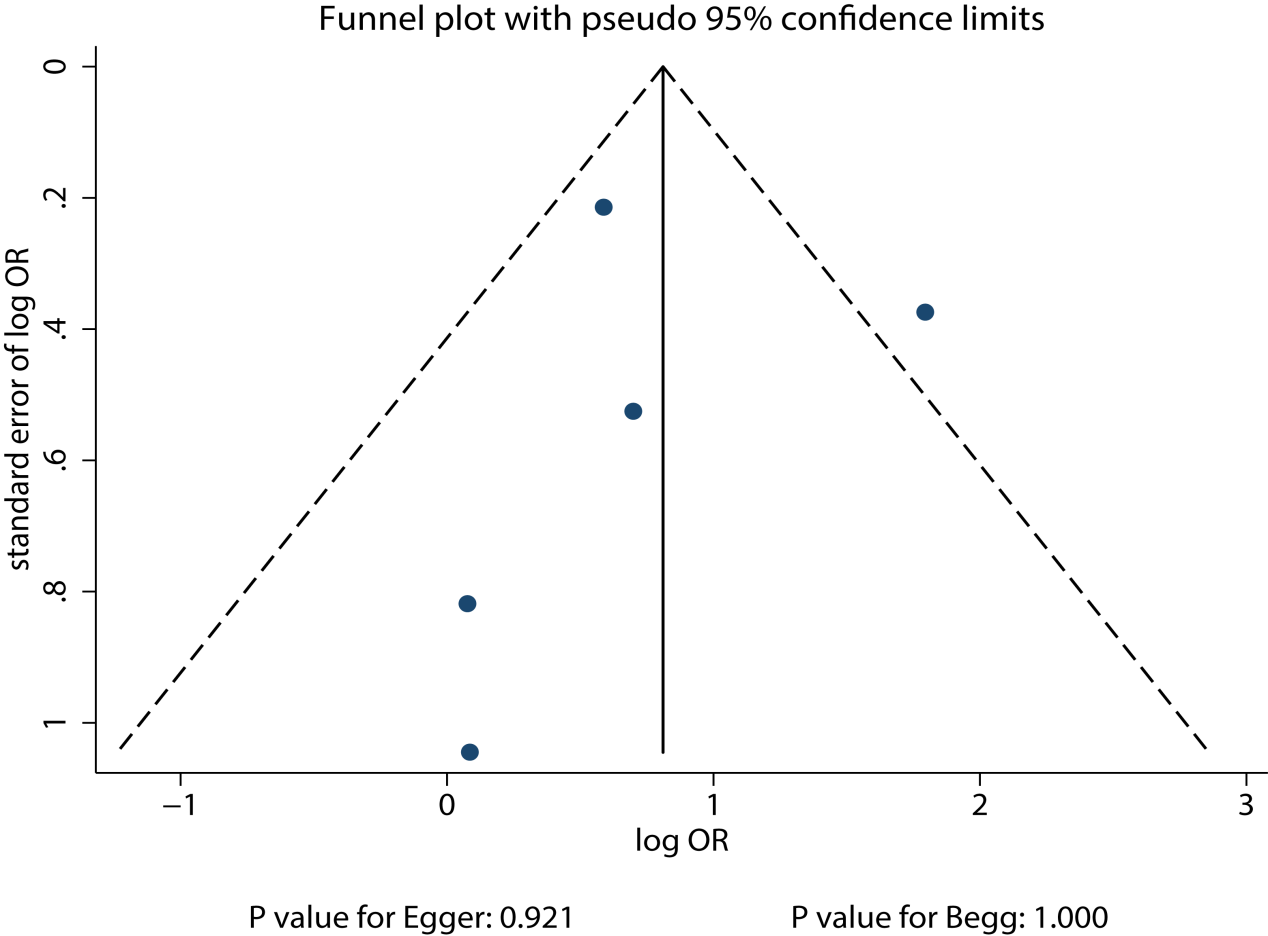


Figure S34. Funnel plot regarding the association of COPD with the risk of POD


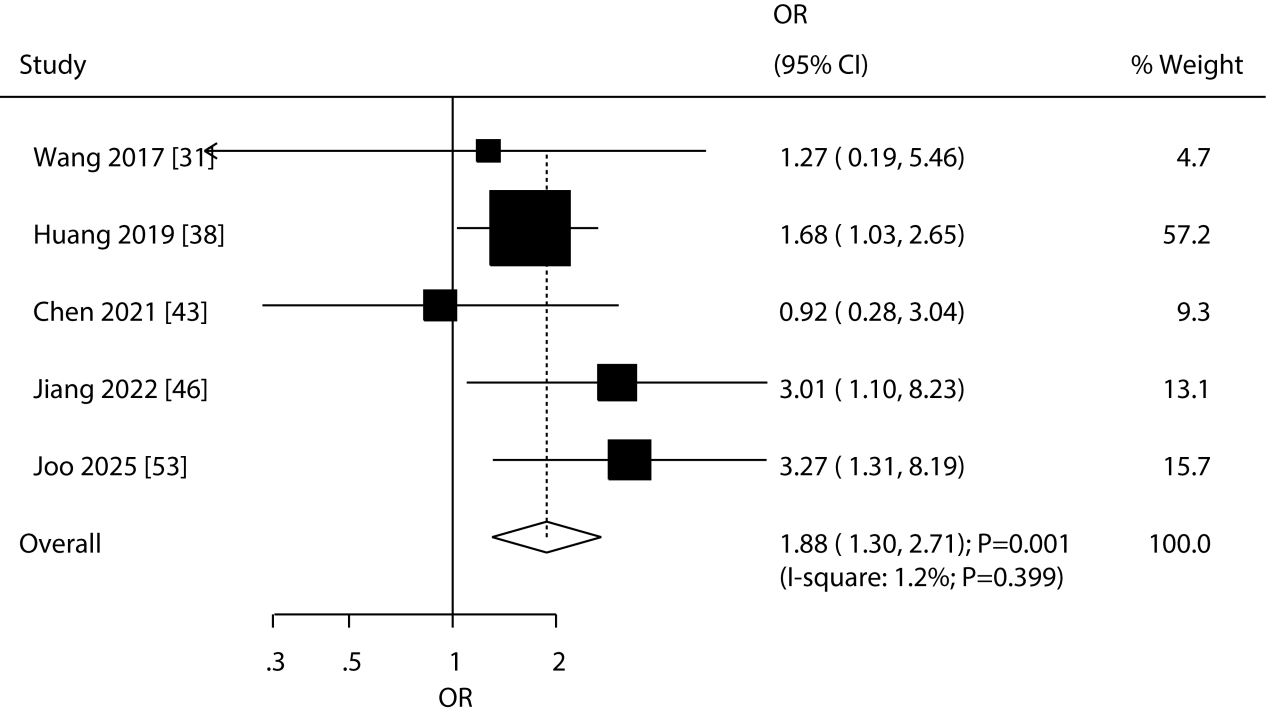


Figure S35. Association of renal disease with the risk of POD


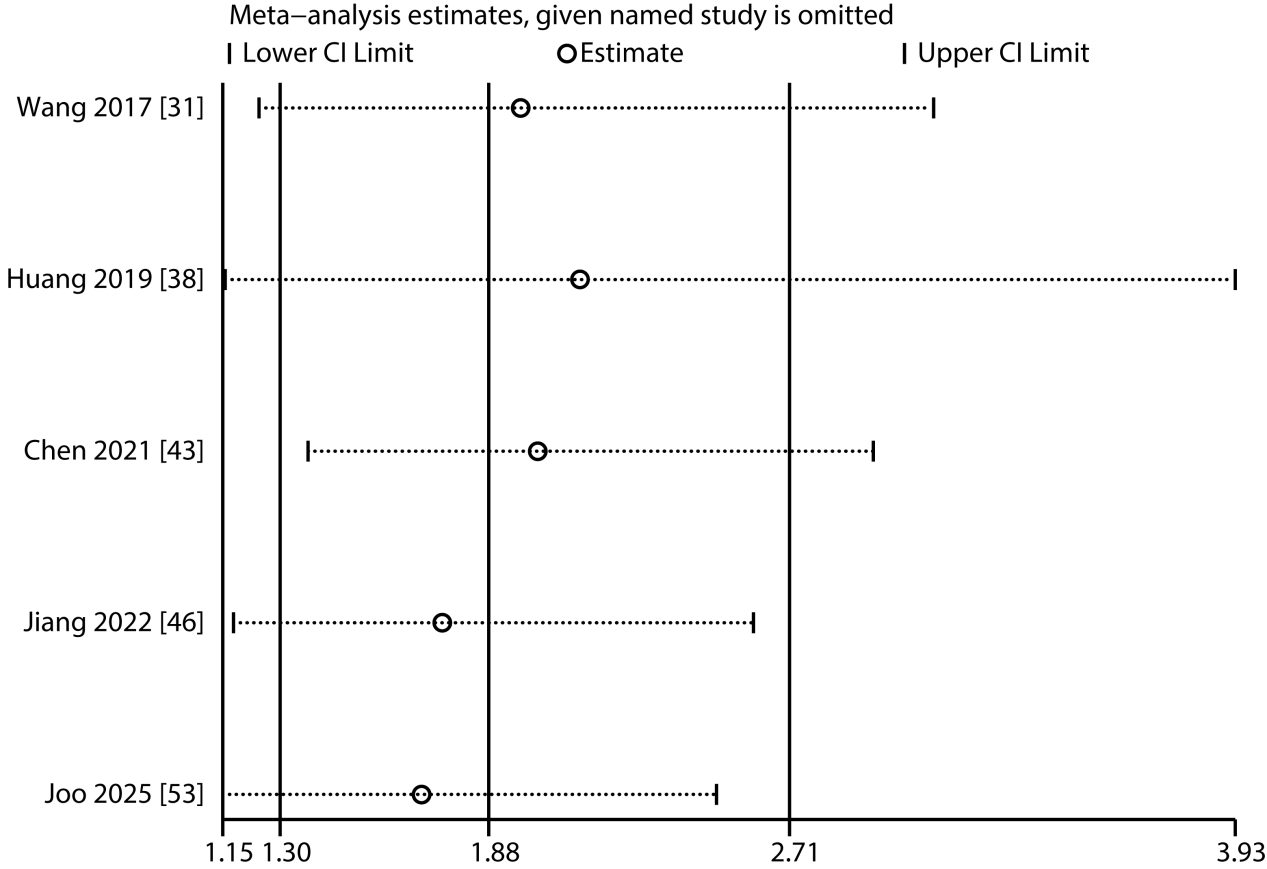


Figure S36. Sensitivity analysis regarding the association of renal disease with the risk of POD


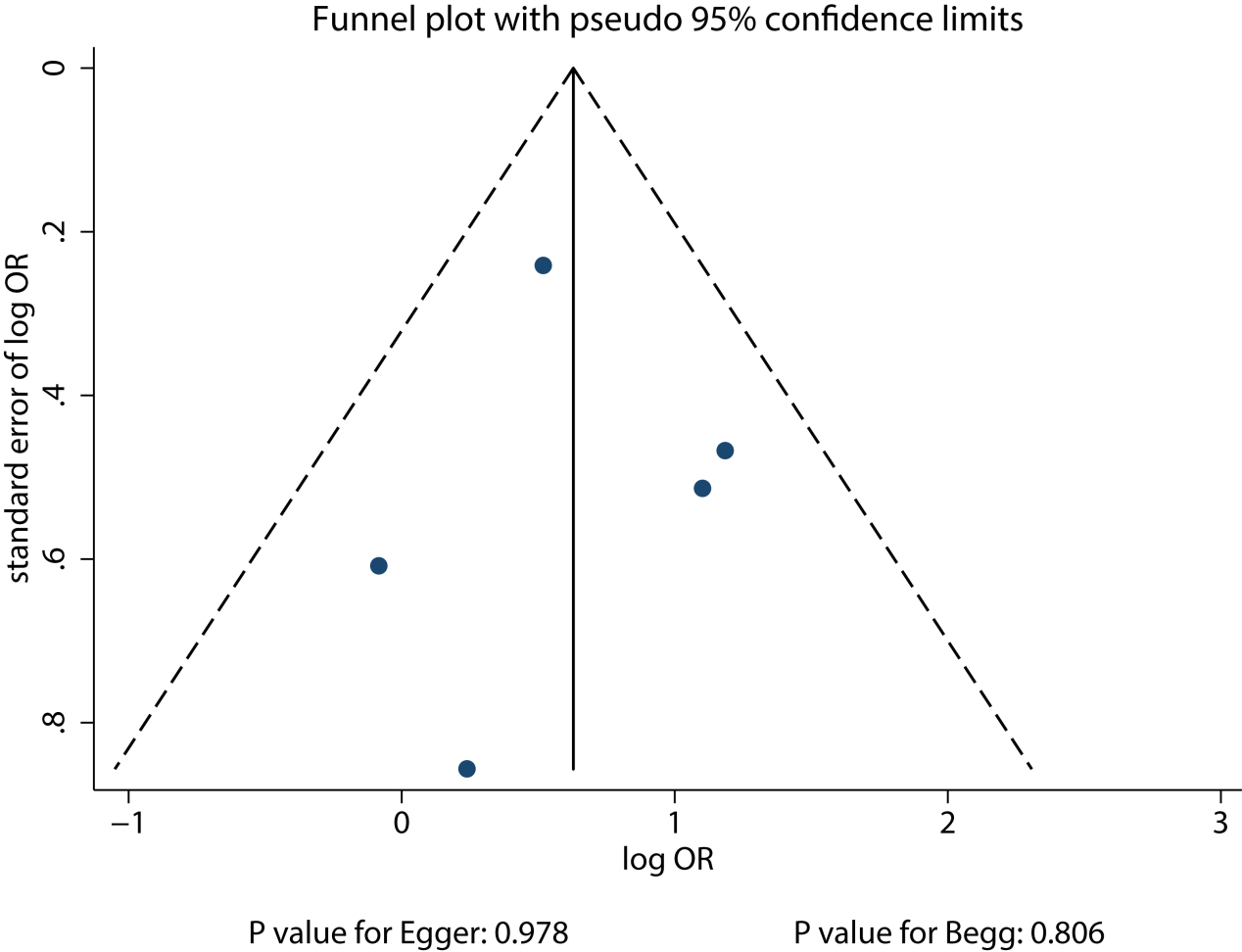


Figure S37. Funnel plot regarding the association of renal disease with the risk of POD


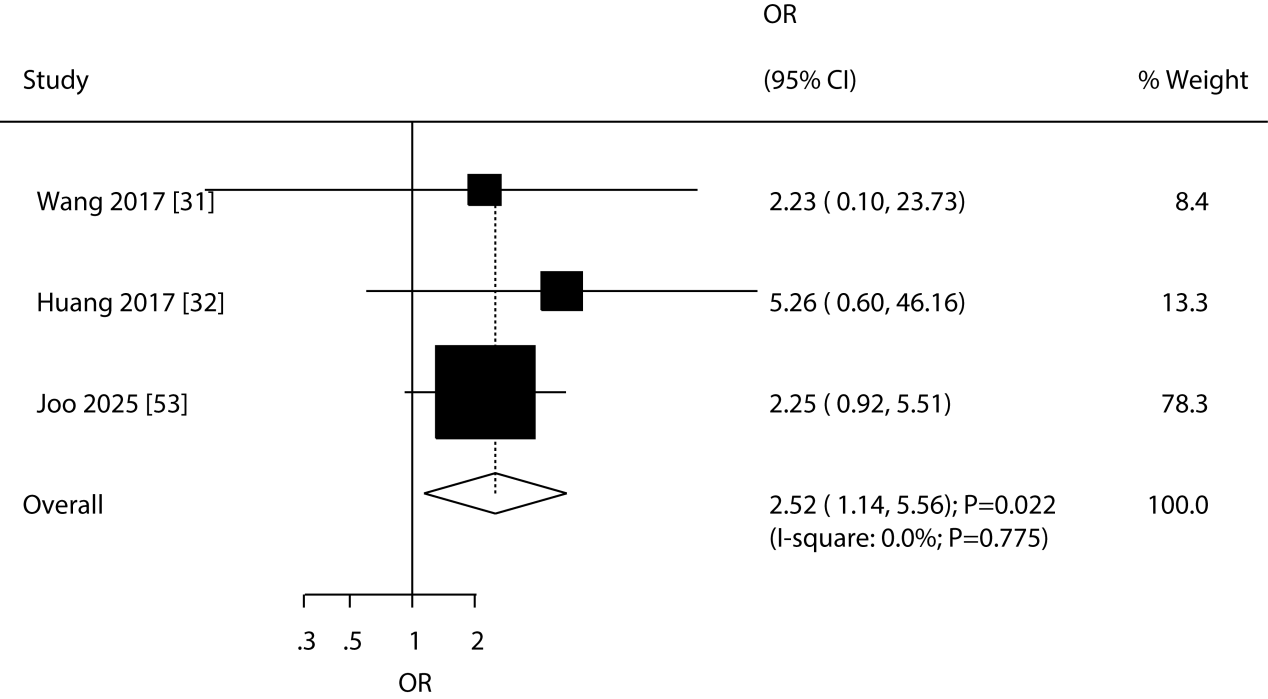


Figure S38. Association of solid tumor with the risk of POD


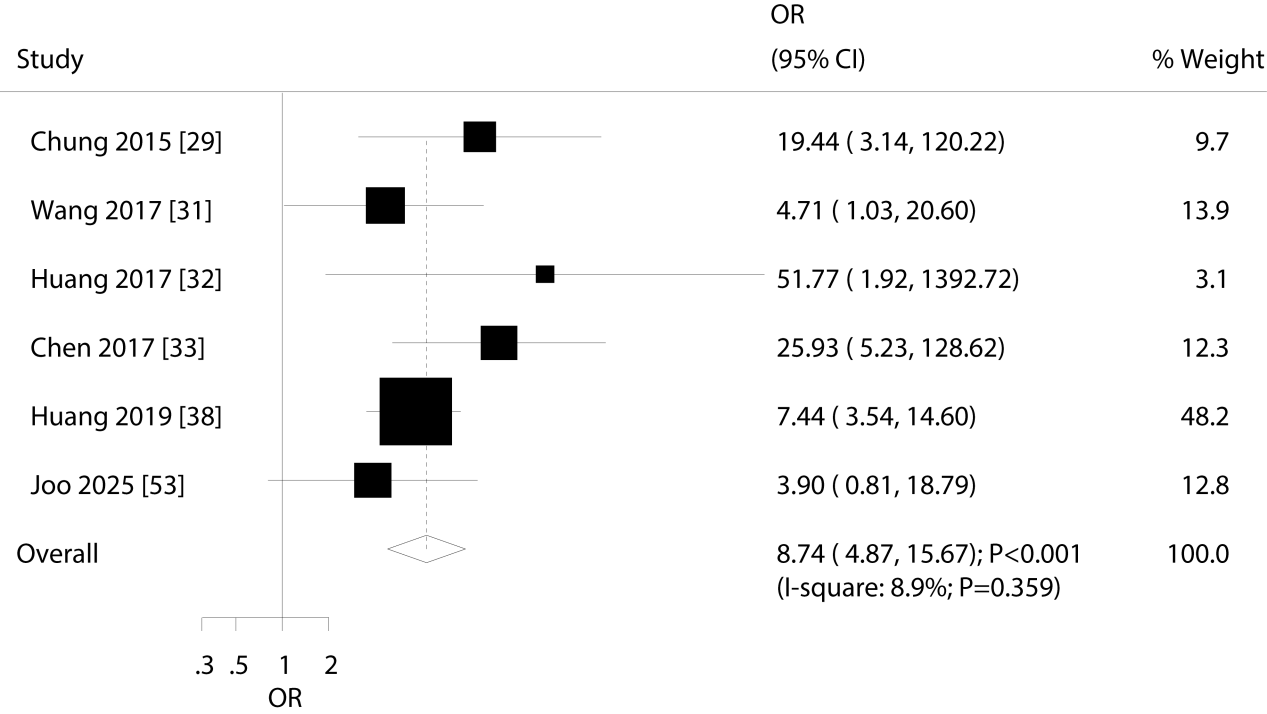


Figure S39. Association of dementia with the risk of POD


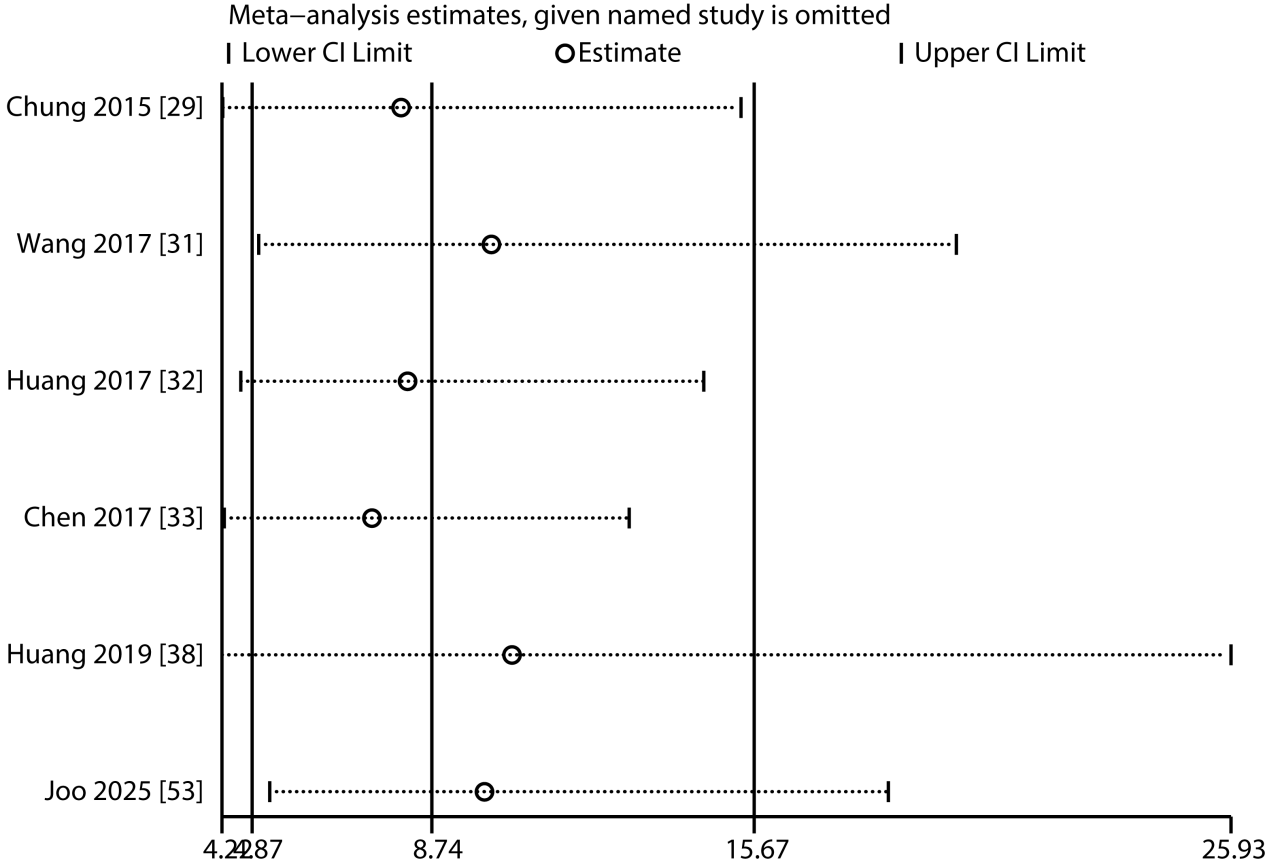


Figure S40. Sensitivity analysis regarding the association of dementia with the risk of POD


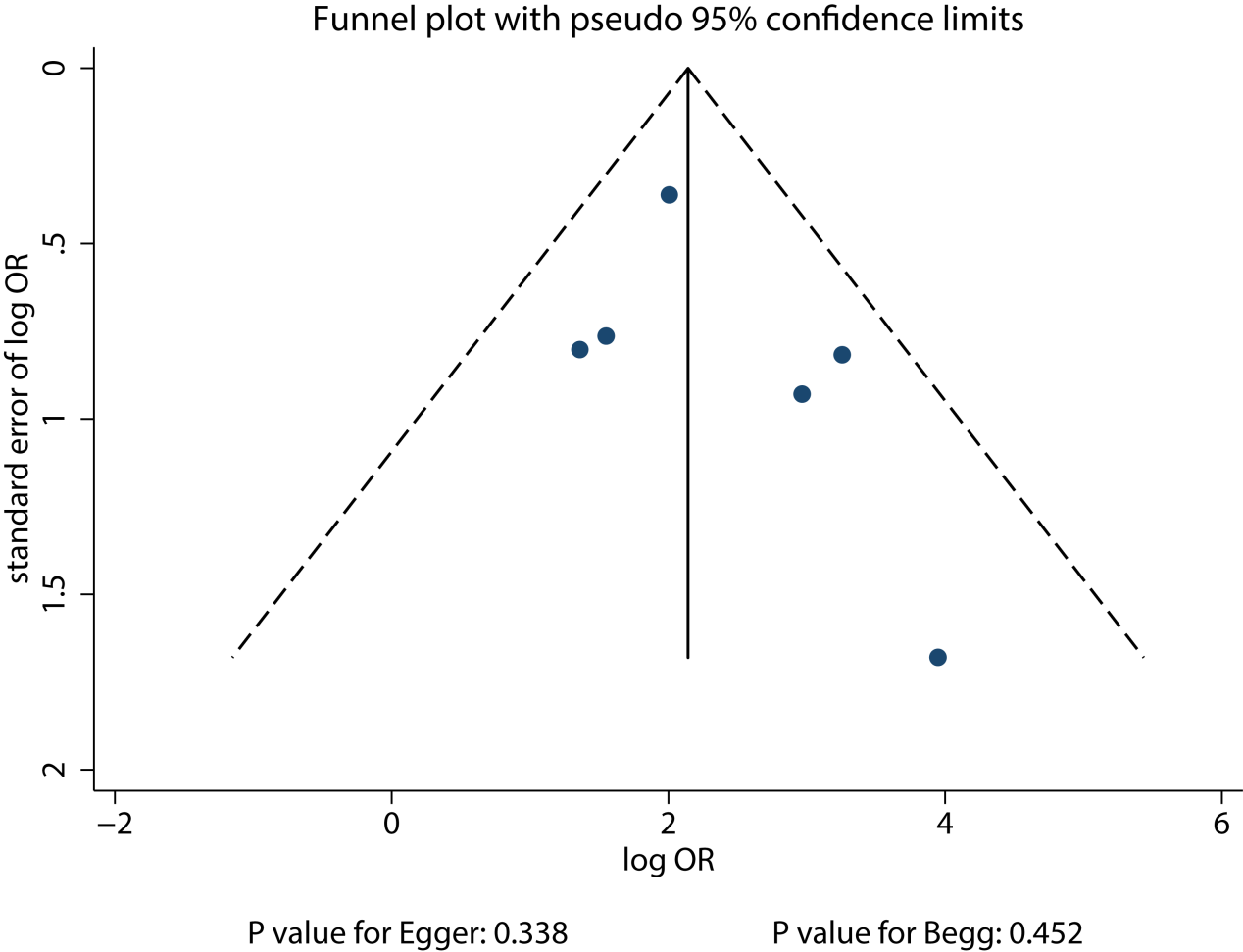


Figure S41. Funnel plot regarding the association of dementia with the risk of POD


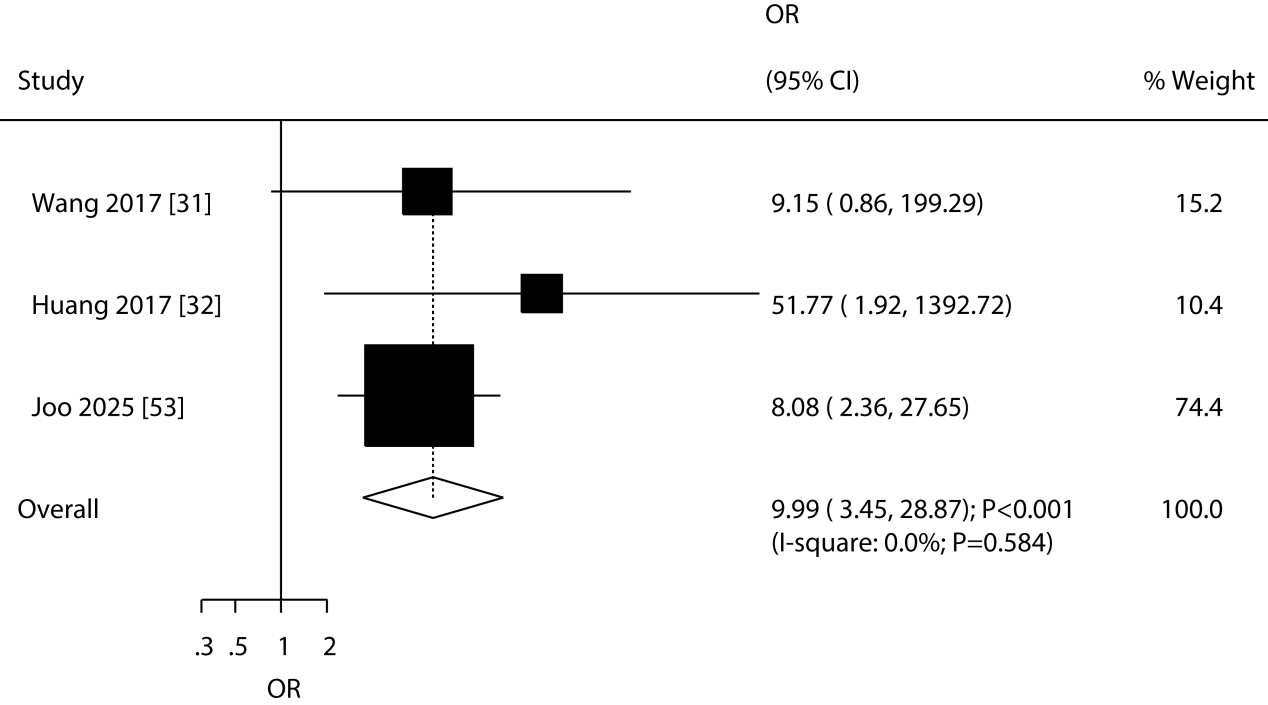


Figure S42. Association of Parkinson disease with the risk of POD


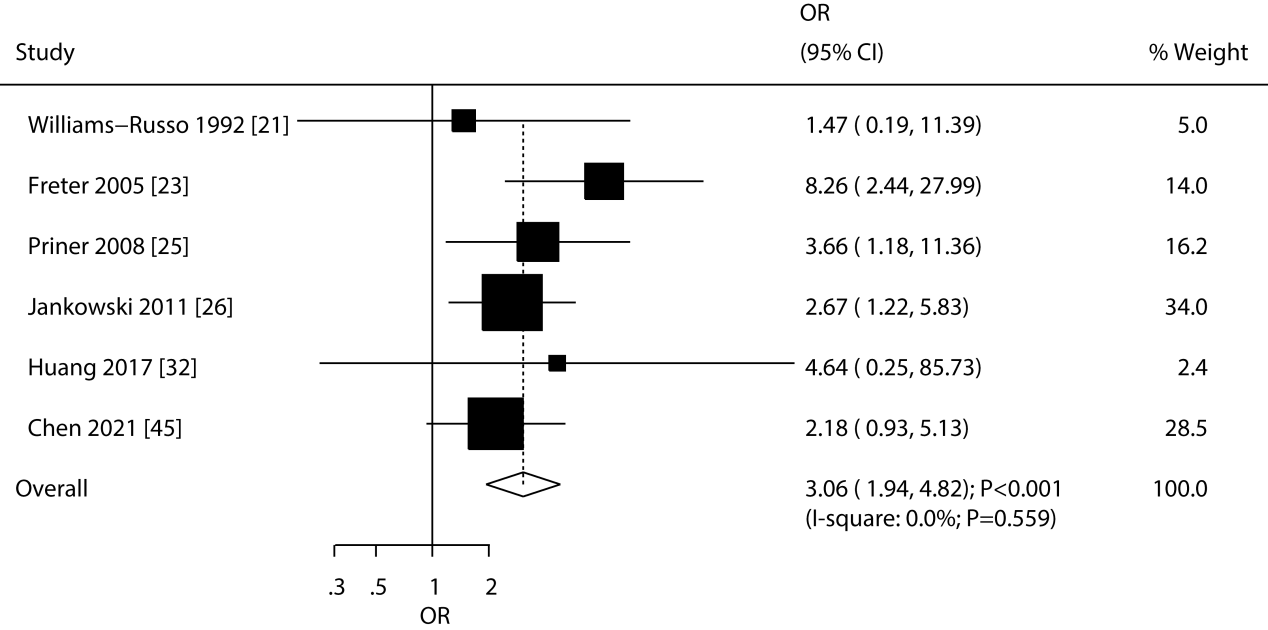


Figure S43. Association of psychiatric disease with the risk of POD


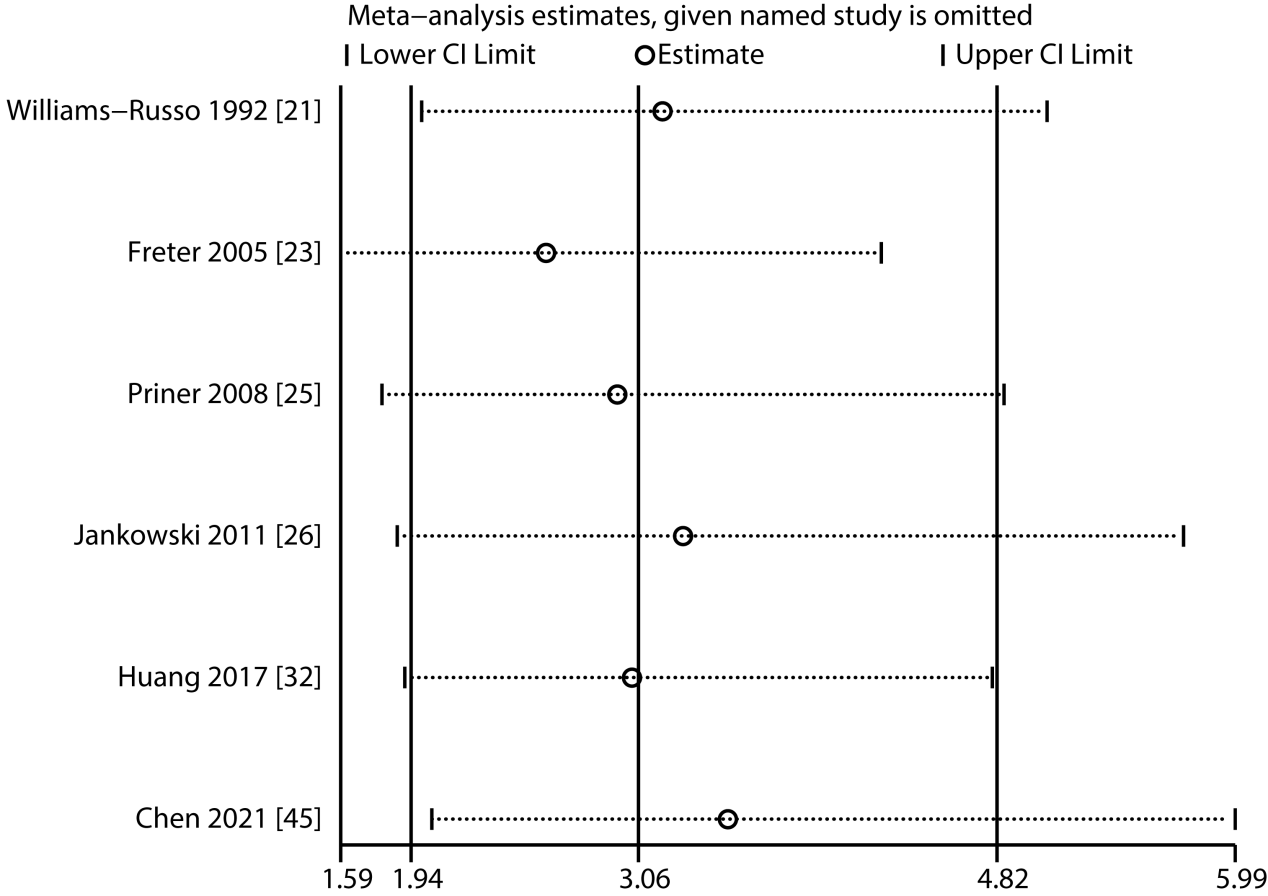


Figure S44. Sensitivity analysis regarding the association of psychiatric disease with the risk of POD


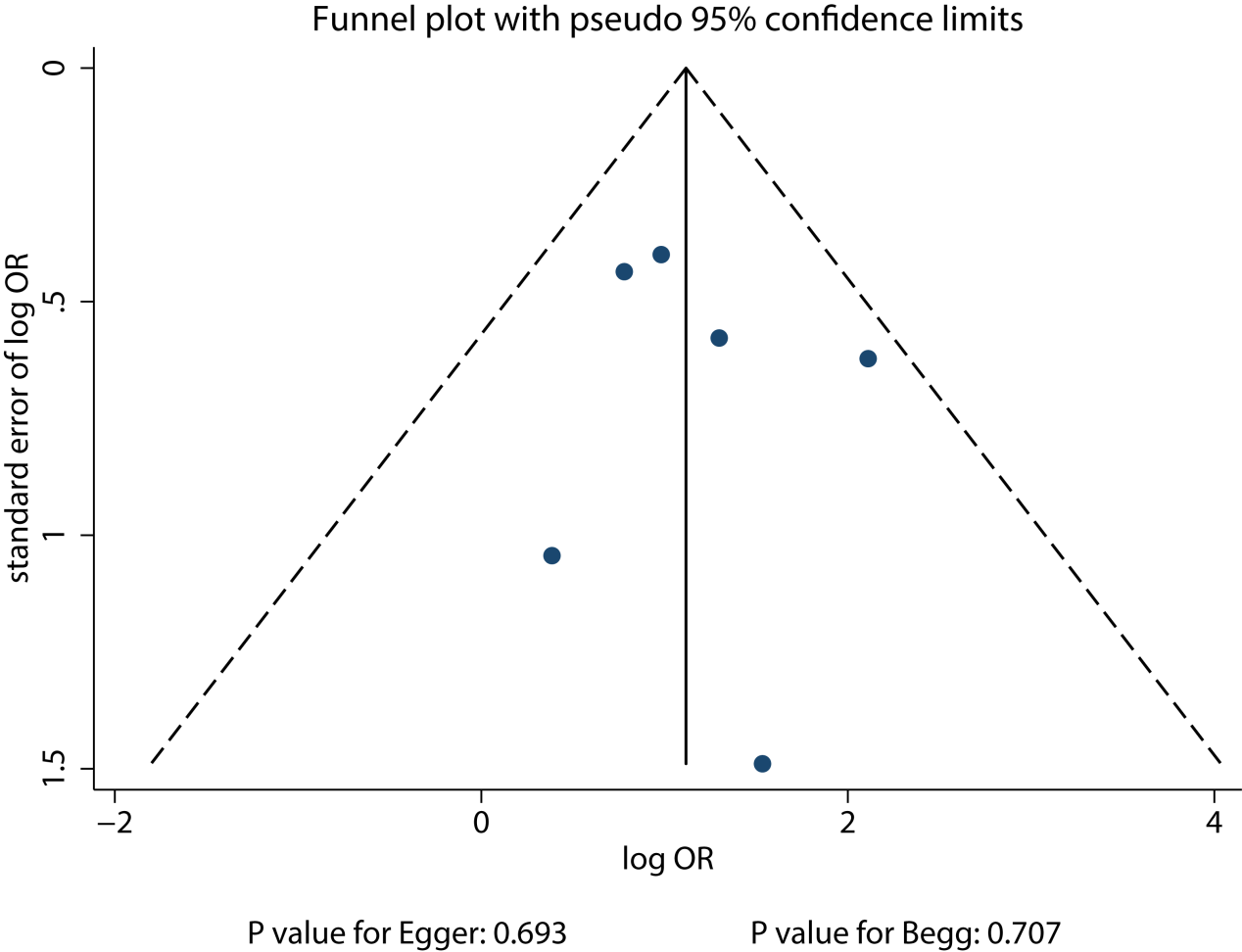


Figure S45. Funnel plot regarding the association of psychiatric disease with the risk of POD


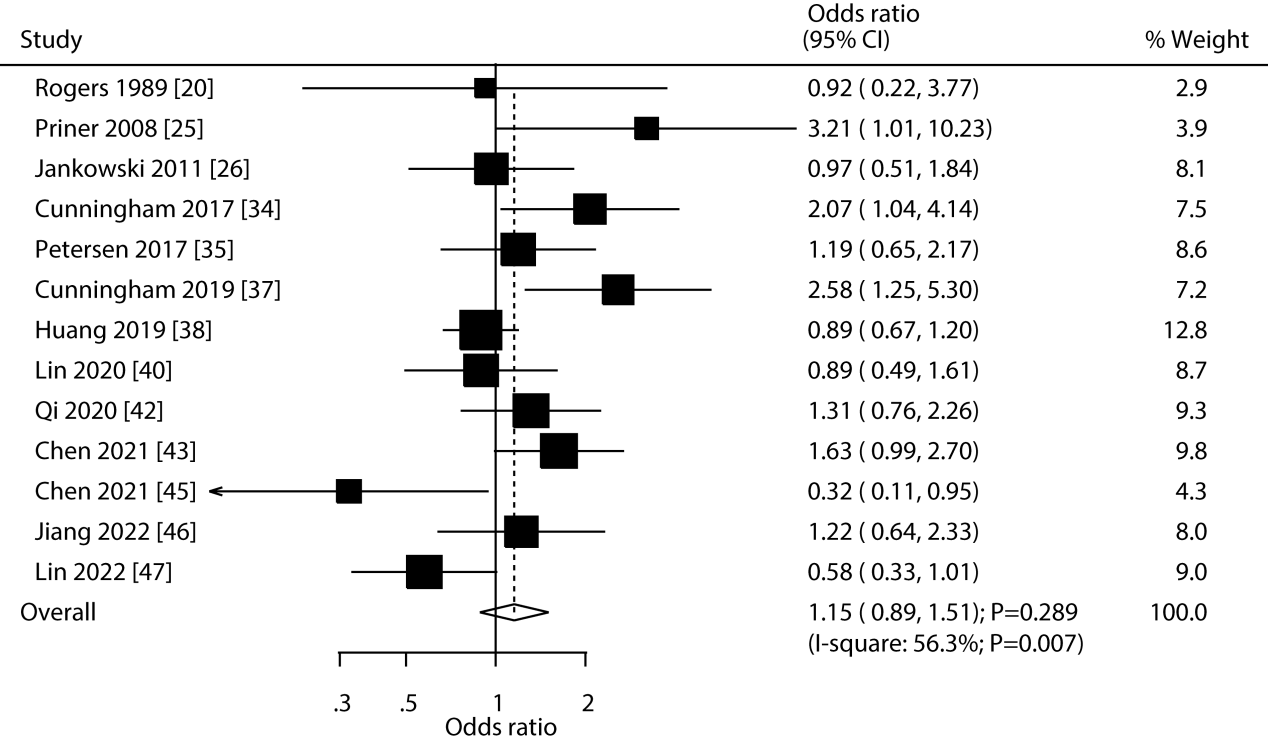


Figure S46. Association of surgical type (TKA vs THA) with the risk of POD


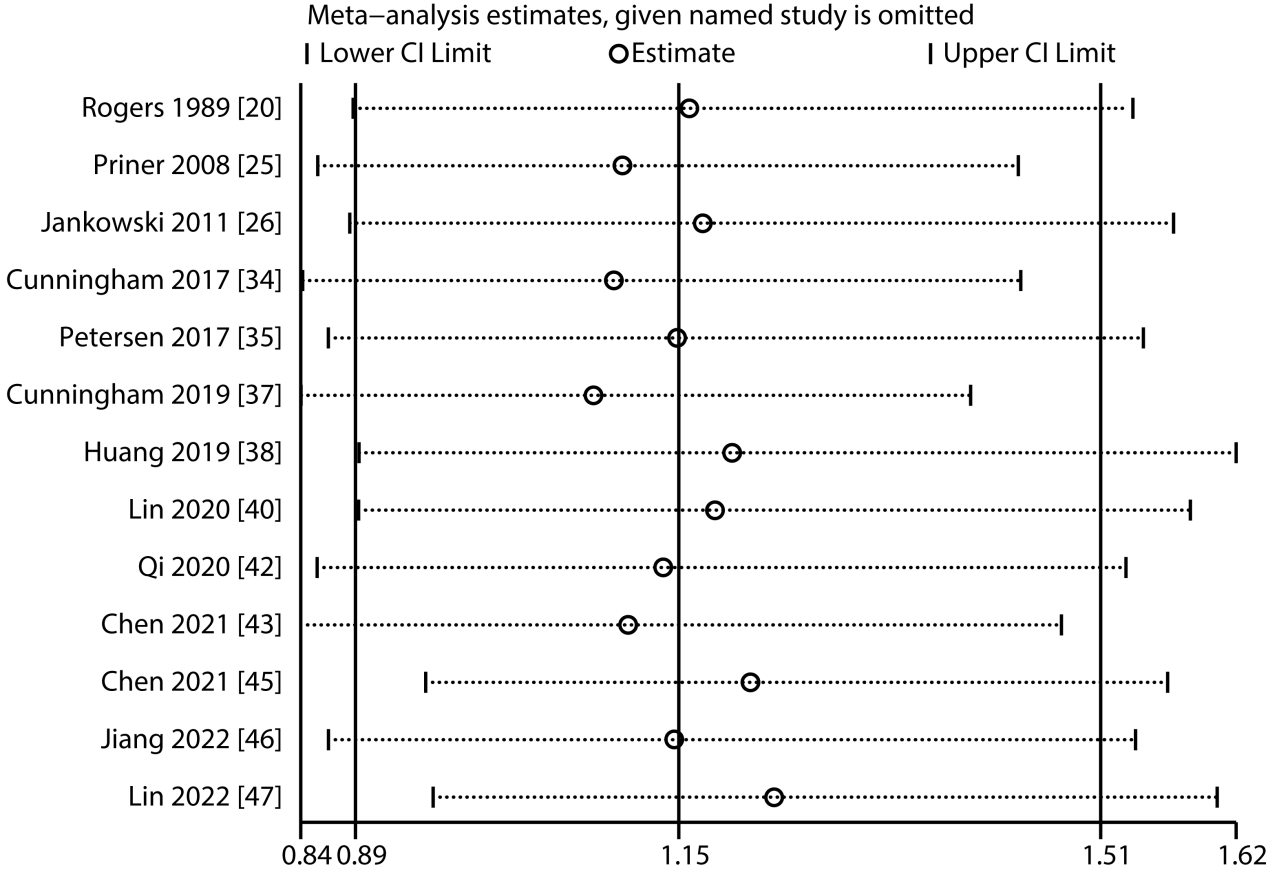


Figure S47. Sensitivity analysis regarding the association of surgical type (TKA vs THA) with the risk of POD


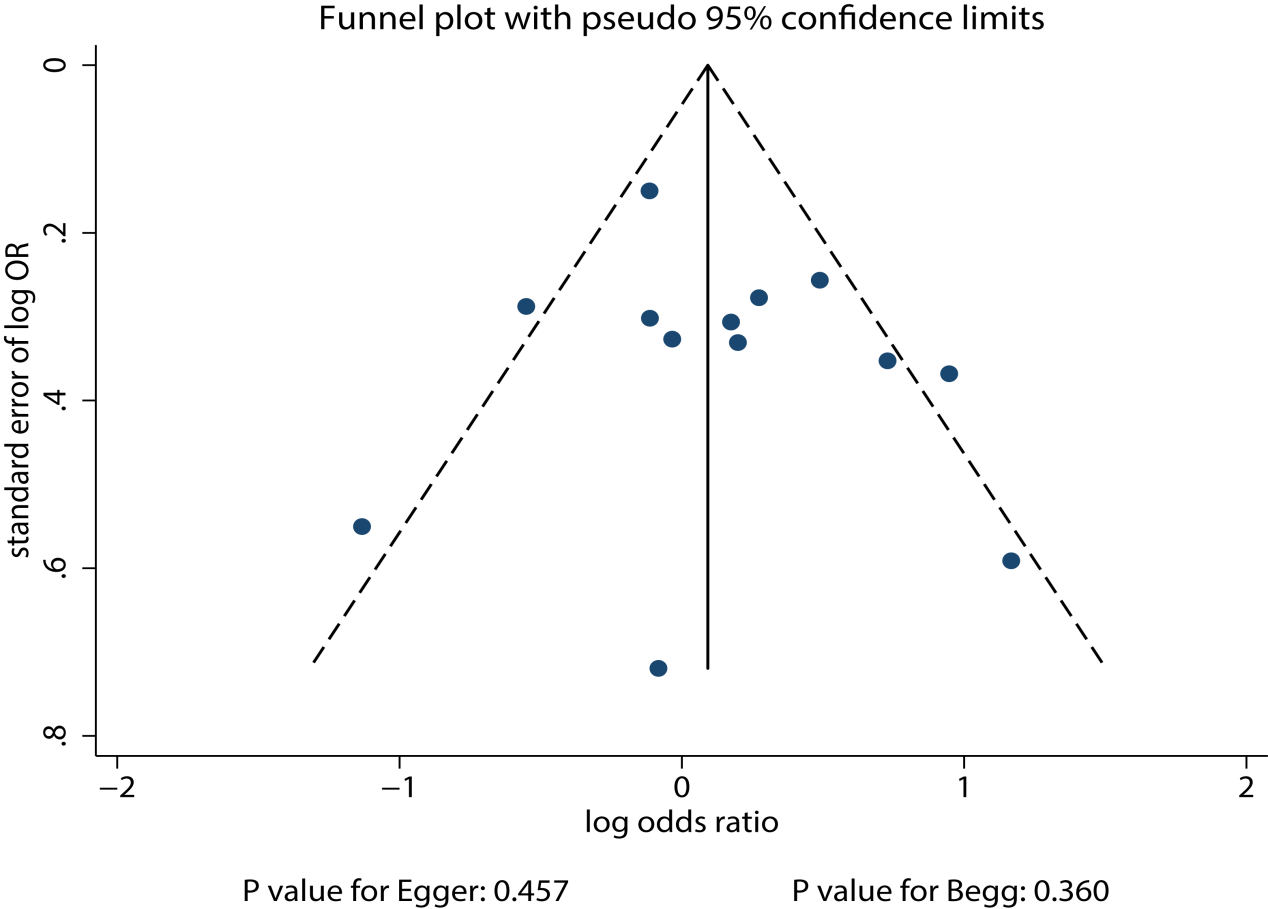


Figure S48. Funnel plot regarding the association of surgical type (TKA vs THA) with the risk of POD


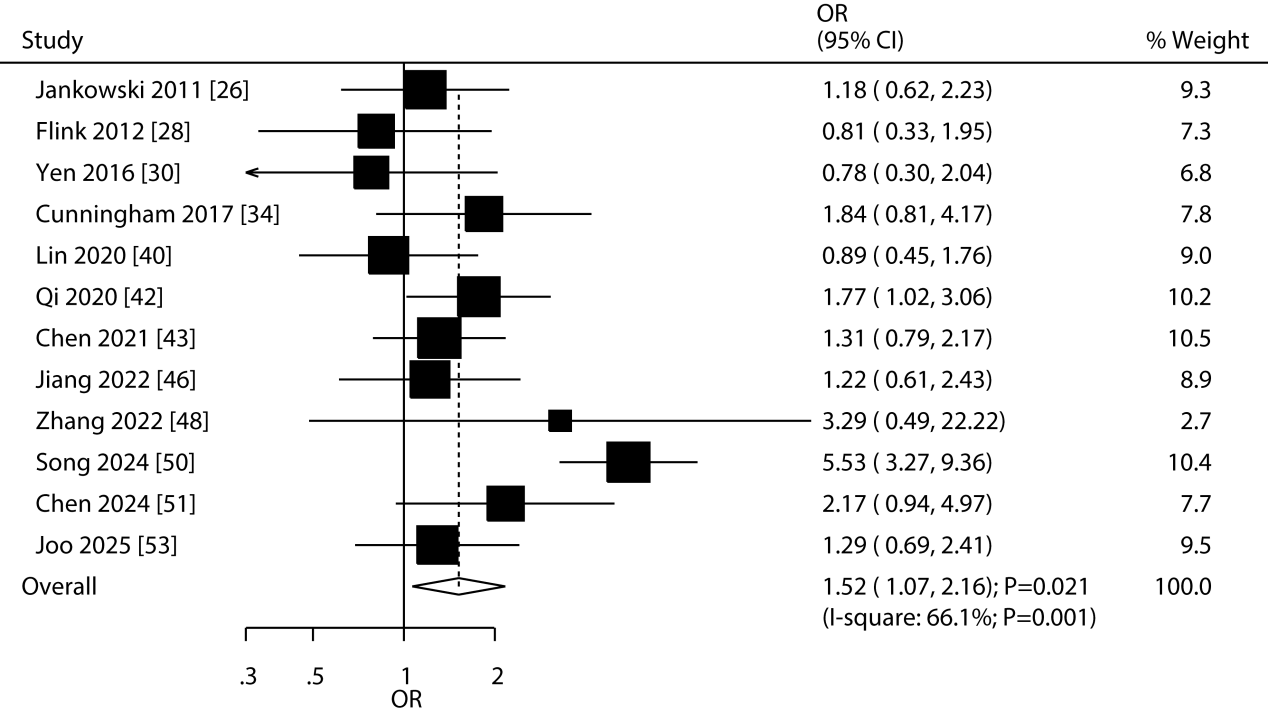


Figure S49. Association of ASA (III/IV vs I/II) with the risk of POD


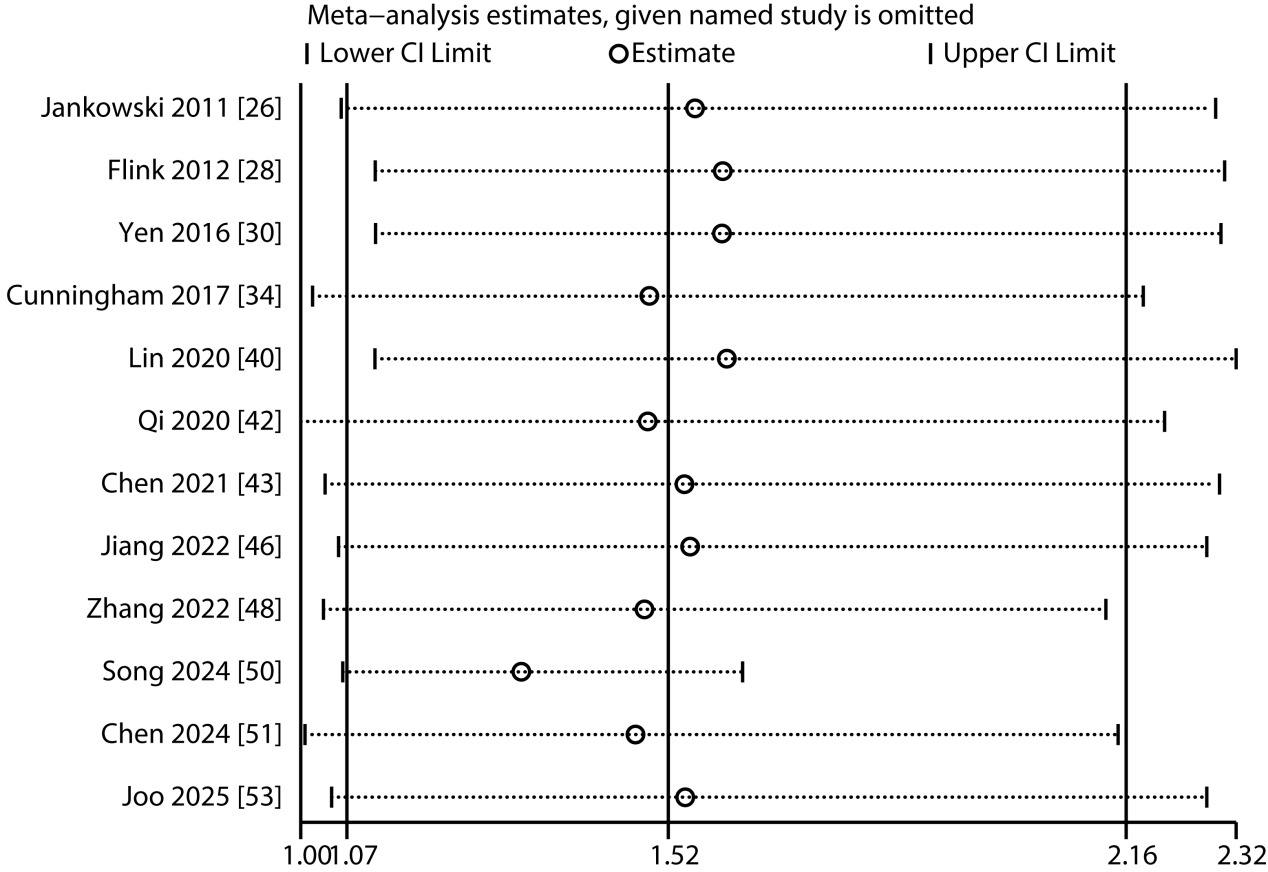


Figure S50. Sensitivity analysis regarding the association of ASA (III/IV vs I/II) with the risk of POD


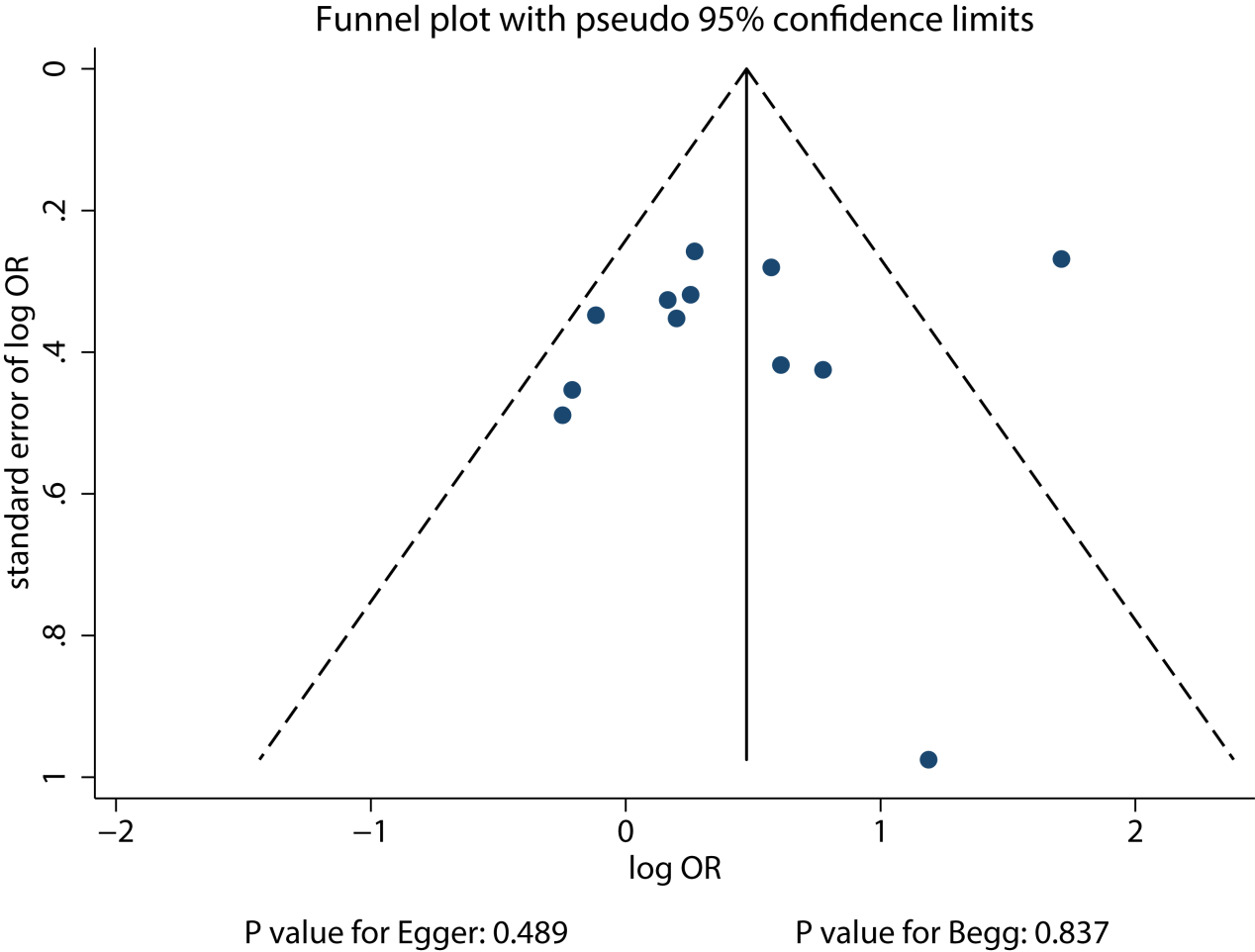


Figure S51. Funnel plot regarding the association of ASA (III/IV vs I/II) with the risk of POD


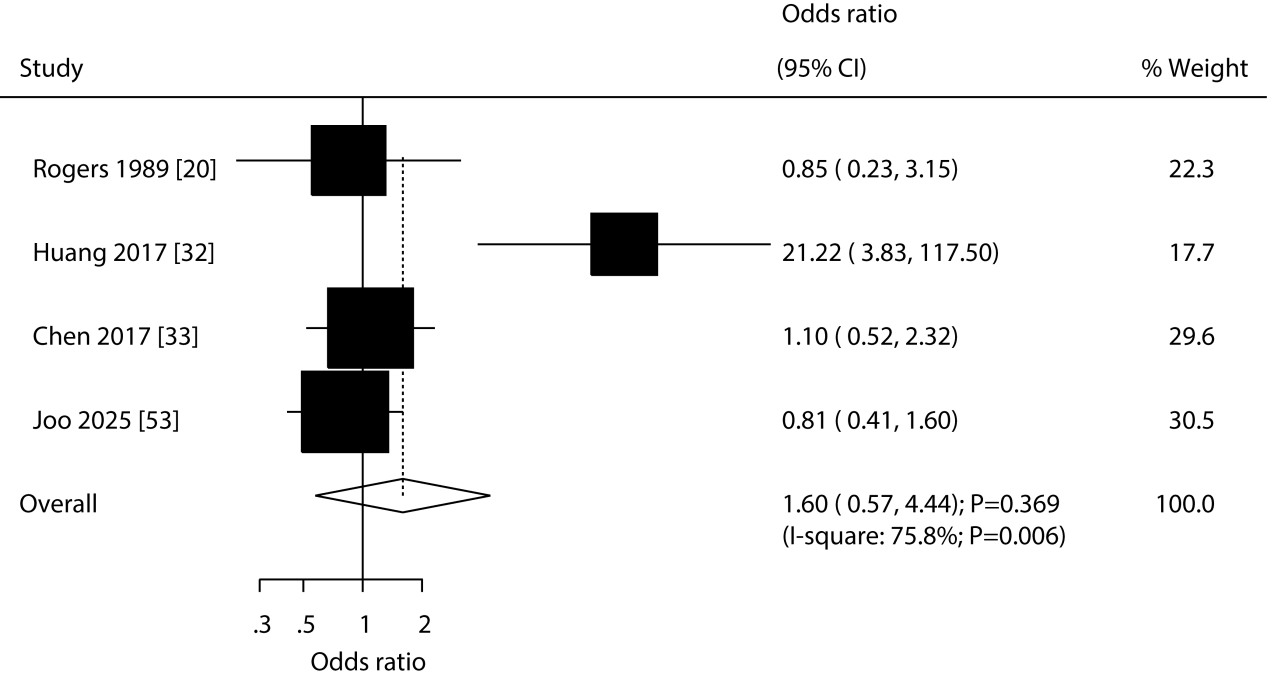


Figure S52. Association of opioid used with the risk of POD


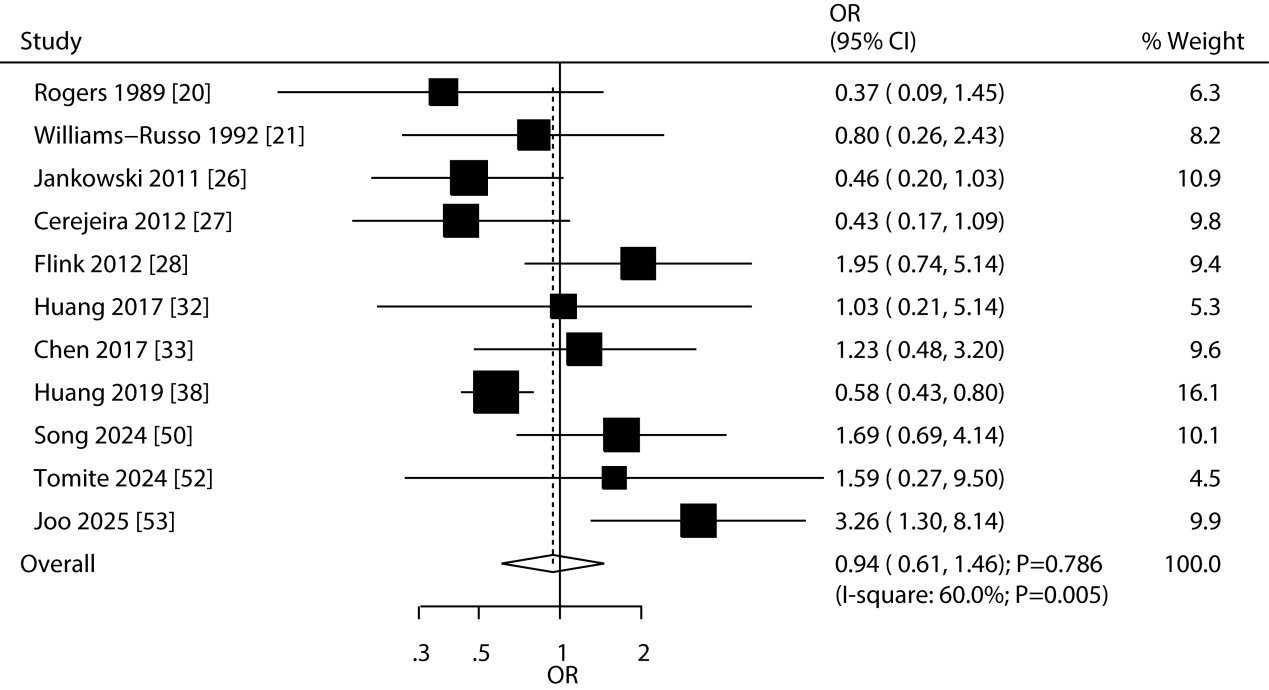


Figure S53. Association of anesthesia type (spinal vs general) used with the risk of POD


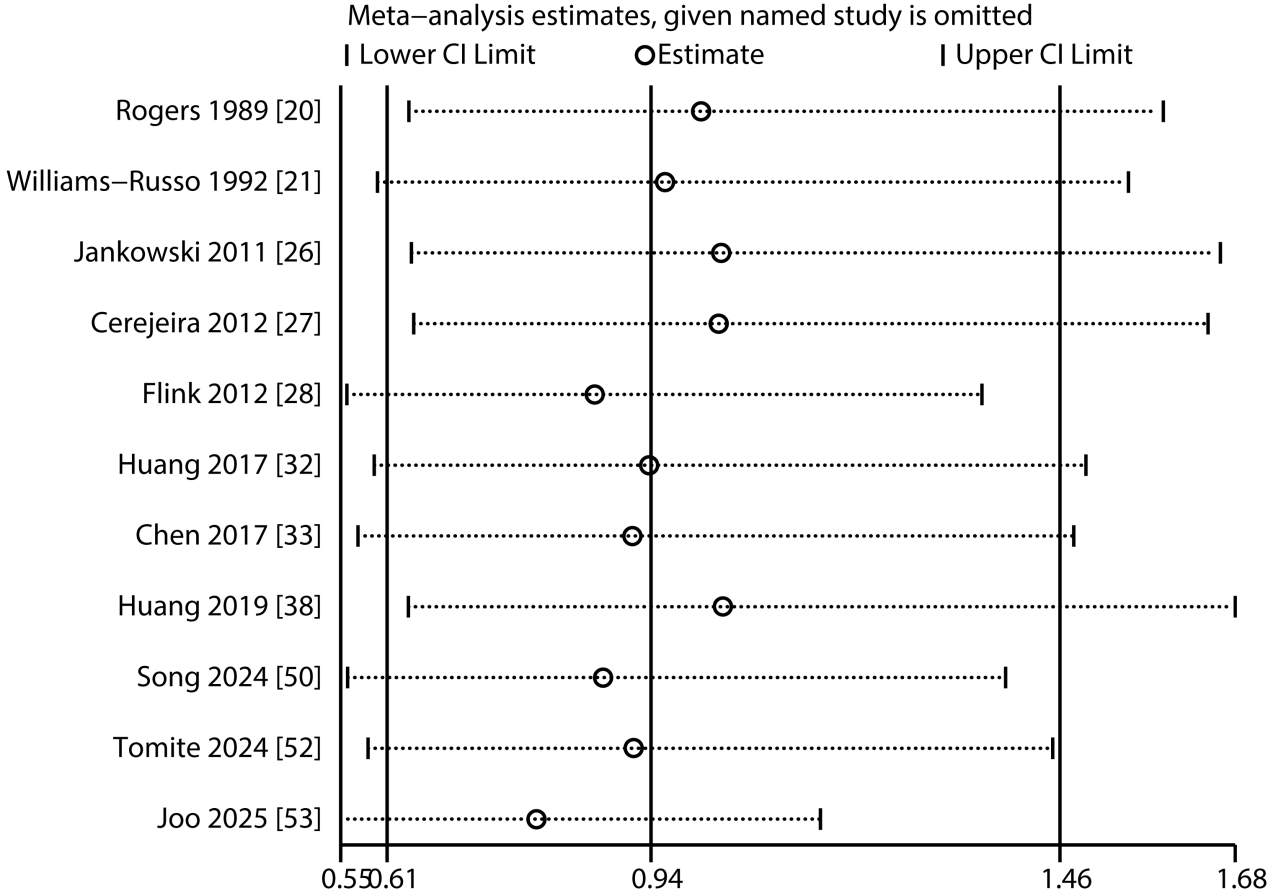


Figure S54. Sensitivity analysis regarding the association of anesthesia type (spinal vs general) used with the risk of POD


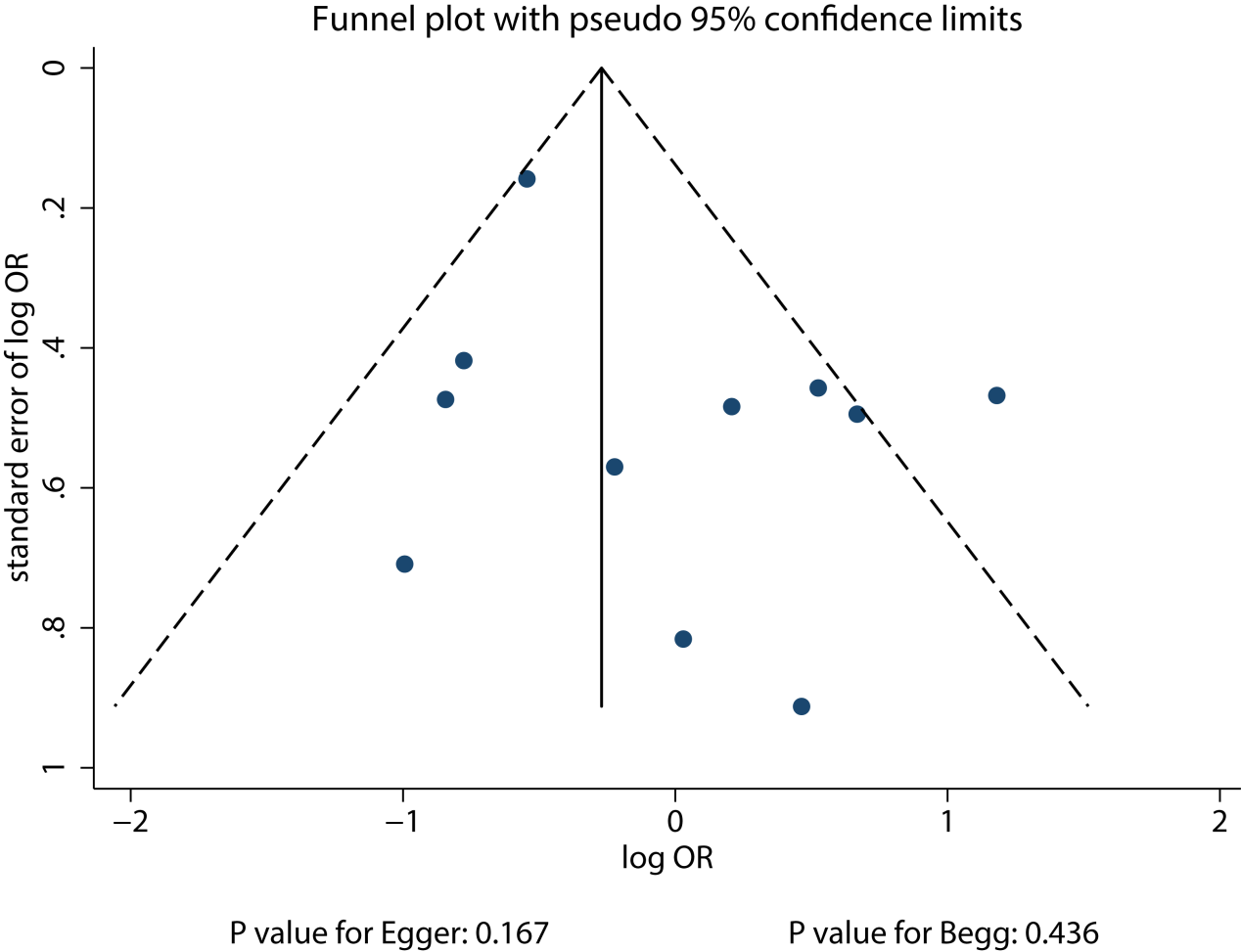


Figure S55. Funnel plot regarding the association of anesthesia type (spinal vs general) used with the risk of POD


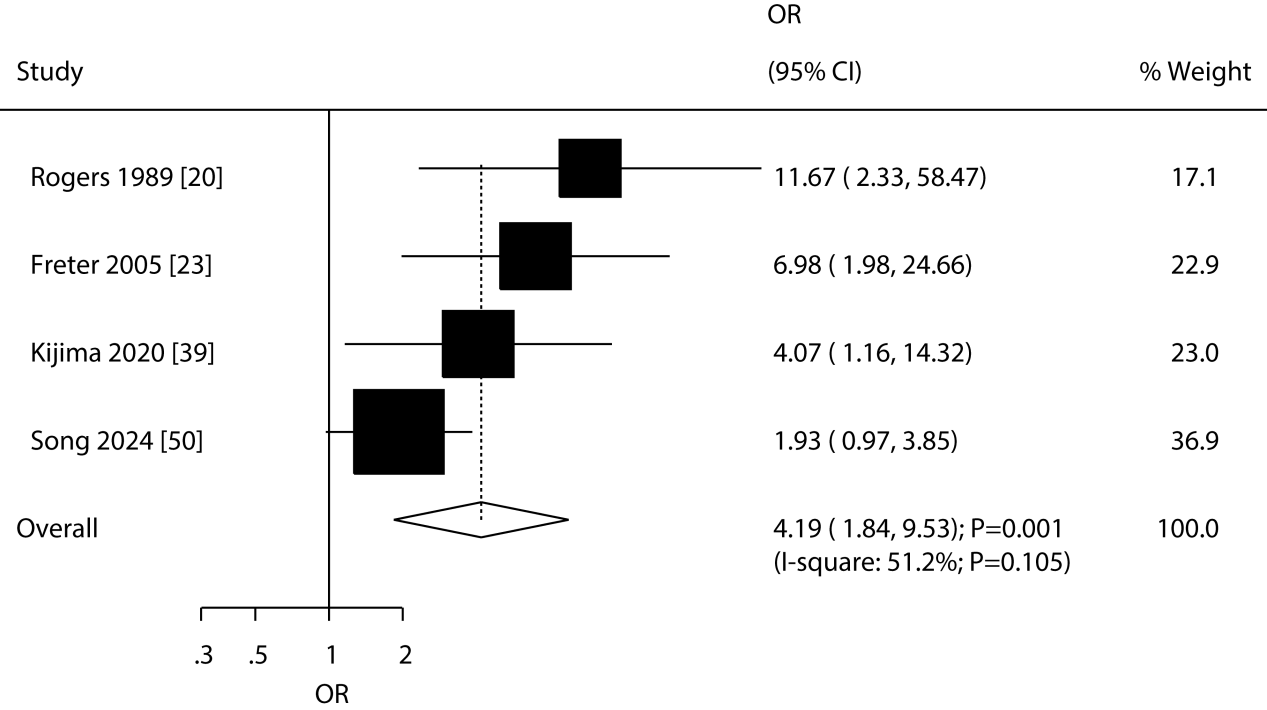


Figure S56. Association of substance use with the risk of POD


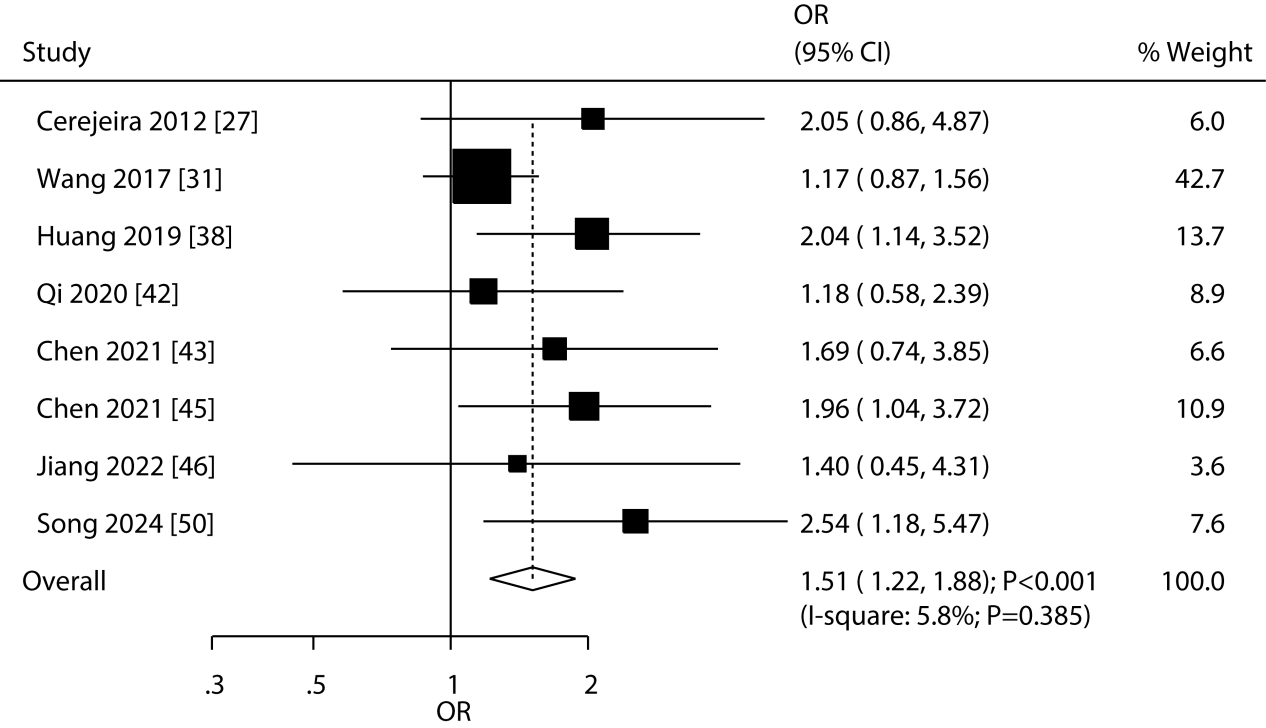


Figure S57. Association of transfusion with the risk of POD


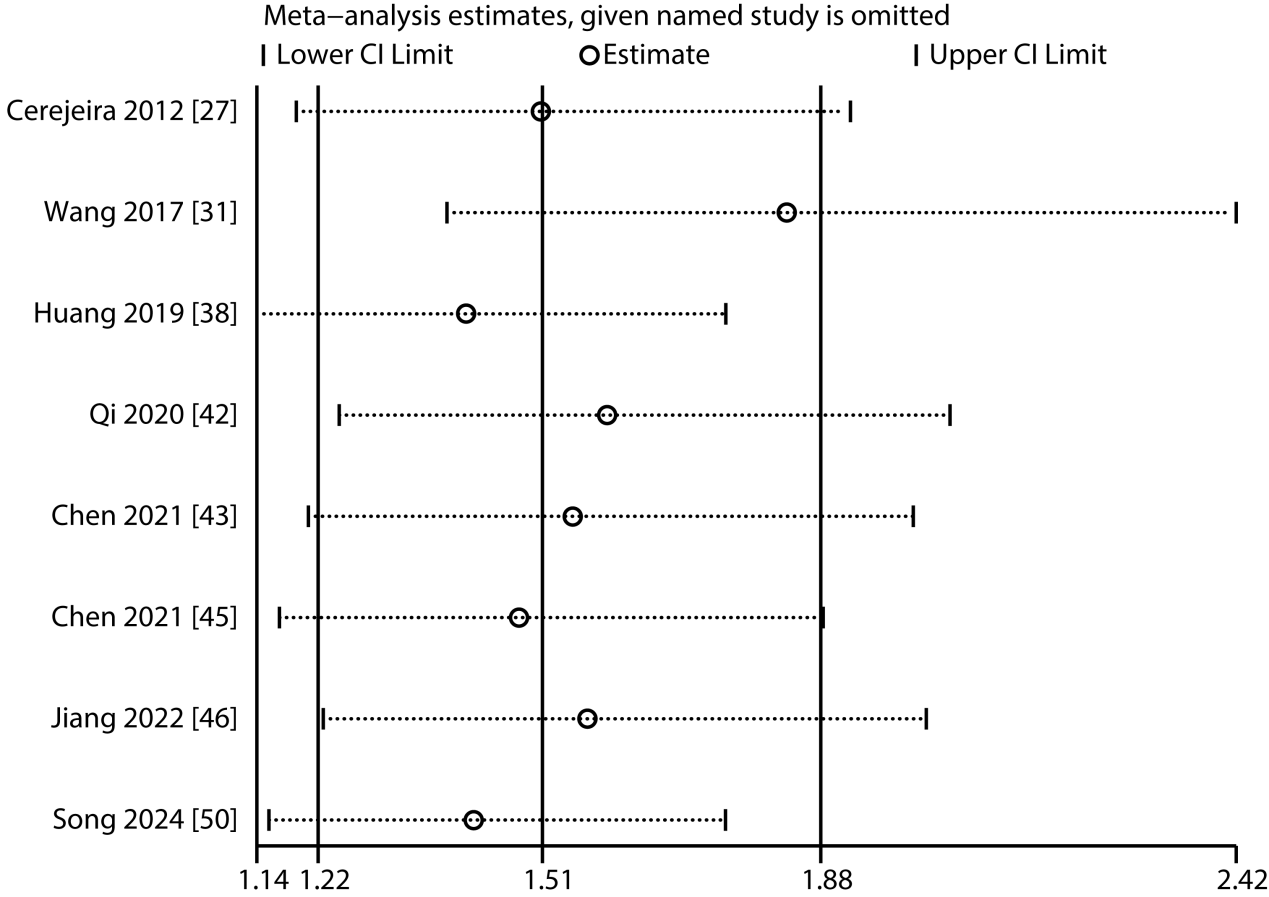


Figure S58. Sensitivity analysis regarding the association of transfusion with the risk of POD


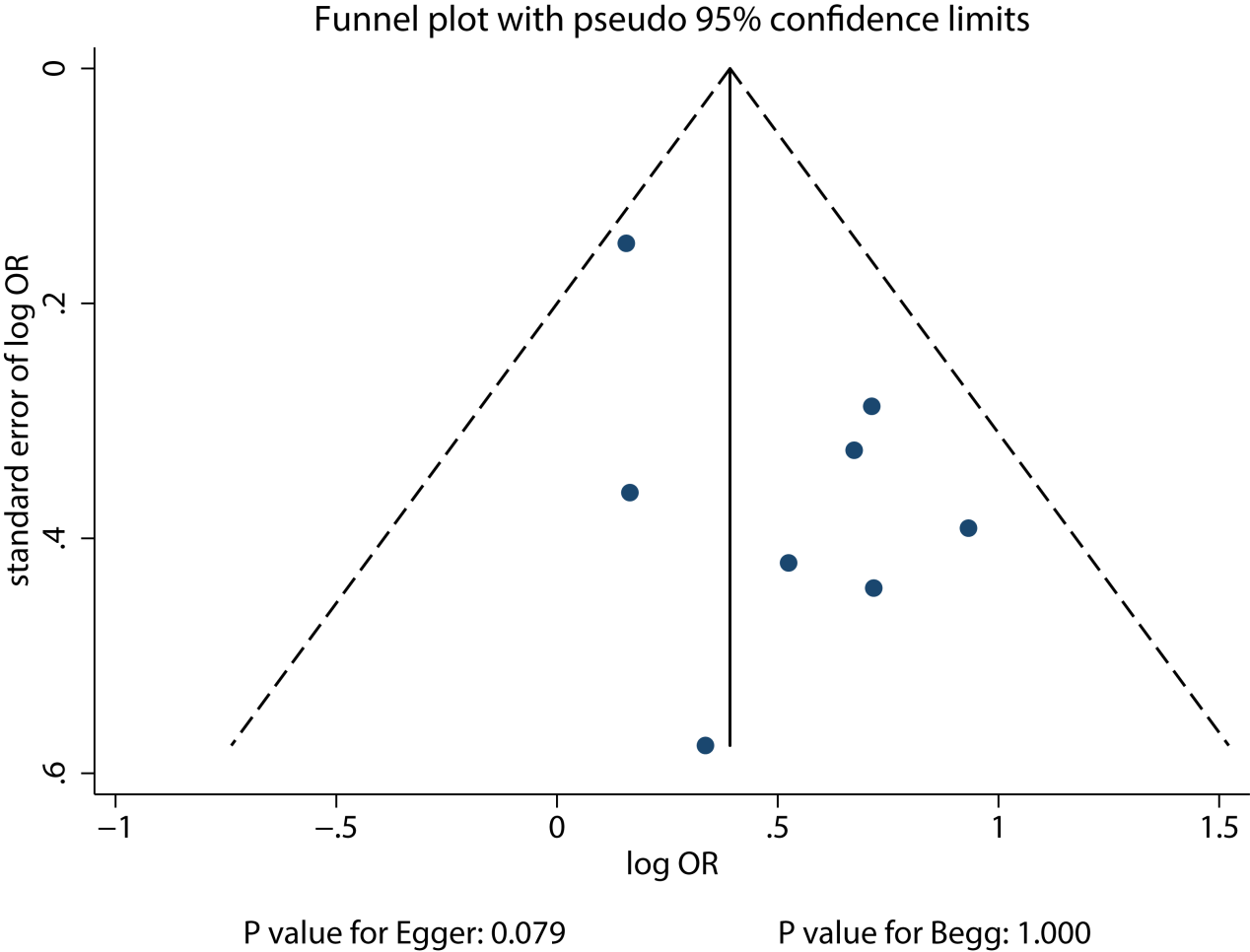


Figure S59. Funnel plot regarding the association of transfusion with the risk of POD
